# Regional and gender disparities in tobacco-related esophageal cancer: Insights from the Global Burden of Disease study 1990–2021

**DOI:** 10.18332/tid/205670

**Published:** 2025-07-19

**Authors:** Jinlong Chen, Zihan Qin, Xiaoxue Wang, Wei Jiang, Rui Gong, Xinyuan Liu, Kaiqi Yang, Peng Li, Shutian Zhang, Xiujing Sun, Jiugang Song

**Affiliations:** 1 Department of Gastroenterology, Beijing Friendship Hospital, Capital Medical University, Beijing, China; 2 State Key Laboratory of Digestive Health, Beijing, China; 3 National Clinical Research Center for Digestive Disease, Beijing, China; 4 Beijing Key Laboratory of Early Gastrointestinal Cancer Medicine and Medical Devices, Beijing, China; 5 Department of General Surgery, Beijing Friendship Hospital, Capital Medical University, Beijing, China

**Keywords:** tobacco-related esophageal cancer, epidemiology, disease burden, disability-adjusted life years, public health interventions

## Abstract

**INTRODUCTION:**

Tobacco-related esophageal cancer (TREC) is a major public health concern, with incidence and mortality rates rising globally. This study aims to analyze worldwide epidemiological data on TREC, examining its disease burden and temporal trends across regions, sexes, and age groups, in order to provide a theoretical basis for the development of targeted prevention policies.

**METHODS:**

This secondary dataset analysis utilized data from the Global Burden of Disease (GBD) 2021 study to examine the epidemiological features of TREC, focusing on mortality rates, disability-adjusted life years (DALYs), and other key indicators across regions and genders.

**RESULTS:**

From 1990 to 2021, global deaths due to TREC increased from 143332.8 to 219185.3, while the age-standardized death rate (ASDR) decreased from 3.6 to 2.5 per 100000 persons. The rise in TREC burden was primarily attributed to relative contributions from population growth (154.62%) and aging (39.75%). DALYs associated with TREC rose from 3844095.6 to 5136277, with a notable decline in age-standardized DALYs rate (ASDR) from 93.3 to 58.5 per 100000 persons. Significant regional and gender disparities were observed, with males experiencing a higher burden. Notably, China and India exhibited the most concerning epidemiological trends.

**CONCLUSIONS:**

The findings highlight the need for targeted public health interventions to address the rising burden of TREC, particularly in regions with high smoking rates. While population growth and aging are key contributors, improvements in public health policies have the potential to mitigate the TREC burden in certain areas. Further research is necessary to explore additional factors influencing TREC epidemiology and to quantify the observed regional and gender differences.

## INTRODUCTION

Esophageal cancer, as a fatal malignant tumor, is the sixth leading cause of cancer-related deaths^1^. According to the Global Burden of Disease (GBD) study, the incidence and mortality rates of esophageal cancer have been continuously rising worldwide^2^, with significant variations across different regions. These differences may be attributed to various factors, including genetic susceptibility, environmental influences, lifestyle choices, and dietary habits^3,4^. Analyzing the impact of specific factors on esophageal cancer can guide the formulation of targeted prevention policies and improve regional esophageal cancer control efforts.

Esophageal cancer is primarily classified into two histological types: squamous cell carcinoma (ESCC) and adenocarcinoma (EAC). Tobacco is a recognized risk factor for both types, although the mechanisms of carcinogenesis may differ^5^. Tobacco smoke contains various carcinogens, including nitrosamines and polycyclic aromatic hydrocarbons, which can induce DNA damage and promote tumor development^6^. Additionally, tobacco use is often associated with other risk factors, such as alcohol consumption and poor dietary habits, which can synergistically increase the risk of esophageal cancer^7^. Research indicates that tobacco not only directly affects the health of smokers but also increases the risk of esophageal cancer in non-smokers through secondhand smoke exposure^8^. Previous studies have explored the correlation between exposure to secondhand smoke and esophageal cancer, highlighting that exposure to secondhand smoke increases the risk of esophageal squamous cell carcinoma in non-smokers^9,10^.

Global tobacco consumption remains high: recent estimates show adult smoking prevalence around 25% worldwide, with particularly elevated rates (>50% in men) in Eastern Europe, East Asia, and parts of South America^11^. This geographical heterogeneity in tobacco use parallels marked regional differences in TREC incidence and mortality, with the highest burdens observed in China, India and former Soviet Union states – regions where male smoking prevalence often exceeds 45%^12^.

Compared with other etiological subtypes of esophageal cancer – such as those driven by gastroesophageal reflux, dietary carcinogens or thermal injury – TREC accounts for an estimated 30%–50% of global esophageal cancer cases and is distinguished by well-characterized carcinogenic mechanisms and strong dose–response relationships with tobacco exposure^13^. The clear etiological link to specific tobacco-derived carcinogens and the proven impact of tobacco control measures make TREC one of the most preventable cancer subtypes, underscoring its critical importance in public health^14^. Previous studies have shown that the burden of esophageal cancer varies by gender, age, and region, with tobacco playing an important role^15^. For instance, the incidence rate among males is generally higher than that among females, which may be related to higher smoking rates and greater social acceptance of tobacco use among men^16^.

Although studies have identified the epidemiological profile of esophageal cancer, there is a lack of systematic studies exploring the interaction between tobacco and other risk factors. This study aims to analyze the epidemiological data of TREC globally, investigating the disease burden and its trends across different regions, genders, and age groups. We will utilize data from the GBD 2021 to assess the mortality rates, disability-adjusted life years (DALYs), and other relevant indicators for TREC. Additionally, we will conduct decomposition analysis to identify the main factors influencing changes in the TREC burden, including population growth, aging, and epidemiological changes.

## METHODS

### Study population and data collection

This is a secondary dataset analysis of the GBD 2021^2^. The GBD 2021 study conducted a thorough evaluation of health losses across 204 countries and territories, utilizing the most recent epidemiological data and enhanced standardized methodologies. In this study, we extracted mortality and other relevant measures of TREC from the GBD database for further analysis. Notably, tobacco-associated esophageal cancer (TREC) is defined as a case of esophageal cancer associated with tobacco smoke. We extracted data using the GBD Results Tool (http://ghdx.healthdata.org/gbd-results-tool) following standardized protocols. Our extraction encompassed all available countries and territories (n=204), covering the period from 1990 to 2021. We included data for all age groups provided in the GBD database (14 age groups ranging from 30–35 years to ≥95 years) and all genders. For inclusion/exclusion criteria, we selected all data points categorized under ‘esophageal cancer’ (International Classification of Diseases, 10th Revision [ICD-10] codes C15.0–C15.9) with the risk factor specified as ‘Tobacco’ (including smoking, chewing tobacco, and secondhand smoke). For comprehensive burden assessment, we analyzed four dimensions: mortality (deaths), morbidity (years lived with disability [YLDs]), premature mortality (years of life lost [YLLs]), and their composite measure (disability-adjusted life years [DALYs]). All extracted data underwent standardization according to GBD methodology, including age adjustment using the GBD world population age standard and calculation of age-standardized rates per 100000 person-years to enable valid cross-population comparisons.

The age-standardized rates (ASR) applied in the GBD study conformed to the criteria for the global population. To account for uncertainties in parameter predictions, model selection, and data compilation, the projected disease burden was presented as a 95% uncertainty interval (UI), reflecting a 95% probability of the true parameter values^17^. The sociodemographic index (SDI) is a composite measure based on income per capita, average education level, and fertility level. It ranges from 0 to 1 and categorizes countries into five categories: low, low-middle, middle, high-middle, and high SDI^18^.

### Statistical analysis

The ASR for TREC was calculated using the GBD Population Standard Framework with projections based on 1000 iterations and a 95% UI^17^.

We conducted a descriptive analysis to assess the burden of TREC at various levels by comparing the deaths, DALYs, years lived with disability (YLDs), and years of life lost (YLLs) across sex and age groups. Joinpoint regression was used to calculate the annual percentage change (APC) and average annual percentage change (AAPC) in smoking-attributable gastrointestinal (GI) cancer burden from 1990 to 2023. AAPC was calculated as:


$$\text{AAPC}=[\text{exp}(\frac{\text{Σ}{\text{w}}_{\text{i}}{\text{b}}_{\text{i}}}{\text{Σ}{\text{w}}_{\text{i}}})-1]\times 100$$

where w_i_ is segment length and b_i_ is the slope coefficient. Trends were considered increasing if the AAPC and its 95% confidence interval (CI) exceeded zero, decreasing if both were below zero, and stable otherwise^19^.

In addition, we conducted a decomposition analysis to gain a deeper understanding of the factors influencing the TREC burden from 1990 to 2021^20^. We first identified three key driving factors: population growth, aging, and epidemiological changes, and constructed the following mathematical model: the total burden of TREC equals the sum of contributions from each factor^21^. Subsequently, we utilized R statistical software to perform numerical calculations on the data, specifically by calculating the contribution rate of each factor to the changes in TREC across different years, with the formula being the ratio of the change in TREC attributed to a specific factor to the total change in TREC.

All statistical analyses and data visualizations were conducted using R programming software (version 4.2.3). The *dplyr* package (version 1.1.3) and the *data.table* package (version 1.14.8) were utilized for data manipulation. For creating plots, the *ggplot2* package (version 3.5.1) was employed.

## RESULTS

### Deaths

**Figure 1:**
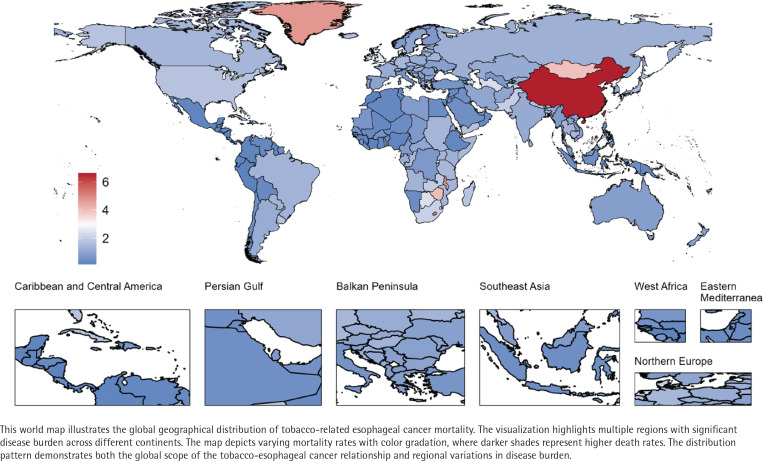
Global geographical distribution of tobacco-related esophageal cancer

From 1990 to 2021, the number of deaths due to TREC increased from 143332.8 (95% UI: 117012.5–170295) to 219185.3 (95% UI: 172166.4–270731.2), while the age-standardized death rate (ASDR) decreased from 3.6 per 100000 persons to 2.5 per 100000 persons (Table 1 and Figure 1; and Supplementary file Table 1 and Figure 1). Decomposition analysis revealed that the rise in TREC deaths was primarily attributed to population growth (154.62% relative contribution) and aging (39.75% relative contribution), while epidemiological changes (-94.37% relative contribution) had a suppressive effect (Table 2; and Supplementary file Figure 2). This paradoxical pattern – rising absolute mortality alongside declining age-standardized rates – predominantly reflects demographic transitions. Decomposition analysis quantified these drivers: population growth accounted for 154.62% of the mortality increase (equivalent to 338137 excess deaths), population aging contributed 39.75% (86915 deaths), while improved age-specific risk (epidemiological changes) averted 94.37% of potential deaths (-206412 deaths) (Table 2; and Supplementary file Figure 2). Notably, aging populations have become the dominant force – in high-SDI regions, aging alone explained 218% of mortality increases from 2010–2021.

**Table 1:** Number of deaths and disease-burden metrics by year and sex (point estimates)

	*1990 Deaths*		*2021 Deaths*		*1990 DALYs*		*2021 DALYs*		*1990 YLDs*		*2021 YLDs*		*1990 YLLs*		*2021 YLLs*	
	*Number* *(95% UI)*	*ASR/100000 persons* *(95% UI)*	*Number* *(95% UI)*	*ASR/100000 persons* *(95% UI)*	*Number* *(95% UI)*	*ASR/100000 persons* *(95% UI)*	*Number* *(95% UI)*	*ASR/100000 persons* *(95% UI)*	*Number* *(95% UI)*	*ASR/100000 persons* *(95% UI)*	*Number* *(95% UI)*	*ASR/100000 persons* *(95% UI)*	*Number* *(95% UI)*	*ASR/100000 persons* *(95% UI)*	*Number* *(95% UI)*	*ASR/100000 persons* *(95% UI)*
**Global**	143332.8 (117012.5–170295)	3.6 (3–4.3)	219185.3 (172166.4–270731.2)	2.5 (2–3.1)	3844095.6 (3139093.9–4585376.4)	93.3 (76.2–111.3)	5136277 (4040644.3–6350151.2)	58.5 (46–72.3)	37913.2 (26500.5–51322.3)	0.9 (0.7–1.3)	63202.3 (42826.7–87266.3)	0.7 (0.5–1)	3806182.4 (3105052.1–4543522.9)	92.4 (75.3–110.3)	5073074.8 (3987558.6–6269501.3)	57.8 (45.4–71.4)
**Sex**																
**Male**	128460.4 (104173.7–153779.4)	7.1 (5.8–8.5)	201411.2 (157094.9–250086.6)	5.1 (4–6.3)	3500527.9 (2831848.8–4207047)	179.7 (145.8–215.2)	4754997.4 (3709201.3–5904764.1)	114.8 (89.5–142.6)	33972.2 (23632–46126.9)	1.8 (1.3–2.4)	57905.6 (39226.3–80320.3)	1.4 (1–2)	3466555.6 (2801253.1–4165039.6)	177.9 (144.1–213.1)	4697091.8 (3660823.5–5826298)	113.4 (88.4–140.7)
**Female**	14872.4 (11144.9–18530.2)	0.7 (0.5–0.9)	17774.1 (13596.8–22344.1)	0.4 (0.3–0.5)	343567.7 (259972.5–426105.1)	16.1 (12.2–20)	381279.6 (292975–487652.5)	8.3 (6.3–10.6)	3940.9 (2629–5563.8)	0.2 (0.1–0.3)	5296.7 (3524.2–7611.1)	0.1 (0.1–0.2)	339626.8 (256675.6–421167.8)	16 (12.1–19.8)	375983 (288756.4–481016.4)	8.1 (6.3–10.4)
**SDI**																
**High**	27941.6 (22890.9–32511.2)	2.5 (2.1–2.9)	36045.4 (28280.5–43800.9)	1.7 (1.3–2)	676154.4 (558730.9–787082.5)	62.7 (51.9–73)	763421.5 (608706.6–920289.8)	38.5 (30.9–46.3)	8505.8 (6027.1–11378.1)	0.8 (0.6–1)	12728 (8788.3–17509.8)	0.6 (0.4–0.9)	667648.6 (551973.7–774890)	62 (51.3–71.9)	750693.5 (597699.4–905917.1)	37.9 (30.4–45.6)
**High-middle**	46617.9 (37143.7–56995.5)	4.7 (3.7–5.7)	75223.5 (56618.2–98193.9)	3.7 (2.8–4.9)	1262909.4 (1002474.6–1542666.1)	122.6 (97.5–149.8)	1763633.7 (1324867.1–2308908.3)	87.6 (65.8–114.5)	11901.8 (8016.8–16326)	1.2 (0.8–1.6)	21524.6 (14106.3–31099.1)	1.1 (0.7–1.5)	1251007.6 (991527.6–1527908.9)	121.4 (96.5–148.3)	1742109.1 (1309166.9–2276815.9)	86.5 (65–113)
**Middle**	56459.5 (44978–69933.3)	5.5 (4.4–6.8)	86733.1 (65047.8–112366.3)	3.3 (2.5–4.3)	1556511.5 (1242518–1932994.1)	140.7 (112.5–174.5)	2037140.5 (1540952.1–2630987.7)	73.4 (55.4–94.9)	14279.9 (9611–19996)	1.3 (0.9–1.9)	23381.7 (15228.1–32589.6)	0.9 (0.6–1.2)	1542231.6 (1228770.3–1916601)	139.4 (111.4–172.9)	2013758.8 (1521211.6–2603570.6)	72.6 (54.7–93.9)
**Low-middle**	9478.5 (7884.3–11286.1)	1.6 (1.3–1.9)	16581.5 (13541.9–19828.7)	1.2 (1–1.4)	267895.1 (224058.1–319292.7)	40.9 (34–48.7)	443711 (362461.1–531665.3)	29.4 (24–35.1)	2492.3 (1710.7–3329.8)	0.4 (0.3–0.5)	4367 (2990.6–6078.5)	0.3 (0.2–0.4)	265402.8 (221971.9–316446)	40.5 (33.7–48.2)	439344 (359081.8–525897.2)	29.1 (23.8–34.7)
**Low**	2773.9 (2252.8–3320)	1.3 (1–1.5)	4524 (3601.5–5573.8)	0.9 (0.7–1.1)	79010.8 (64043.5–94590.9)	32.4 (26.2–38.7)	126444.4 (100439.6–155506.9)	23.2 (18.5–28.5)	717.1 (503.1–984.7)	0.3 (0.2–0.4)	1179.3 (797.2–1650.8)	0.2 (0.2–0.3)	78293.7 (63489.4–93683.2)	32.1 (26–38.3)	125265 (99500.5–154293.8)	23 (18.3–28.3)

ASR: age-standardized rate. DALYs: disability-adjusted life years. YLLs: years of life lost. YLDs: years lived with disability. UI: uncertainty interval.

**Table 2:** Changes in the burden of tobacco-related esophageal cancer due to the factors of aging, population, and epidemiological change from 1990 to 2021

	*Deaths*				*DALYs*				*YLDs*				*YLLs*			
	*Overall difference*	*Aging (%)*	*Population (%)*	*Epidemiological change (%)*	*Overall difference*	*Aging (%)*	*Population (%)*	*Epidemiological change (%)*	*Overall difference*	*Aging (%)*	*Population (%)*	*Epidemiological change (%)*	*Overall difference*	*Aging (%)*	*Population (%)*	*Epidemiological change (%)*
**Global**	75852.52	30150.21 (39.75)	117281.46 (154.62)	-71579.15 (-94.37)	1292181.47	595751.43 (46.1)	2970342.42 (229.87)	-2273912.37 (-175.97)	25289.11	7290.03 (28.83)	32169.63 (127.21)	-14170.54 (-56.03)	1266892.36	588461.4 (46.45)	2938172.79 (231.92)	-2259741.82 (-178.37)
**SDI**																
**High**	8103.84	7585.31 (93.6)	12895.64 (159.13)	-12377.11 (-152.73)	87267.16	133839.33 (153.37)	293907.2 (336.79)	-340479.37 (-390.16)	4222.26	2135.81 (50.58)	4185.67 (99.13)	-2099.22 (-49.72)	83044.9	131703.52 (158.59)	289721.53 (348.87)	-338380.15 (-407.47)
**High-middle**	28605.55	11975.54 (41.86)	30897.82 (108.01)	-14267.81 (-49.88)	500724.3	238296.03 (47.59)	783103.06 (156.39)	-520674.79 (-103.98)	9622.79	2813.7 (29.24)	8321.97 (86.48)	-1512.88 (-15.72)	491101.51	235482.33 (47.95)	774781.08 (157.76)	-519161.91 (-105.71)
**Middle**	30273.66	19035.39 (62.88)	55750.3 (184.15)	-44512.03 (-147.03)	480629	396122.52 (82.42)	1441817.37 (299.99)	-1357310.9 (-282.4)	9101.72	4378.45 (48.11)	14417.71 (158.41)	-9694.44 (-106.51)	471527.28	391744.08 (83.08)	1427399.66 (302.72)	-1347616.46 (-285.8)
**Low-middle**	7102.96	1347.11 (18.97)	10111.42 (142.35)	-4355.56 (-61.32)	175815.99	25465 (14.48)	278721.91 (158.53)	-128370.93 (-73.01)	1874.73	292.89 (15.62)	2658.04 (141.78)	-1076.2 (-57.41)	173941.25	25172.11 (14.47)	276063.87 (158.71)	-127294.73 (-73.18)
**Low**	1750.06	-202.15 (-11.55)	3236.77 (184.95)	-1284.56 (-73.4)	47433.56	-5960.03 (-12.56)	91501.28 (192.9)	-38107.69 (-80.34)	462.23	-55.56 (-12.02)	839.73 (181.67)	-321.93 (-69.65)	46971.33	-5904.46 (-12.57)	90661.55 (193.01)	-37785.76 (-80.44)

Overall difference = value (2021) - value (1990). DALYs: disability-adjusted life years. YLLs: years of life lost. YLDs: years lived with disability. SDI: sociodemographic index.

**Table 3:** Trends in age-standardized rate of tobacco-related esophageal cancer health metrics by sociodemographic index (SDI), 1990–2021

*SDI*	*ASR deaths/100000 persons*			*ASR DALYs/100000 persons*			*ASR YLDs/100000 persons*			*ASR YLLs/100000 persons*		
	*1990* *(95% UI)*	*2021* *(95% UI)*	*AAPC* *(95% CI)*	*1990* *(95% UI)*	*2021* *(95% UI)*	*AAPC* *(95% CI)*	*1990* *(95% UI)*	*2021* *(95% UI)*	*AAPC* *(95% CI)*	*1990* *(95% UI)*	*2021* *(95% UI)*	*AAPC* *(95% CI)*
High	2.5 (2.1–2.9)	1.7 (1.3–2)	-1.35 (-1.38 – -1.31)	62.7 (51.9–73)	38.5 (30.9–46.3)	-1.61 (-1.65 – -1.57)	0.8 (0.6–1)	0.6 (0.4–0.9)	-0.77 (-0.81 – -0.74)	62 (51.3–71.9)	37.9 (30.4–45.6)	-1.62 (-1.66 – -1.58)
High-middle	4.7 (3.7–5.7)	3.7 (2.8–4.9)	-0.75 (-0.82 – -0.68)	122.6 (97.5–149.8)	87.6 (-1.18 – -1.05)	-1.11 (65.8–114.5)	1.2 (0.8–1.6)	1.1 (0.7–1.5)	-0.3 (-0.37 – -0.24)	121.4 (96.5–148.3)	86.5 (65–113)	-1.12 (-1.19 – -1.06)
Middle	5.5 (4.4–6.8)	3.3 (2.5–4.3)	-1.66 (-1.75 – -1.57)	140.7 (112.5–174.5)	73.4 (55.4–94.9)	-2.08 (-2.16 – -2.01)	1.3 (0.9–1.9)	0.9 (0.6–1.2)	-1.45 (-1.53 – -1.37)	139.4 (111.4–172.9)	72.6 (54.7–93.9)	-2.09 (-2.16 – -2.02)
Low-middle	1.6 (1.3–1.9)	1.2 (1–1.4)	-0.92 (-0.96 – -0.88)	40.9 (34–48.7)	29.4 (24–35.1)	-1.02 (-1.05 – -0.99)	0.4 (0.3–0.5)	0.3 (0.2–0.4)	-0.88 (-0.91 – -0.85)	40.5 (33.7–48.2)	29.1 (23.8–34.7)	-1.02 (-1.05 – -0.99)
Low	1.3 (1–1.5)	0.9 (0.7–1.1)	-0.95 (-0.99 – -0.92)	32.4 (26.2–38.7)	23.2 (18.5–28.5)	-1.07 (-1.09 – -1.05)	0.3 (0.2–0.4)	0.2 (0.2–0.3)	-0.96 (-0.98 – -0.94)	32.1 (26–38.3)	23 (18.3–28.3)	-1.07 (-1.09 – -1.05)

ASR: age-standardized rate. DALYs: disability-adjusted life years. YLLs: years of life lost. YLDs: years lived with disability. AAPC: average annual percent change. CI: confidence interval. UI: uncertainty interval.

Joinpoint regression analysis indicated (Supplementary file Figure 3) that although the overall ASDR showed a declining trend (AAPC= -1.19), the rate of decline has gradually slowed (APC_2015-2021_= -0.51). In 2021, the ASDR for males (5.1 per 100000 persons) was significantly higher than that for females (0.4 per 100000 persons).

By SDI region, the ASDR for TREC showed a declining trend across all regions (AAPC<0), with the middle SDI regions experiencing the largest decrease (AAPC= -1.87) (Supplementary file: Figure 4 and Table 3). In 2021, the middle SDI regions had the highest number of TREC deaths (86733.1) and ASDR (3.3 per 100000 persons). However, correlation analysis did not provide evidence of an association between ASDR and SDI (p=0.152) (Supplementary file Figure 5).

At the national level, the three countries with the highest number of deaths in 2021 were China (140513.7), the USA (10453.4), and India (13222.7), while the countries with the highest ASDR were China (6.6 per 100000 persons), Lesotho (5.1 per 100000 persons), and Greenland (4.7 per 100000 persons).

### DALYs

**Figure 2:**
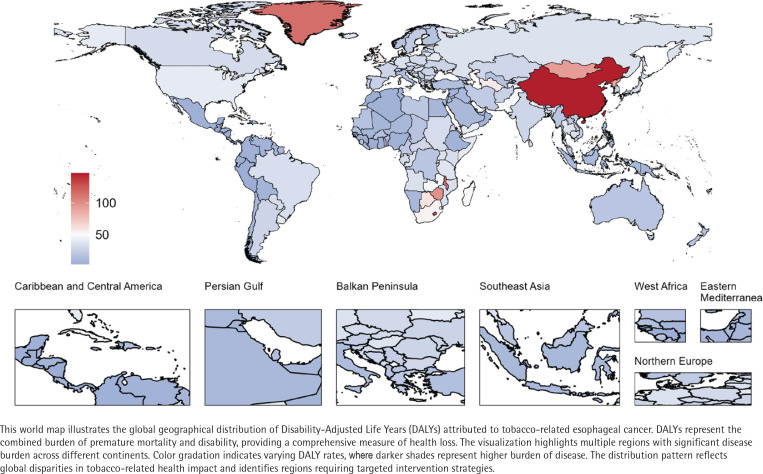
Global geographical distribution of tobacco-related esophageal cancer

From 1990 to 2021, the global DALYs due to TREC increased from 3844095.6 (95% UI: 3139093.9–4585376.4) to 5136277 (95% UI: 4040644.3–6350151.2), while the age-standardized DALYs rate (ASDR) decreased from 93.3 per 100000 persons to 58.5 per 100000 persons (Table 1 and Figure 2; and Supplementary file Table 2 and Figure 1). Decomposition analysis indicated that the rise in TREC-related DALYs was primarily attributed to population growth (229.87% relative contribution) and aging (46.1% relative contribution), while epidemiological changes had a suppressive effect (-175.97% relative contribution) (Table 2; and Supplementary file Figure 6). Joinpoint regression analysis results (Supplementary file Figure 7) showed that although the overall ASDR exhibited a declining trend (AAPC= -1.51), the rate of decline slowed during the period from 2016 to 2021 compared to earlier years (APC_2016-2021_= -0.64). By 2021, the ASDR for males (114.8 per 100000 persons) was significantly higher than that for females (8.3 per 100000 persons).

By SDI region, the ASDR for TREC showed a declining trend across all regions (AAPC<0), with the middle SDI regions experiencing the largest decrease (AAPC= -2.08) (Supplementary file: Figure 8 and Table 3). In 2021, the middle SDI region had the highest TREC-related DALYs, reaching 2037140.5, while the high SDI region had the highest ASDR at 87.6 per 100000 persons. However, correlation analysis did not reveal a significant association between ASDR and SDI (p=0.431) (Supplementary file Figure 9).

At the national level, the three countries with the highest TREC-related DALYs in 2021 were China (3238100.2), India (355612.7), and the USA (231871.1), while the countries with the highest ASDR were China (147.1 per 100000 persons), Lesotho (131.6 per 100000 persons), and Greenland (114.6 per 100000 persons).

### YLDs

**Figure 3:**
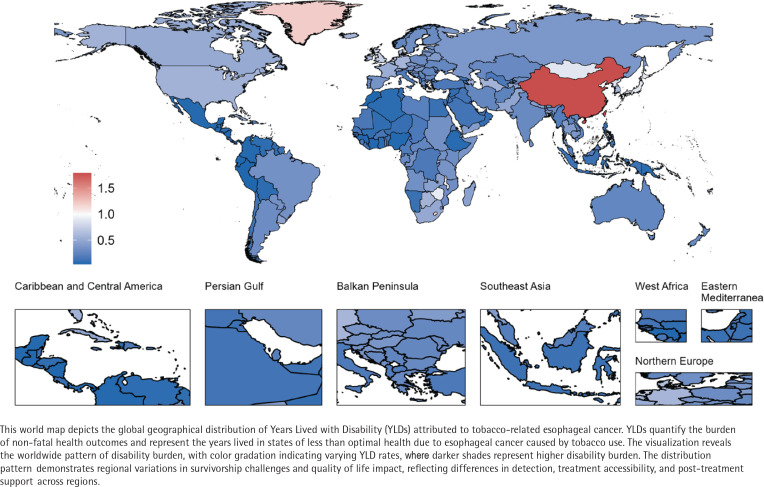
Global geographical distribution of tobacco-related esophageal cancer

From 1990 to 2021, the global years YLDs due to TREC increased from 37913.2 (95% UI: 26500.5–51322.3) to 63202.3 (95% UI: 42826.7–87266.3), while the age-standardized YLDs rate (ASYR) decreased from 0.9 per 100000 persons to 0.7 per 100000 persons (Table 1 and Figure 3; and Supplementary file Table 3 and Figure 1). Decomposition analysis indicated that the rise in TREC-related YLDs was primarily attributed to population growth (127.21% relative contribution) and aging (28.83% relative contribution), while epidemiological changes had a suppressive effect (-56.03% relative contribution) (Table 2; and Supplementary file Figure 10). Joinpoint regression analysis results (Supplementary file Figure 11) showed that although the overall ASYR exhibited a declining trend (AAPC= -0.85), the rate of decline has gradually slowed (APC_2016-2021_= -0.15). By 2021, the ASYR for males (1.4 per 100000 persons) was significantly higher than that for females (0.1 per 100000 persons).

By SDI region, the ASYR for TREC showed a declining trend across all regions (AAPC<0), with the middle SDI regions experiencing the largest decrease (AAPC= -1.45) (Supplementary file: Figure 12 and Table 3). In 2021, the middle SDI regions had the highest TREC-related YLDs, reaching 23381.7, while the high SDI region had the highest ASYR at 1.1 per 100000 persons. Correlation analysis indicated a positive relationship between ASYR and SDI (ρ=0.22, p<0.001) (Supplementary file Figure 13).

At the national level, China had significantly higher TREC-related YLDs in 2021 at 38990.7, followed by India (355612.7) and the USA (231871.1). The three countries with the highest ASYR in 2021 were China (1.8 per 100000 persons), Taiwan (1.2 per 100000 persons), and Lesotho (1.2 per 100000 persons).

### YLLs

**Figure 4:**
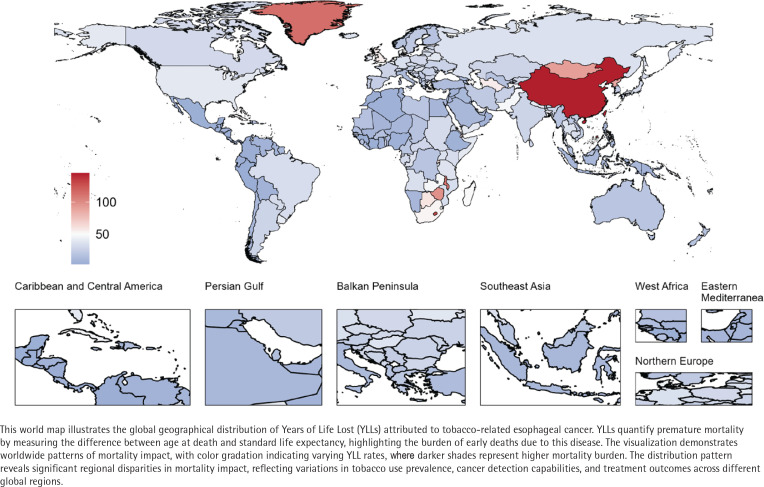
Global geographical distribution of tobacco-related esophageal cancer

From 1990 to 2021, the global YLLs due to TREC increased from 3806182.4 (95% UI: 3105052.1–4543522.9) to 5073074.8 (95% UI: 3987558.6–6269501.3), while the age-standardized YLLs rate (ASYLR) decreased from 92.4 per 100000 persons to 57.8 per 100000 persons (Table 1 and Figure 4; and Supplementary file Table 4 and Figure 1). Decomposition analysis indicated that the increase in TREC-related YLLs was primarily attributed to population growth (231.92% relative contribution) and aging (46.45% relative contribution), while epidemiological changes had a suppressive effect (-178.37% relative contribution) (Table 2; and Supplementary file Figure 14). Joinpoint regression analysis results (Supplementary file Figure 15) showed that although the overall ASYLR exhibited a declining trend (AAPC= -1.52), the rate of decline slowed during the period from 2016 to 2021 compared to earlier years (APC_2016-2021_= -0.65). By 2021, the ASYLR for males (113.4 per 100000 persons) was significantly higher than that for females (8.1 per 100000 persons).

By SDI region, the ASYLR for TREC showed a declining trend across all regions (AAPC<0), with the middle SDI regions experiencing the largest decrease (AAPC= -2.32) (Supplementary file: Figure 16 and Table 3). In 2021, the middle SDI regions had the highest TREC-related YLLs, reaching 2013758.8, while the High SDI region had the highest ASYLR at 86.5 per 100000 persons. However, correlation analysis did not reveal a significant association between ASYLR and SDI (p=0.446) (Supplementary file Figure 17).

At the national level, China had significantly higher TREC-related YLLs in 2021 at 3199109.5, followed by India (352096.9) and the USA (228599.6). The three countries with the highest ASYLR in 2021 were China (145.3 per 100000 persons), Lesotho (130.5 per 100000 persons), and Greenland (113.4 per 100000 persons).

## DISCUSSION

This study aims to analyze the epidemiological characteristics of TREC globally, exploring the disease burden and its trends across different regions, genders, and age groups. By utilizing the GBD 2021 data, we found that from 1990 to 2021, the number of deaths due to TREC significantly increased, particularly among males. This finding is consistent with previous research, which indicates that smoking rates are generally higher among men, and societal acceptance of smoking among men is greater, potentially leading to a higher incidence of TREC in this population^5^. Furthermore, the study identifies population growth and aging as the primary factors contributing to the increasing burden of TREC, this suggests that we need to implement specific policy measures to address these trends, such as enhancing health education and regular screenings for high-risk populations to reduce the future incidence of TREC^22^.

In the analysis of mortality rates and DALYs, we observed significant differences in the burden of TREC across different regions. The middle SDI regions exhibited the most substantial decline in ASDR, this indicates that public health interventions in these regions, such as smoking bans and health promotion campaigns, may have played an important role^23^. For high-burden regions, particularly in countries like China and India where we observed the highest absolute TREC burden, tailored public health interventions are urgently needed. These should include integrated approaches combining tobacco control with early detection screening, community-based education addressing both tobacco use and regional dietary habits, targeted screening policies for high-risk populations (especially male smokers aged >40 years), and culturally appropriate interventions for regions with disproportionately high age-standardized rates^24^. The differential resource allocation based on burden distribution is essential – in middle SDI regions with high absolute burden yet significant improvement potential, strengthening tobacco control while expanding early detection infrastructure could yield substantial benefits through collaboration between governmental agencies, healthcare institutions, and community organizations^25^.

To further strengthen the causal inference between tobacco use and TREC (trachea, esophagus, and respiratory cancers) burden, we analyzed smoking rate data from the World Health Organization’s Global Health Observatory, which revealed a significant positive correlation between national smoking rates and age-standardized DALYs (disability-adjusted life years) for TREC (r=0.68, p<0.001). This correlation was particularly pronounced in high-burden countries (China: smoking rate of 26.6%, TREC age-standardized death rate (ASDR) of 11.2/100000; India: smoking rate of 10.7%, TREC ASDR of 5.9/100000), and the association was stronger in men (r=0.72) than in women (r=0.51), consistent with the gender disparities we observed in TREC burden. Notably, this association was also confirmed in the Middle East, particularly in Iran, where studies from 1995–2015 showed distinct geographical and gender differences in esophageal cancer burden, especially in high-incidence northeastern areas, linked to specific tobacco use patterns in the region^26^. The situation in Iran is particularly complex due to the widespread socio-cultural acceptance of traditional waterpipe smoking. Despite the country implementing MPOWER measures based on the WHO Framework Convention on Tobacco Control, socially accepted waterpipe use, especially among young people, continues to impact the country’s TREC burden. Educational intervention studies based on the BASNEF model suggest that prevention strategies tailored to specific cultural contexts have the potential to reduce waterpipe use, which could be an important approach to controlling TREC burden in the Middle East^26^. Time-trend analysis indicates that countries with declining smoking rates (e.g. Australia, Canada) have experienced more significant reductions in TREC burden compared to regions with persistently high smoking rates (e.g. Eastern European men, smoking rate >30%).

Beyond smoking rates, various factors may explain regional disparities in the burden of esophageal cancer (TREC). Dietary habits are a significant influence; the prevalence of high-temperature food and beverages (e.g. hot tea) and the consumption of pickled foods in East and Central Asia are associated with increased risk of esophageal epithelial damage and carcinogenesis^27^. Differences in healthcare accessibility also significantly impact TREC prognosis. High SDI (sociodemographic index) regions generally have more developed endoscopic screening programs and early diagnostic capabilities, while limited healthcare resources in low-to-middle SDI regions lead to a higher proportion of late-stage diagnoses^28^. Genetic susceptibility factors, such as the ALDH2 and ADH1B gene variants more common in East Asian populations, may increase the risk of esophageal cancer in smokers^29^. Furthermore, environmental pollutants may synergize with carcinogens in tobacco, exacerbating TREC risk in specific regions^30^.

However, despite the overall downward trend in ASDR, correlation analysis failed to reveal a significant association between ASDR and SDI. When evaluating the effectiveness of public health policies, it is crucial to consider other potential factors, such as the allocation of medical resources and the dissemination of health education^31^. To incorporate these factors into prevention strategies, it is recommended to develop comprehensive health intervention programs that ensure effective resource allocation and enhance community health education^32^.

In the context of global tobacco control measures, many countries have recently implemented strict tobacco control policies, including smoke-free laws in public places, restrictions on tobacco advertising, and health education campaigns^33^. These policies have contributed, to some extent, to reducing smoking rates, thereby lowering the incidence and mortality of TREC. Especially in middle- and high-income countries, improvements in public health policies may be an important factor in explaining the decline in ASDR^34^. Additionally, targeted interventions for specific populations, such as youth smoking prevention and smoking cessation support, can have a more direct impact on tobacco use, thereby influencing the burden of TREC^35^.

Additionally, the results showed that TREC-related YLLs and YLDs also displayed similar trends. Although the increase in YLLs and YLDs was primarily attributed to population growth and aging, the suppressive effect of epidemiological changes suggests that the burden of TREC may have been effectively controlled in certain regions due to improvements in public health policies^36^. This finding emphasizes the importance of targeted interventions against tobacco use in reducing cancer burden, particularly in countries and regions with high smoking rates^15^.

### Strengths and limitations

A strength of this study is its use of the latest GBD data, providing a comprehensive global perspective that reveals the epidemiological characteristics of TREC and its trends. Moreover, the study employed decomposition analysis to investigate the main factors influencing changes in the TREC burden, offering scientific evidence for public health policy formulation. However, this study has several limitations. First, although the GBD data cover a broad spectrum of countries and regions, biases in data collection and reporting may persist – particularly in low-income settings – potentially introducing systematic error into our estimates. Second, as a secondary data analysis, we rely on GBD-modeled data, which inherently lack the accuracy of primary cohort or registry sources. Third, our trend analysis used a joinpoint regression model, an approach highly sensitive to the number of joinpoints, smoothing parameters and underlying assumptions (e.g. linear segmented trends); varying these settings can essentially alter the estimated annual percent change (APC). Fourth, smoking exposure is self-reported and may be under- or mis-reported – especially across diverse sociocultural or regulatory environments – thereby affecting the precision of smoking-attributable burden estimates. Fifth, we did not conduct systematic sensitivity analyses of short-term or regional fluctuations in smoking prevalence, which may limit the granularity of policy-impact assessments. Finally, the ecological design of this study only permits population-level associations and cannot establish individual-level causality, raising the risk of ecological fallacy. Moreover, the GBD methodology does not fully capture complex social and environmental determinants – such as socio-economic status, cultural practices, and healthcare-system differences – that could affect the generalizability and policy relevance of our findings.

## CONCLUSIONS

This study analyzed the epidemiological characteristics of TREC globally and found that from 1990 to 2021, the disease burden due to TREC significantly increased, particularly among males. Decomposition analysis revealed that population growth and aging were driving factors behind the continued rise in the TREC burden. Furthermore, the TREC burden exhibited significant regional and gender disparities, which compels us to pay greater attention to the development of targeted public health intervention policies.

## Supplementary Material



## Data Availability

The data supporting this research are available from the Global Health Data Exchange GBD 2021 website (https://ghdx.healthdata.org/gbd2019/data-input-sources).

## References

[1] Yang H, Wang F, Hallemeier CL, Lerut T, Fu J. Oesophageal cancer. Lancet. 2024;404(10466):1991-2005. doi:10.1016/S0140-6736(24)02226-8 39550174

[2] GBD 2021 Diseases and Injuries Collaborators. Global incidence, prevalence, years lived with disability (YLDs), disability-adjusted life-years (DALYs), and healthy life expectancy (HALE) for 371 diseases and injuries in 204 countries and territories and 811 subnational locations, 1990-2021: a systematic analysis for the Global Burden of Disease Study 2021. Lancet. 2024;403(10440):2133-2161. doi:10.1016/S0140-6736(24)00757-8 38642570 PMC11122111

[3] Kamangar F, Chow WH, Abnet CC, Dawsey SM. Environmental causes of esophageal cancer. Gastroenterol Clin North Am. 2009;38(1):27-vii. doi:10.1016/j.gtc.2009.01.004 19327566 PMC2685172

[4] Wang AH, Sun CS, Li LS, Huang JY, Chen QS, Xu DZ. Genetic susceptibility and environmental factors of esophageal cancer in Xi’an. World J Gastroenterol. 2004;10(7):940-944. doi:10.3748/wjg.v10.i7.940 15052670 PMC4717108

[5] Cook MB, Kamangar F, Whiteman DC, et al. Cigarette smoking and adenocarcinomas of the esophagus and esophagogastric junction: a pooled analysis from the international BEACON consortium. J Natl Cancer Inst. 2010;102(17):1344-1353. doi:10.1093/jnci/djq289 20716718 PMC2935475

[6] Pfeifer GP, Denissenko MF, Olivier M, Tretyakova N, Hecht SS, Hainaut P. Tobacco smoke carcinogens, DNA damage and p53 mutations in smoking-associated cancers. Oncogene. 2002;21(48):7435-7451. doi:10.1038/sj.onc.1205803 12379884

[7] Fan Y, Yuan JM, Wang R, Gao YT, Yu MC. Alcohol, tobacco, and diet in relation to esophageal cancer: the Shanghai cohort study. Nutr Cancer. 2008;60(3):354-363. doi:10.1080/01635580701883011 18444169 PMC2409004

[8] Duan L, Wu AH, Sullivan-Halley J, Bernstein L. Passive smoking and risk of oesophageal and gastric adenocarcinomas. Br J Cancer. 2009;100(9):1483-1485. doi:10.1038/sj.bjc.6605023 19352383 PMC2694436

[9] Rafiq R, Shah IA, Bhat GA, et al. Secondhand smoking and the risk of esophageal squamous cell carcinoma in a high incidence region, Kashmir, India: a case-control-observational study. Medicine. 2016;95(1):e2340. doi:10.1097/MD.0000000000002340 26735535 PMC4706255

[10] Wang QL, Xie SH, Li WT, Lagergren J. Smoking cessation and risk of esophageal cancer by histological type: systematic review and meta-analysis. J Natl Cancer Inst. 2017;109(12):10.1093/jnci/djx115. doi:10.1093/jnci/djx115 29933436

[11] Siddiqi K, Husain S, Vidyasagaran A, Readshaw A, Mishu MP, Sheikh A. Global burden of disease due to smokeless tobacco consumption in adults: an updated analysis of data from 127 countries. BMC Med. 2020;18(1):222. doi:10.1186/s12916-020-01677-9 32782007 PMC7422596

[12] Qi L, Sun M, Liu W, et al. Global esophageal cancer epidemiology in 2022 and predictions for 2050: A comprehensive analysis and projections based on GLOBOCAN data. Chin Med J. 2024;137(24):3108-3116. doi:10.1097/CM9.0000000000003420 39668405 PMC11706580

[13] GBD 2017 Oesophageal Cancer Collaborators. The global, regional, and national burden of oesophageal cancer and its attributable risk factors in 195 countries and territories, 1990-2017: a systematic analysis for the Global Burden of Disease Study 2017. Lancet Gastroenterol Hepatol. 2020;5(6):582-597. doi:10.1016/S2468-1253(20)30007-8 32246941 PMC7232026

[14] Cai Y, Lin J, Wei W, Chen P, Yao K. Burden of esophageal cancer and its attributable risk factors in 204 countries and territories from 1990 to 2019. Front Public Health. 2022;10:952087. doi:10.3389/fpubh.2022.952087 36148334 PMC9485842

[15] Zhang HZ, Jin GF, Shen HB. Epidemiologic differences in esophageal cancer between Asian and Western populations. Chin J Cancer. 2012;31(6):281-286. doi:10.5732/cjc.011.10390 22507220 PMC3777490

[16] Liu CQ, Ma YL, Qin Q, et al. Epidemiology of esophageal cancer in 2020 and projections to 2030 and 2040. Thorac Cancer. 2023;14(1):3-11. doi:10.1111/1759-7714.14745 36482832 PMC9807450

[17] GBD 2019 Universal Health Coverage Collaborators. Measuring universal health coverage based on an index of effective coverage of health services in 204 countries and territories, 1990-2019: a systematic analysis for the Global Burden of Disease Study 2019. Lancet. 2020;396(10258):1250-1284. doi:10.1016/S0140-6736(20)30750-9 32861314 PMC7562819

[18] GBD 2019 Diseases and Injuries Collaborators. Global burden of 369 diseases and injuries in 204 countries and territories, 1990-2019: a systematic analysis for the Global Burden of Disease Study 2019. Lancet. 2020;396(10258):1204-1222. doi:10.1016/S0140-6736(20)30925-9 33069326 PMC7567026

[19] Rosenberg PS, Check DP, Anderson WF. A web tool for age-period-cohort analysis of cancer incidence and mortality rates. Cancer Epidemiol Biomarkers Prev. 2014;23(11):2296-2302. doi:10.1158/1055-9965.EPI-14-0300 25146089 PMC4221491

[20] Chevan A, Sutherland M. Revisiting Das Gupta: refinement and extension of standardization and decomposition. Demography. 2009;46(3):429-449. doi:10.1353/dem.0.0060 19771938 PMC2831344

[21] Das Gupta P. Standardization and decomposition of rates from cross-classified data. Genus. 1994;50(3-4):171-196. 12319256

[22] Yancik R. Cancer burden in the aged: an epidemiologic and demographic overview. Cancer. 1997;80(7):1273-1283. 9317180

[23] Jin W, Huang K, Ding Z, et al. Global, regional, and national burden of esophageal cancer: a systematic analysis of the Global Burden of Disease Study 2021. Biomark Res. 2025;13(1):3. doi:10.1186/s40364-024-00718-2 39762900 PMC11702276

[24] Jin ZY, Liu K, Wallar G, et al. Environmental tobacco smoking (ETS) and esophageal cancer: A population-based case-control study in Jiangsu Province, China. Int J Cancer. 2025;156(8):1552-1562. doi:10.1002/ijc.35254 39552259 PMC11826109

[25] Cofer J, Hurst AN, Winter T, et al. A comprehensive program to reduce tobacco-related cancers through actions by a national cancer institute-designated cancer center. Cancer Control. 2022;29:10732748221138713. doi:10.1177/10732748221138713 36373741 PMC9663624

[26] Rahmani H, Sarabi Asiabar A, Niakan S, et al. Burden of esophageal cancer in Iran during 1995-2015: Review of findings from the global burden of disease studies. Med J Islam Repub Iran. 2018;32:55. doi:10.14196/mjiri.32.55 30175081 PMC6113580

[27] Zhong Y, Yang C, Wang N, Pan D, Wang S, Sun G. Hot tea drinking and the risk of esophageal cancer: a systematic review and meta-analysis. Nutr Cancer. 2022;74(7):2384-2391. doi:10.1080/01635581.2021.2007963 34818954

[28] Qu HT, Li Q, Hao L, et al. Esophageal cancer screening, early detection and treatment: Current insights and future directions. World J Gastrointest Oncol. 2024;16(4):1180-1191. doi:10.4251/wjgo.v16.i4.1180 38660654 PMC11037049

[29] Choi CK, Yang J, Kweon SS, et al. Association between ALDH2 polymorphism and esophageal cancer risk in South Koreans: a case-control study. BMC Cancer. 2021;21(1):254. doi:10.1186/s12885-021-07993-4 33750341 PMC7941978

[30] Simba H, Kuivaniemi H, Abnet CC, Tromp G, Sewram V. Environmental and life-style risk factors for esophageal squamous cell carcinoma in Africa: a systematic review and meta-analysis. BMC Public Health. 2023;23(1):1782. doi:10.1186/s12889-023-16629-0 37710248 PMC10500769

[31] Ya-Qing L, Hao-Ran N, Xiang-Yang T, et al. Research on equity of medical resource allocation in Yangtze river economic belt under healthy China strategy. Front Public Health. 2023;11:1175276. doi:10.3389/fpubh.2023.1175276 37435525 PMC10332165

[32] Pons-Vigués M, Diez È, Morrison J, et al. Social and health policies or interventions to tackle health inequalities in European cities: a scoping review. BMC Public Health. 2014;14:198. doi:10.1186/1471-2458-14-198 24564851 PMC3938820

[33] Flor LS, Reitsma MB, Gupta V, Ng M, Gakidou E. The effects of tobacco control policies on global smoking prevalence. Nat Med. 2021;27(2):239-243. doi:10.1038/s41591-020-01210-8 33479500 PMC7884287

[34] Bialous SA, van der Eijk Y. How should global tobacco control efforts be prioritized to protect children in resource-poor regions? AMA J Ethics. 2020;22(2):E135-E146. doi:10.1001/amajethics.2020.135 32048584

[35] Momenabadi V, Hossein Kaveh PhD M, Hashemi SY, Borhaninejad VR. Factors affecting hookah smoking trend in the society: a review article. Addict Health. 2016;8(2):123-135. 27882210 PMC5115646

[36] Henley SJ, Thomas CC, Sharapova SR, et al. Vital signs: disparities in tobacco-related cancer incidence and mortality - United States, 2004-2013. MMWR Morb Mortal Wkly Rep. 2016;65(44):1212-1218. doi:10.15585/mmwr.mm6544a3 27832048

