# Supplementary material for: Regional and gender disparities in tobacco-related esophageal cancer: Insights from the Global Burden of Disease study 1990–2021
Source: Tob Induc Dis. 2025 Jul 19;23:10.18332/tid/205670. doi: 10.18332/tid/205670 (PMC12278272; doi:10.18332/tid/205670)
Supplement: Supplementary file 1 [file TID-23-96-s1.pdf]

SUPPLEMENTAL MATERIAL

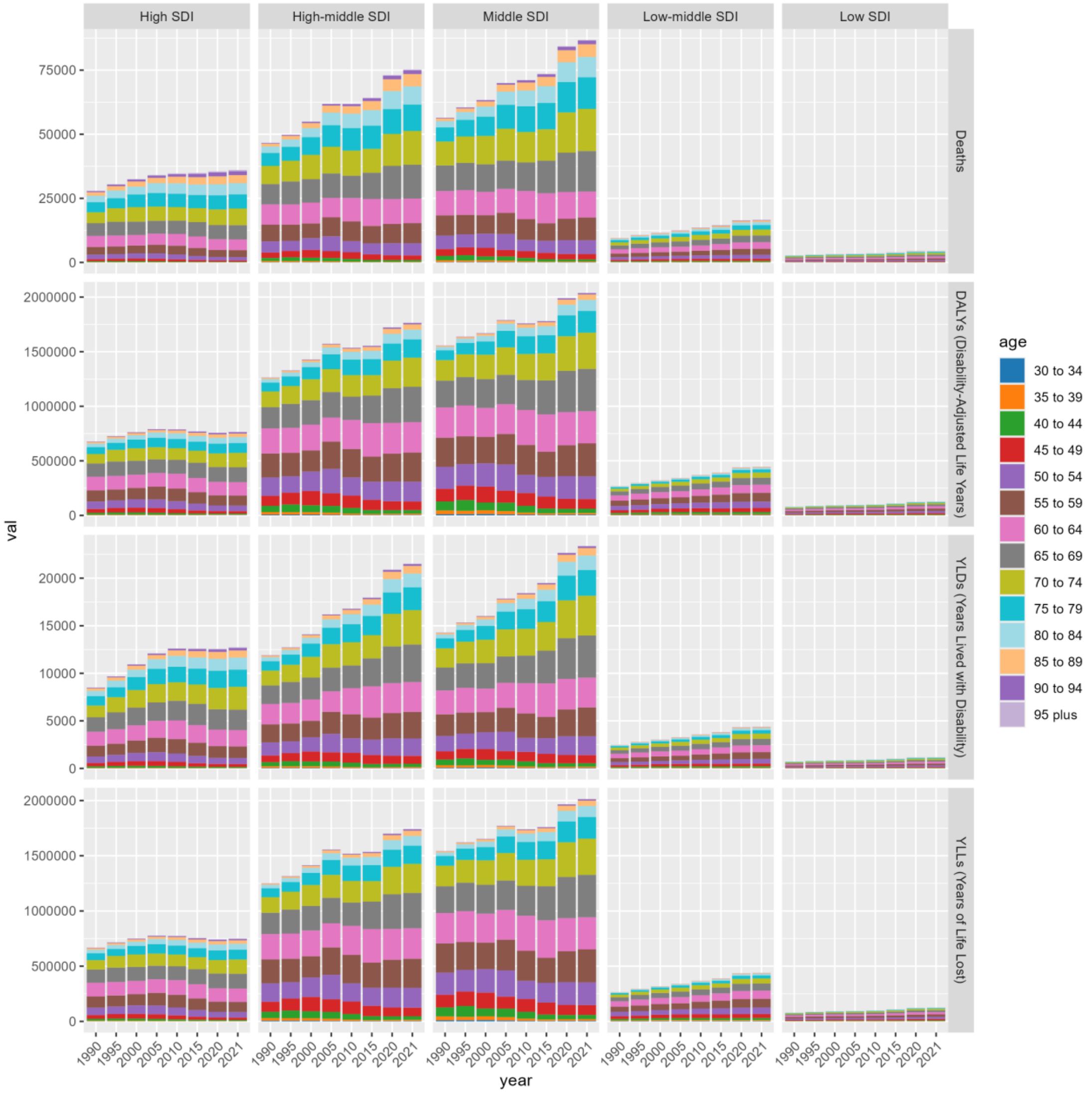

sFigure 1 Global Health Metrics Grid: ASR of Deaths, DALYs, YLDs, and YLLs across Sociodemographic Index (SDI) Levels for Tobacco-Related Esophageal Cancer.

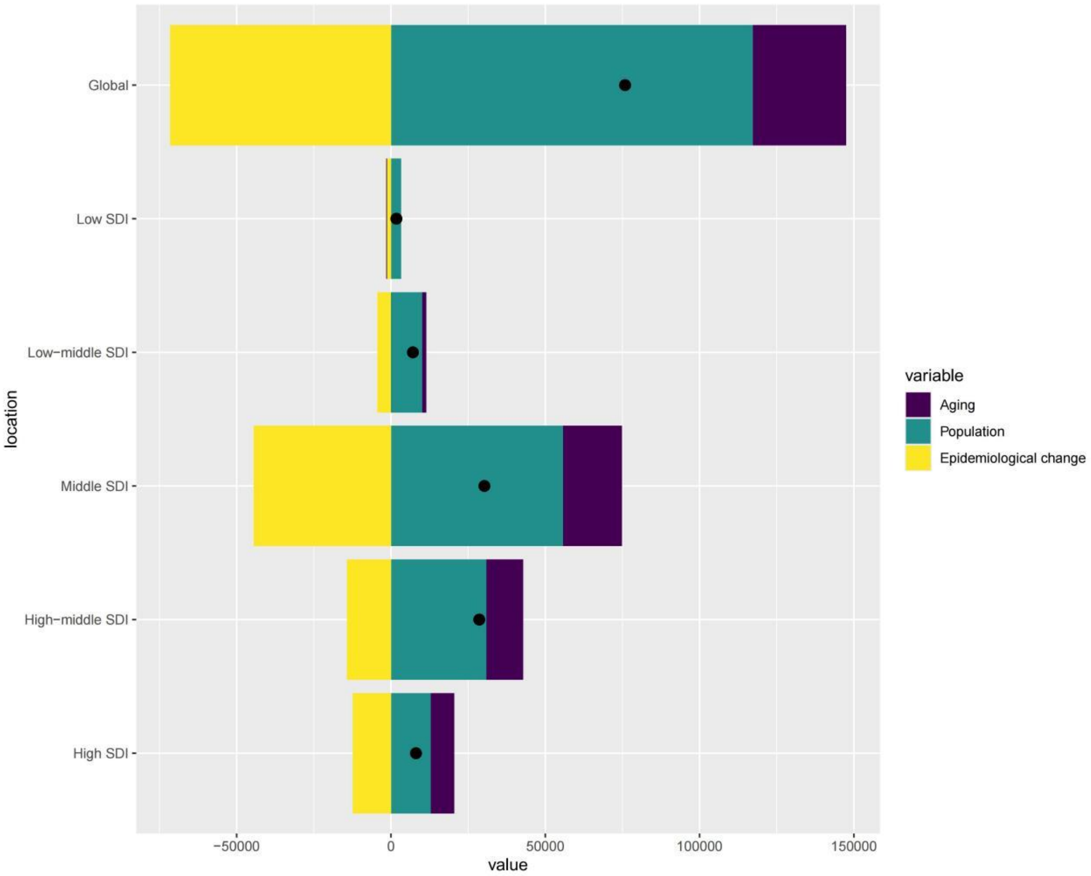

sFigure 2. Decomposition Analysis of Tobacco-Related Esophageal Cancer Mortality by Socio-demographic Index (SDI) Levels.

This figure presents a comprehensive decomposition analysis of tobacco-related esophageal cancer mortality across different Socio-demographic Index (SDI) levels. The visualization breaks down the mortality burden into three key contributing factors: population growth, population aging, and changes in age-specific rates. This three-factor decomposition framework reveals how demographic and epidemiological transitions influence tobacco-related esophageal cancer deaths across the socioeconomic spectrum.

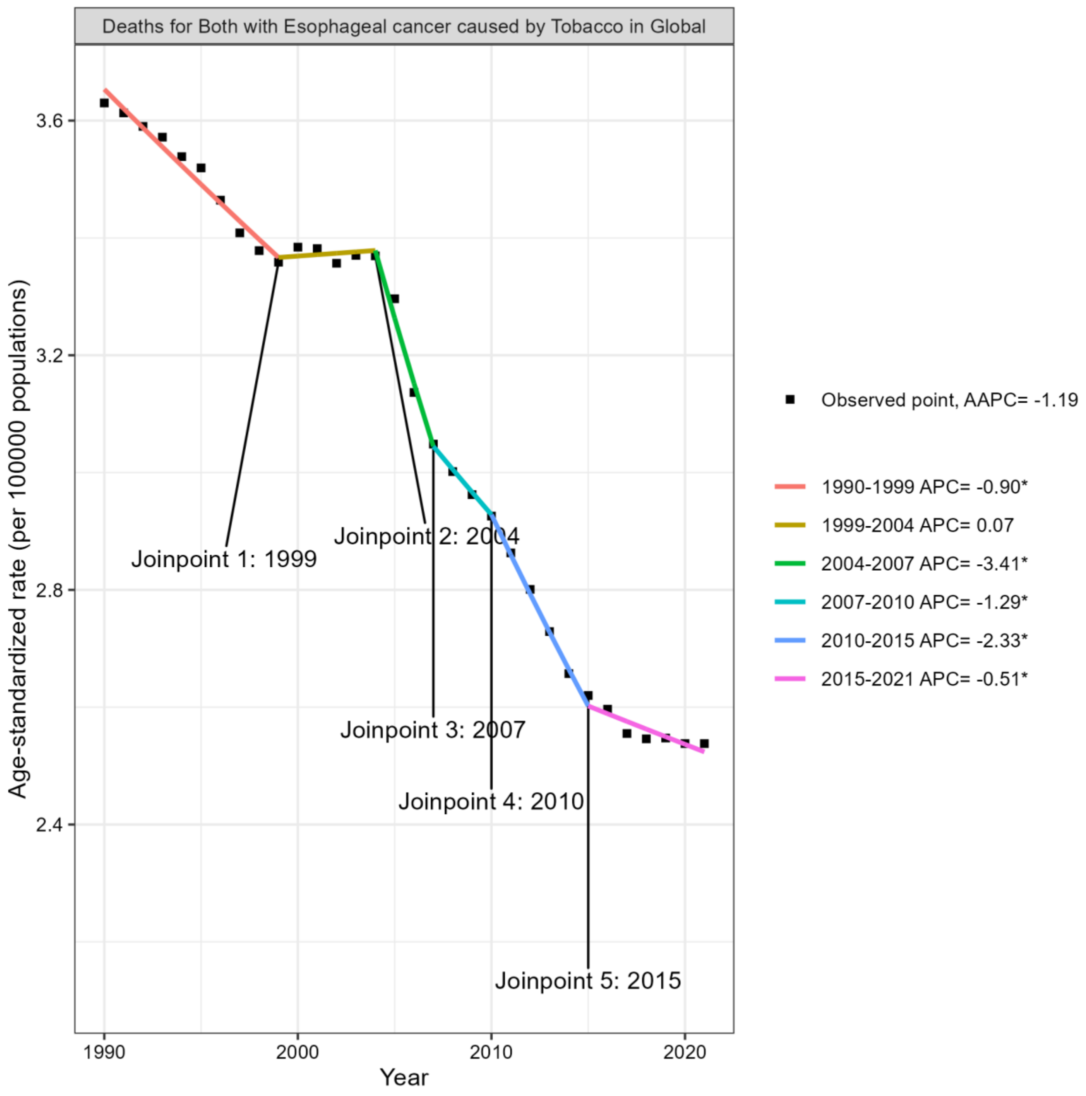

sFigure 3. Joinpoint Regression Analysis of Global Tobacco-Related Esophageal Cancer Mortality Trends: Identification of Significant Trend Changes in Death Rates (1990-2021). This figure illustrates the temporal trends in global tobacco-related esophageal cancer mortality from 1990 to 2021 using joinpoint regression analysis. The visualization depicts the annual percentage change (APC) in death rates over the 32-year period, with identified joinpoints marking statistically significant changes in mortality trends. Each segment between joinpoints represents a distinct period with a consistent rate of change.

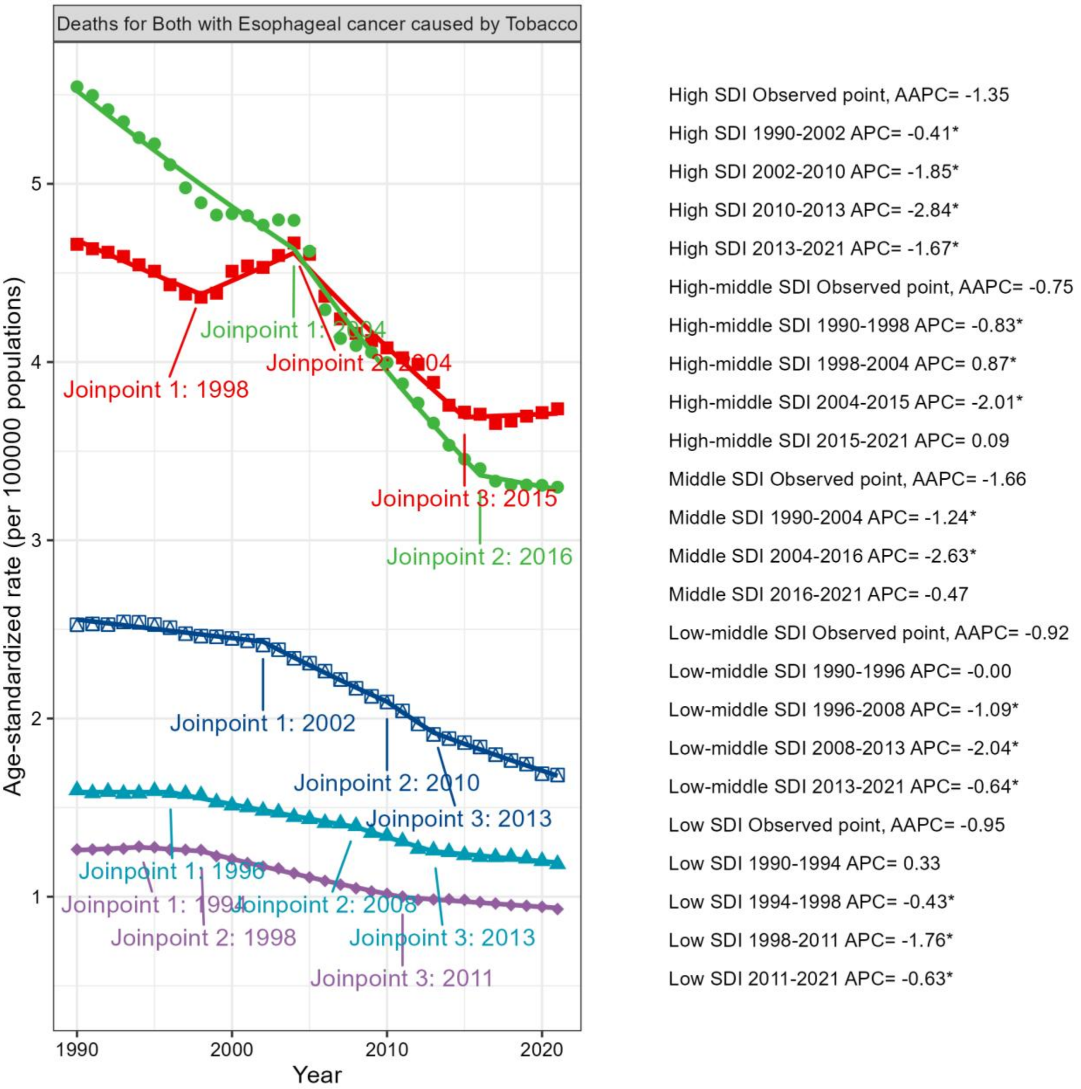

sFigure 4. Joinpoint Regression Analysis of Tobacco-Related Esophageal Cancer Mortality by Sociodemographic Index (SDI) Levels: Comparative Trend Analysis of Death Rates Across Socioeconomic Strata (1990-2021).

This figure presents a comparative joinpoint regression analysis of tobacco-related esophageal cancer mortality trends across different Sociodemographic Index (SDI) levels from 1990 to 2021. The visualization depicts distinct mortality trajectories for high, high-middle, middle, low-middle, and low SDI regions, with identified joinpoints marking statistically significant changes in death rate trends.

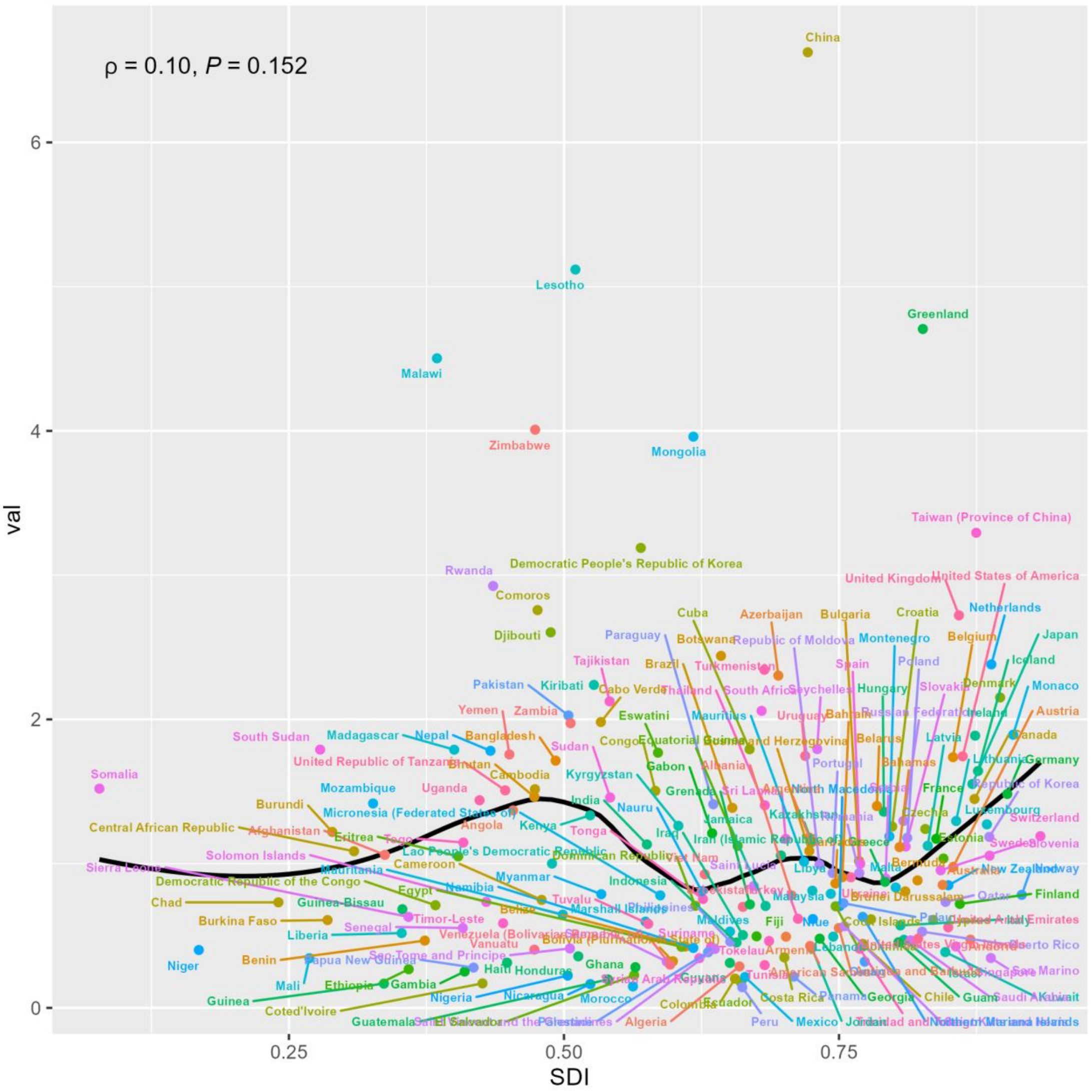

sFigure 5. Correlation between Sociodemographic Index (SDI) and Age-Standardized Death Rates (ASDR) for Tobacco-Related Esophageal Cancer Across Countries: Global Socioeconomic Disparities in Cancer Mortality.

This figure illustrates the relationship between national socioeconomic development status, as measured by the Sociodemographic Index (SDI), and the burden of tobacco-related esophageal cancer mortality, represented by Age-Standardized Death Rates (ASDR). Each data point represents an individual country, allowing for cross-national comparison of cancer mortality burden in relation to socioeconomic development.

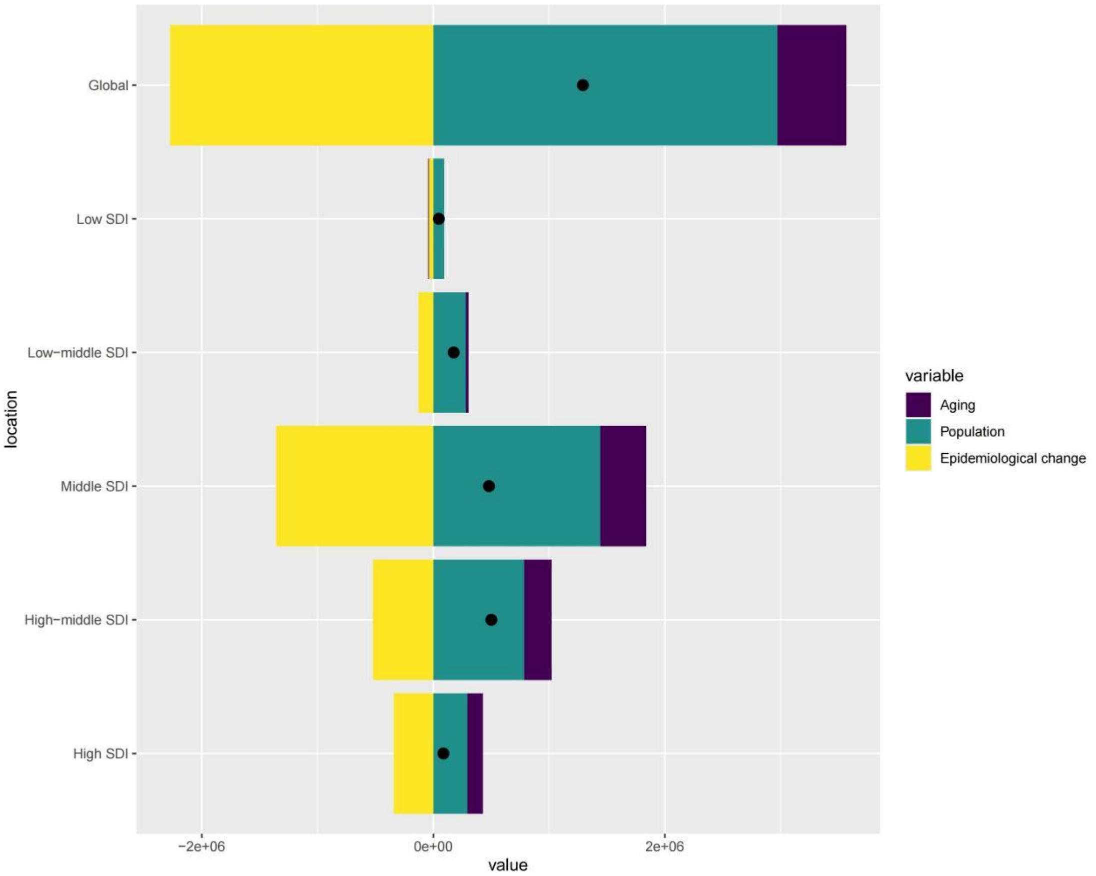

sFigure 6. Decomposition Analysis of Tobacco-Related Esophageal Cancer DALYs by Socio-demographic Index (SDI) Levels.

This figure presents a comprehensive decomposition analysis of tobacco-related esophageal cancer burden, measured in Disability-Adjusted Life Years (DALYs), across different Socio-demographic Index (SDI) levels. The visualization breaks down the DALY burden into three key contributing factors: population growth, population aging, and changes in age-specific rates. This three-factor decomposition framework reveals how demographic and epidemiological transitions influence tobacco-related esophageal cancer disability and premature mortality across the socioeconomic spectrum.

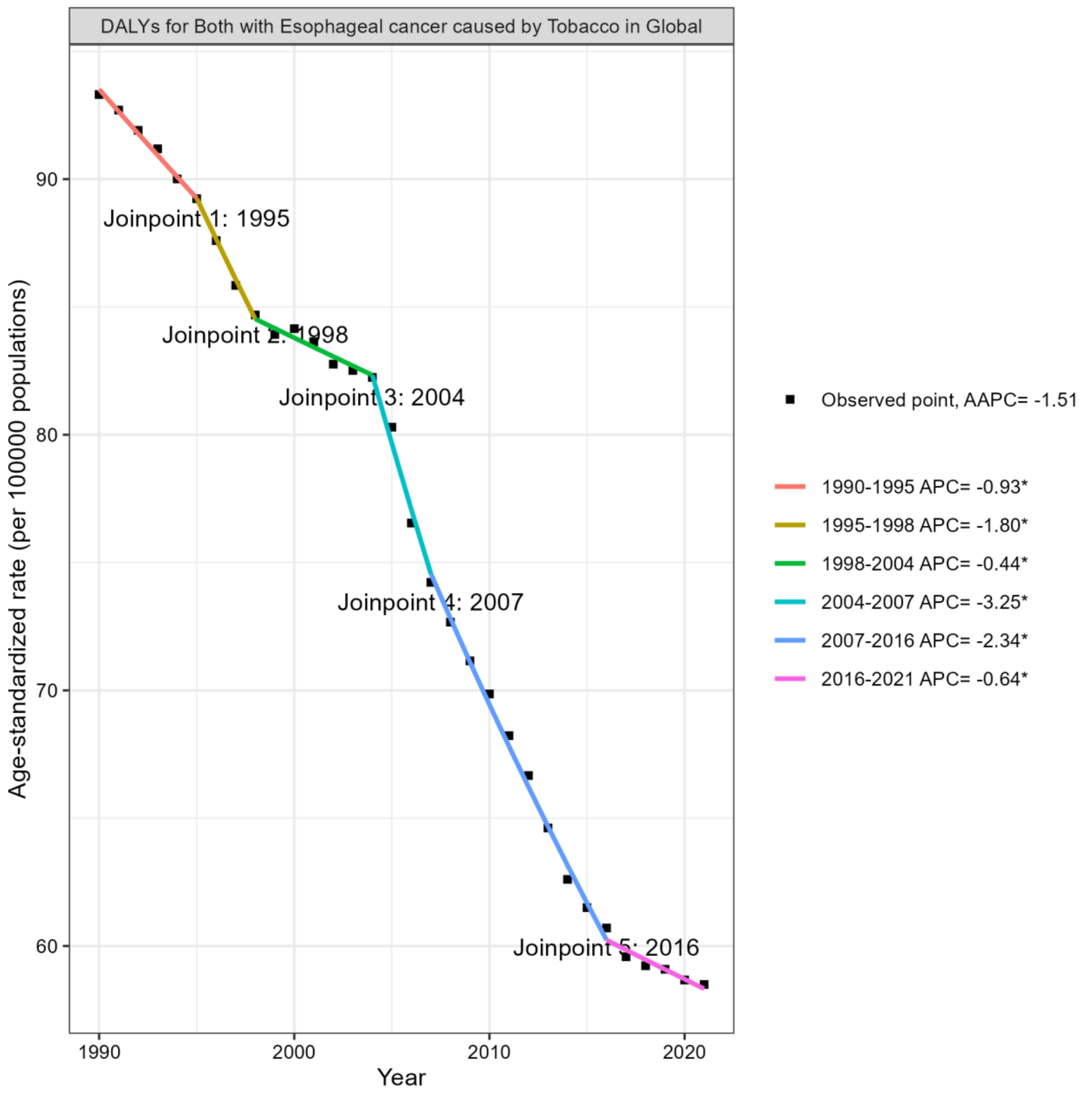

sFigure 7. Joinpoint Regression Analysis of Global Tobacco-Related Esophageal Cancer DALY Trends: Identification of Significant Trend Changes in Disease Burden (1990-2021).

This figure illustrates the temporal trends in global tobacco-related esophageal cancer disease burden, measured in Disability-Adjusted Life Years (DALYs), from 1990 to 2021 using joinpoint regression analysis. The visualization depicts the annual percentage change (APC) in DALY rates over the 32-year period, with identified joinpoints marking statistically significant changes in burden trends. Each segment between joinpoints represents a distinct period with a consistent rate of change.

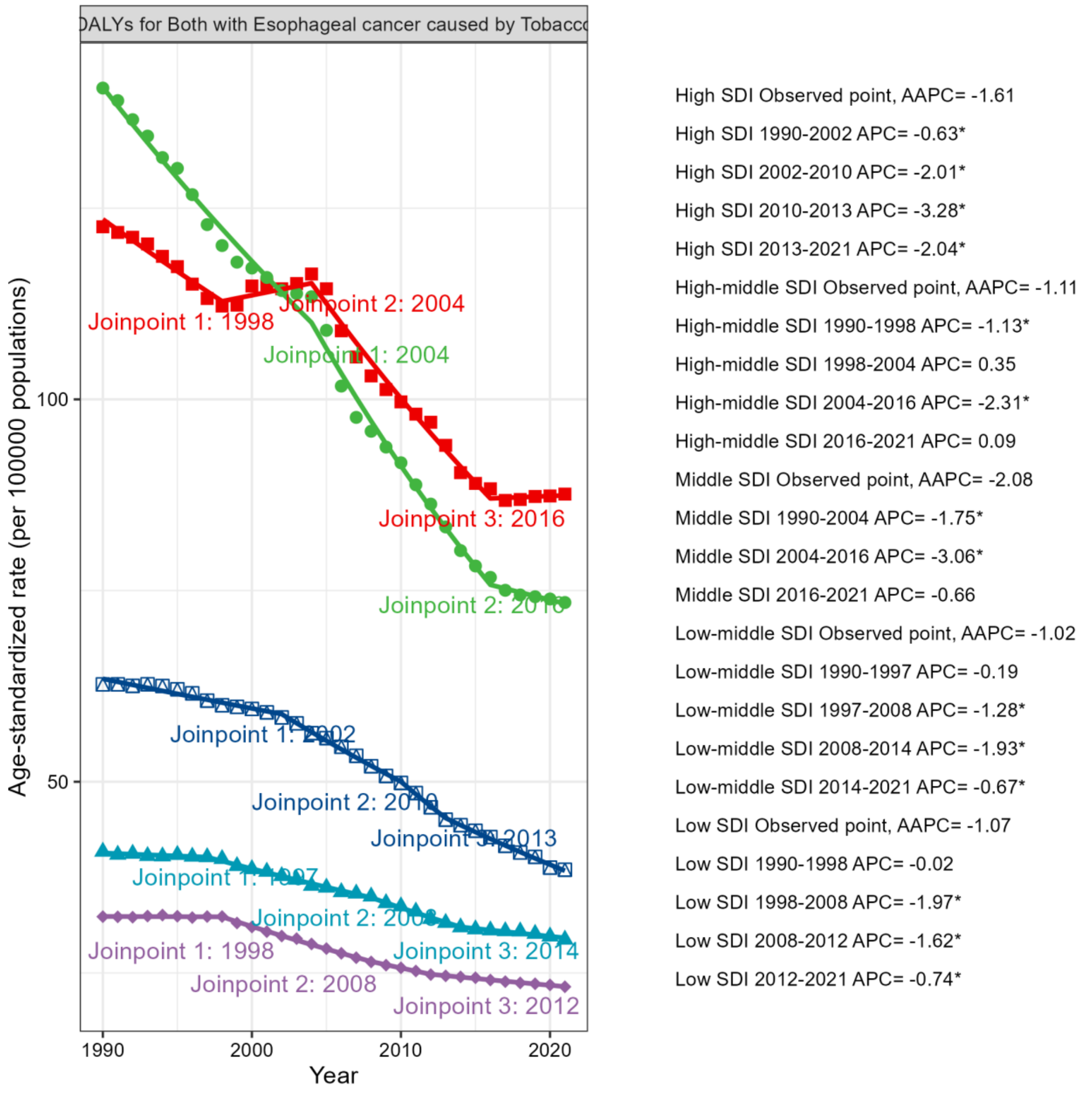

sFigure 8. Joinpoint Regression Analysis of Tobacco-Related Esophageal Cancer DALYs by Sociodemographic Index (SDI) Levels: Comparative Trend Analysis of Disease Burden Across Socioeconomic Strata (1990-2021).

This figure presents a comparative joinpoint regression analysis of tobacco-related esophageal cancer disease burden trends, measured in Disability-Adjusted Life Years (DALYs), across different Sociodemographic Index (SDI) levels from 1990 to 2021. The visualization depicts distinct DALY trajectories for high, high-middle, middle, low-middle, and low SDI regions, with identified joinpoints marking statistically significant changes in disease burden trends.

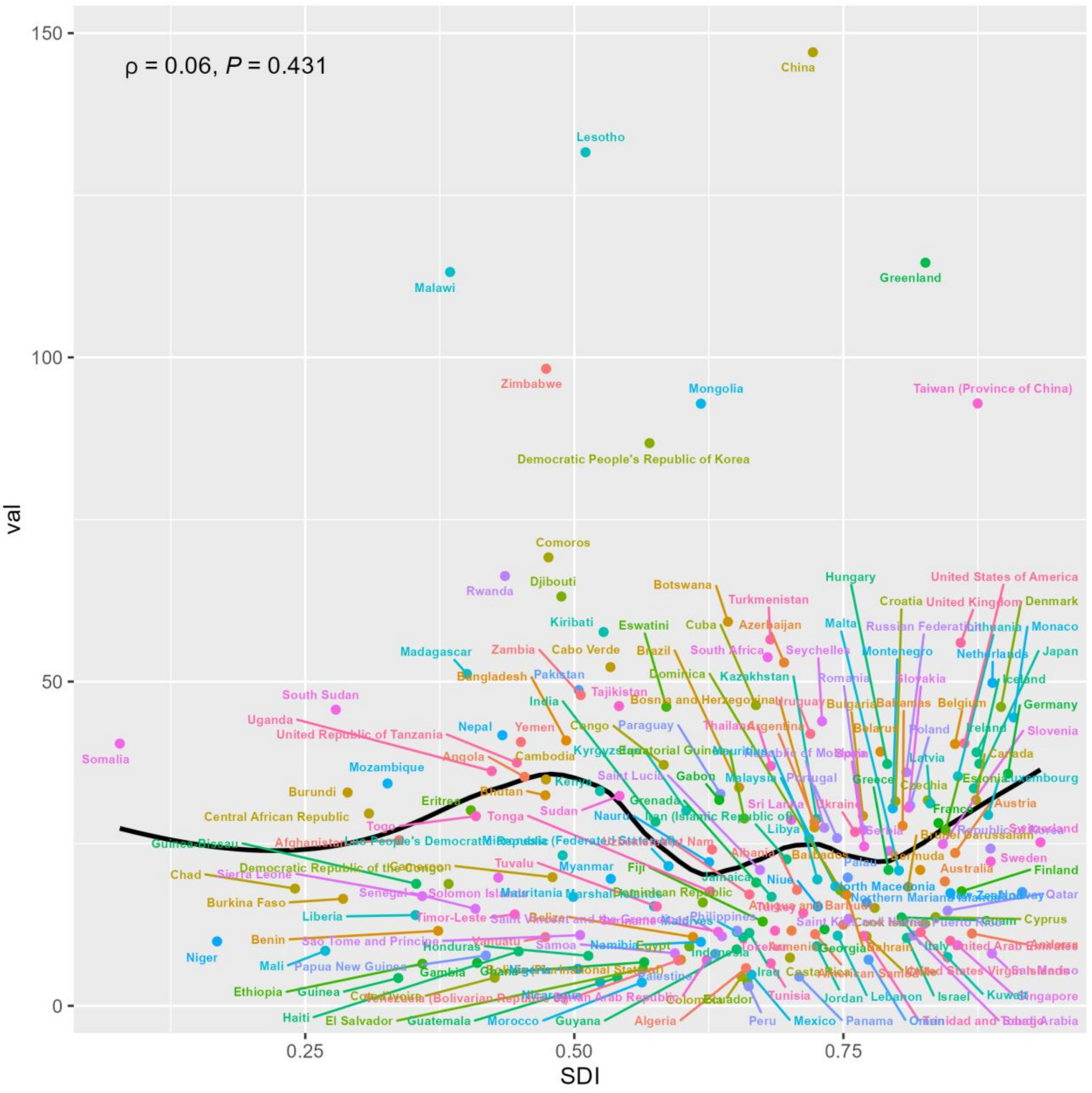

sFigure 9. Correlation between Sociodemographic Index (SDI) and Age-Standardized DALY Rates for Tobacco-Related Esophageal Cancer Across Countries: Global Socioeconomic Disparities in Cancer Burden.

This figure illustrates the relationship between national socioeconomic development status, as measured by the Sociodemographic Index (SDI), and the burden of tobacco-related esophageal cancer, represented by Age-Standardized DALY Rates. Each data point represents an individual country, allowing for cross-national comparison of cancer burden in relation to socioeconomic development.

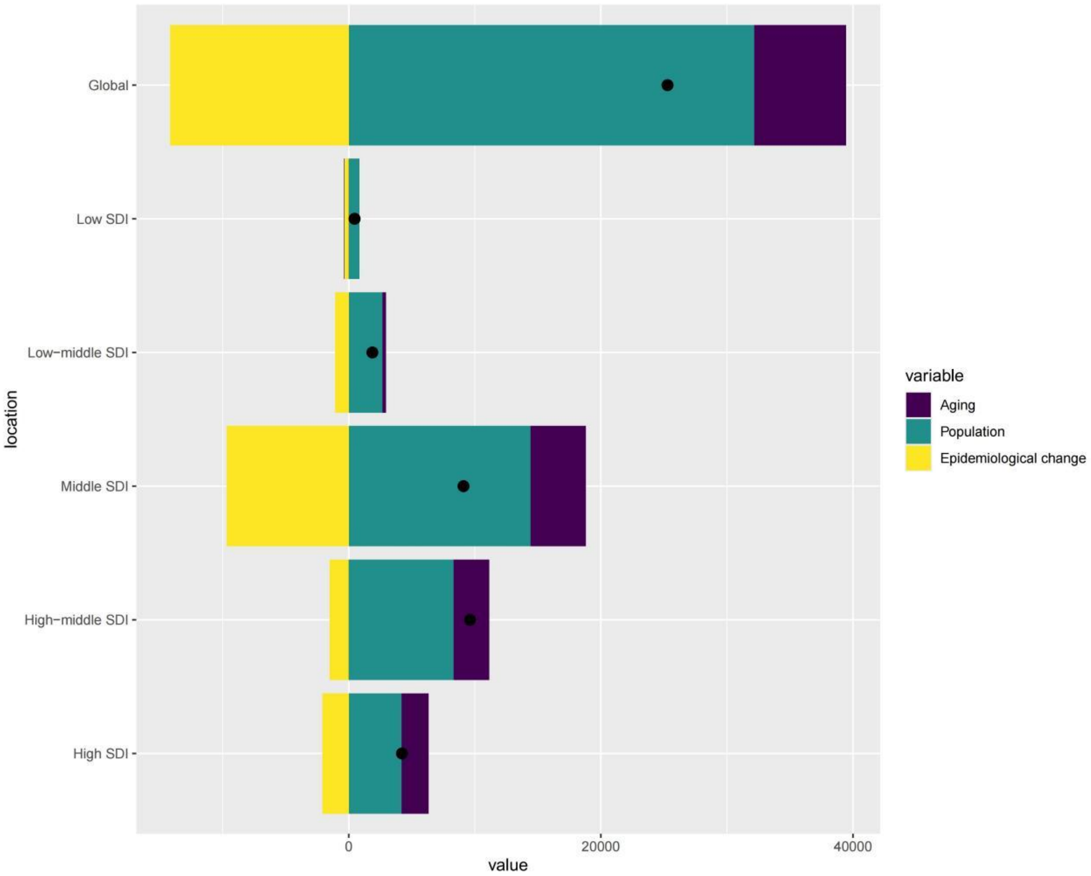

sFigure 10. Decomposition Analysis of Tobacco-Related Esophageal Cancer YLDs by Socio-demographic Index (SDI) Levels: Contributions of Population Growth, Population Aging, and Age-Specific Rates.

This figure presents a comprehensive decomposition analysis of tobacco-related esophageal cancer morbidity burden, measured in Years Lived with Disability (YLDs), across different Socio-demographic Index (SDI) levels. The visualization breaks down the YLD burden into three key contributing factors: population growth, population aging, and changes in age-specific rates. This three-factor decomposition framework reveals how demographic and epidemiological transitions influence the non-fatal burden of tobacco-related esophageal cancer across the socioeconomic spectrum.

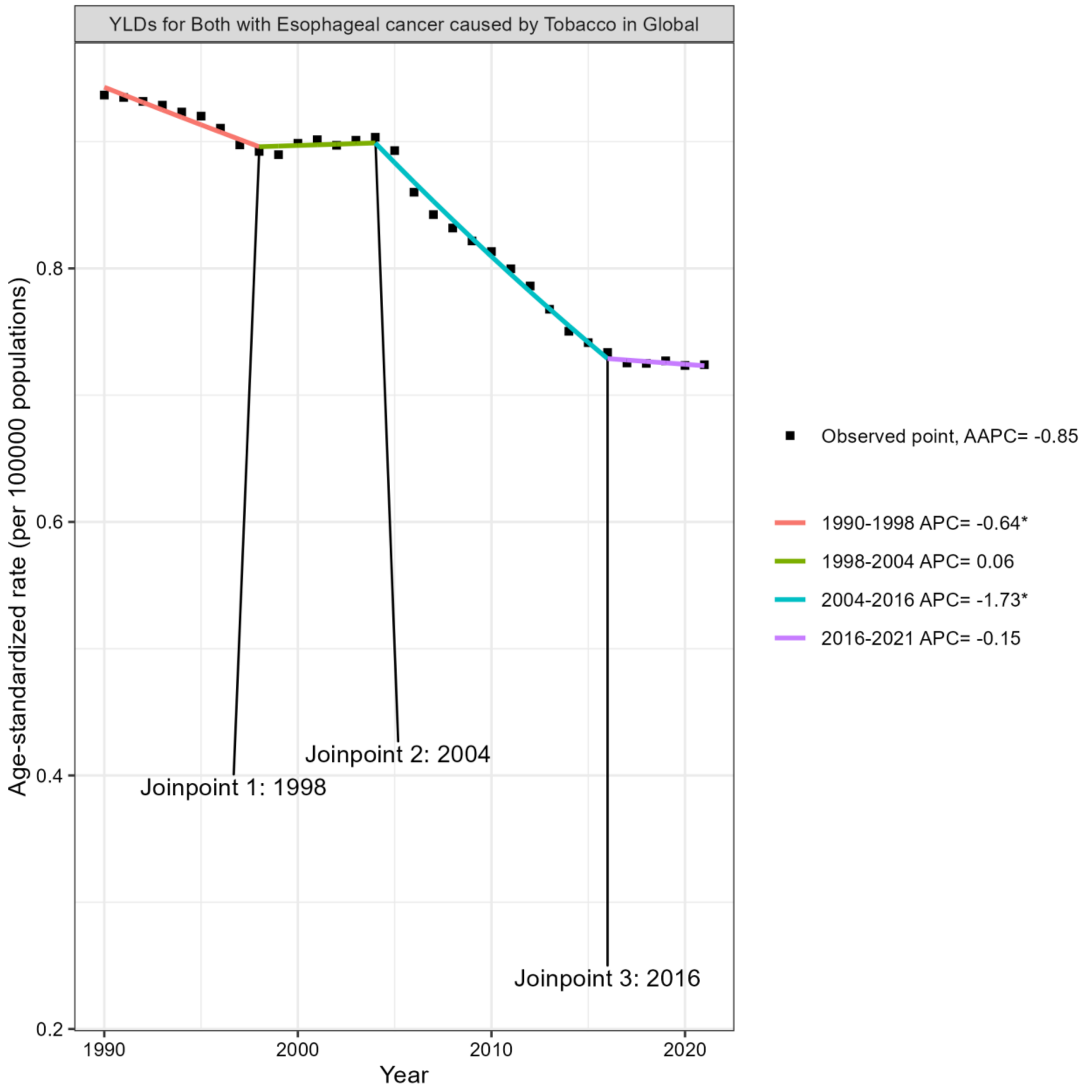

sFigure 11. Joinpoint Regression Analysis of Global Tobacco-Related Esophageal Cancer YLD Trends: Identification of Significant Trend Changes in Non-Fatal Disease Burden (1990-2021). This figure illustrates the temporal trends in global tobacco-related esophageal cancer non-fatal burden, measured in Years Lived with Disability (YLDs), from 1990 to 2021 using joinpoint regression analysis. The visualization depicts the annual percentage change (APC) in YLD rates over the 32-year period, with identified joinpoints marking statistically significant changes in morbidity burden trends. Each segment between joinpoints represents a distinct period with a consistent rate of change.

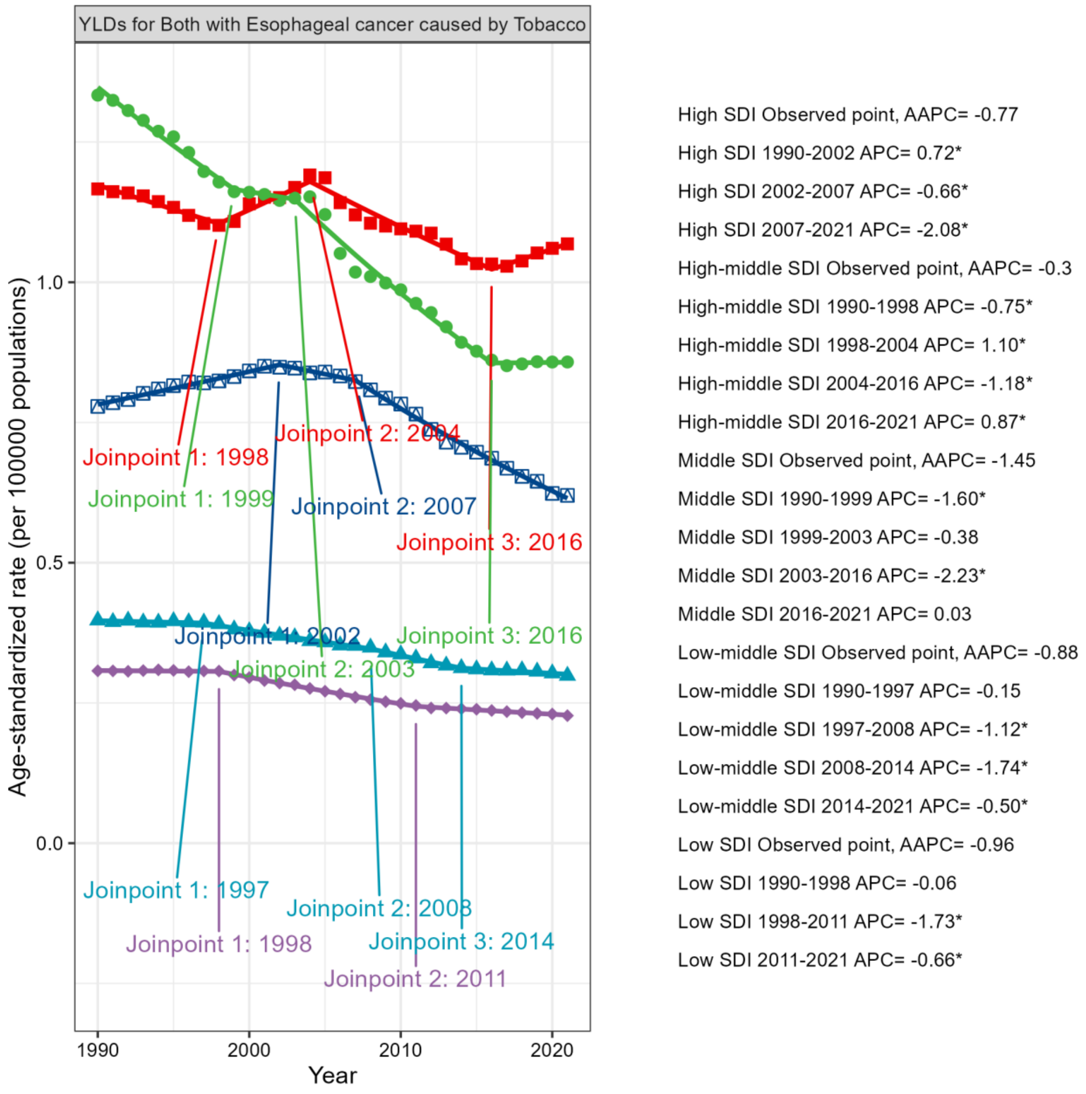

sFigure 12. Joinpoint Regression Analysis of Tobacco-Related Esophageal Cancer YLDs by Sociodemographic Index (SDI) Levels: Comparative Trend Analysis of Non-Fatal Disease Burden Across Socioeconomic Strata (1990-2021).

This figure presents a comparative joinpoint regression analysis of tobacco-related esophageal cancer non-fatal burden trends, measured in Years Lived with Disability (YLDs), across different Sociodemographic Index (SDI) levels from 1990 to 2021. The visualization depicts distinct YLD trajectories for high, high-middle, middle, low-middle, and low SDI regions, with identified joinpoints marking statistically significant changes in disability burden trends.

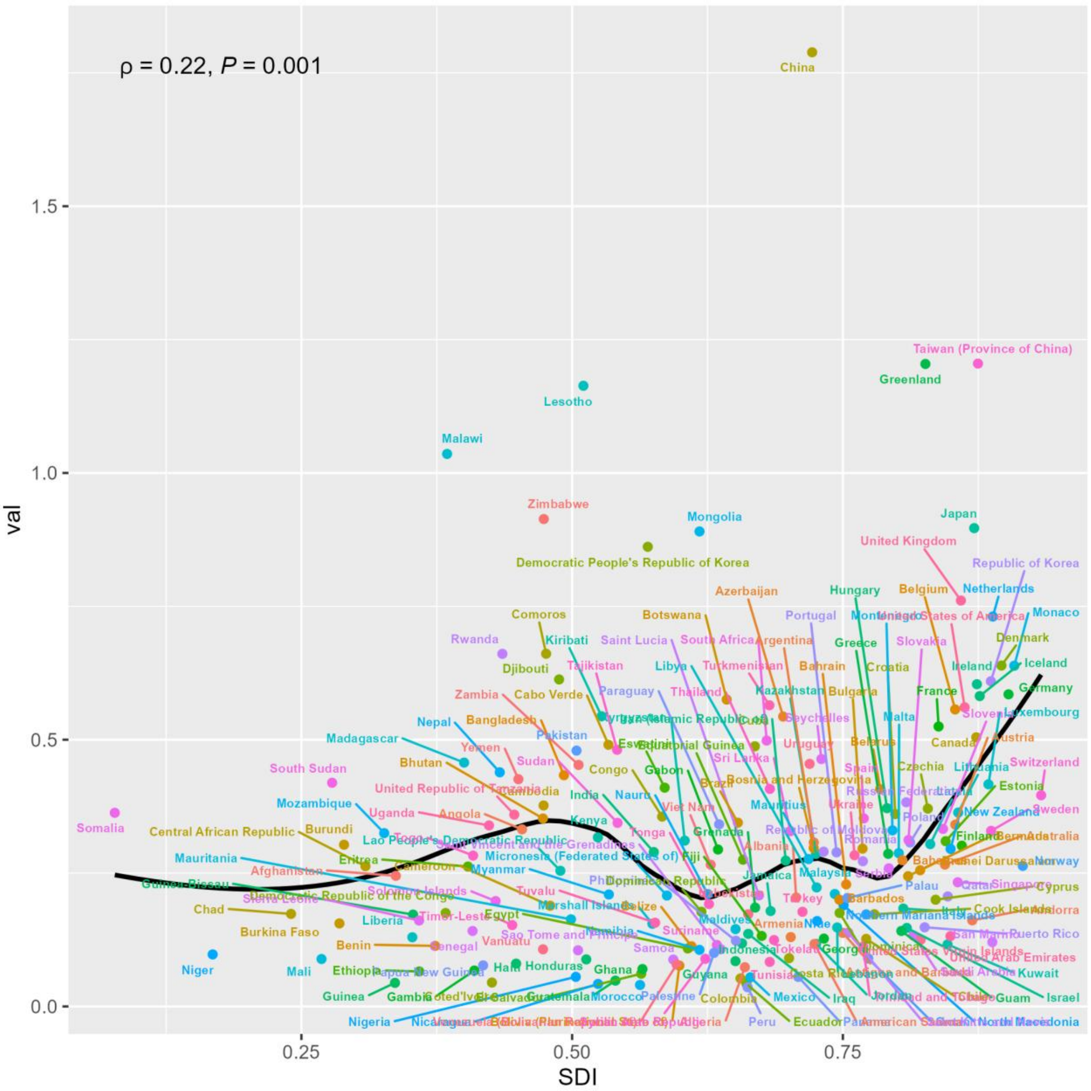

sFigure 13. Correlation between Sociodemographic Index (SDI) and Age-Standardized YLD Rates for Tobacco-Related Esophageal Cancer Across Countries: Global Socioeconomic Disparities in Non-Fatal Cancer Burden.

This figure illustrates the relationship between national socioeconomic development status, as measured by the Sociodemographic Index (SDI), and the non-fatal burden of tobacco-related esophageal cancer, represented by Age-Standardized YLD Rates. Each data point represents an individual country, allowing for cross-national comparison of cancer-related disability in relation to socioeconomic development.

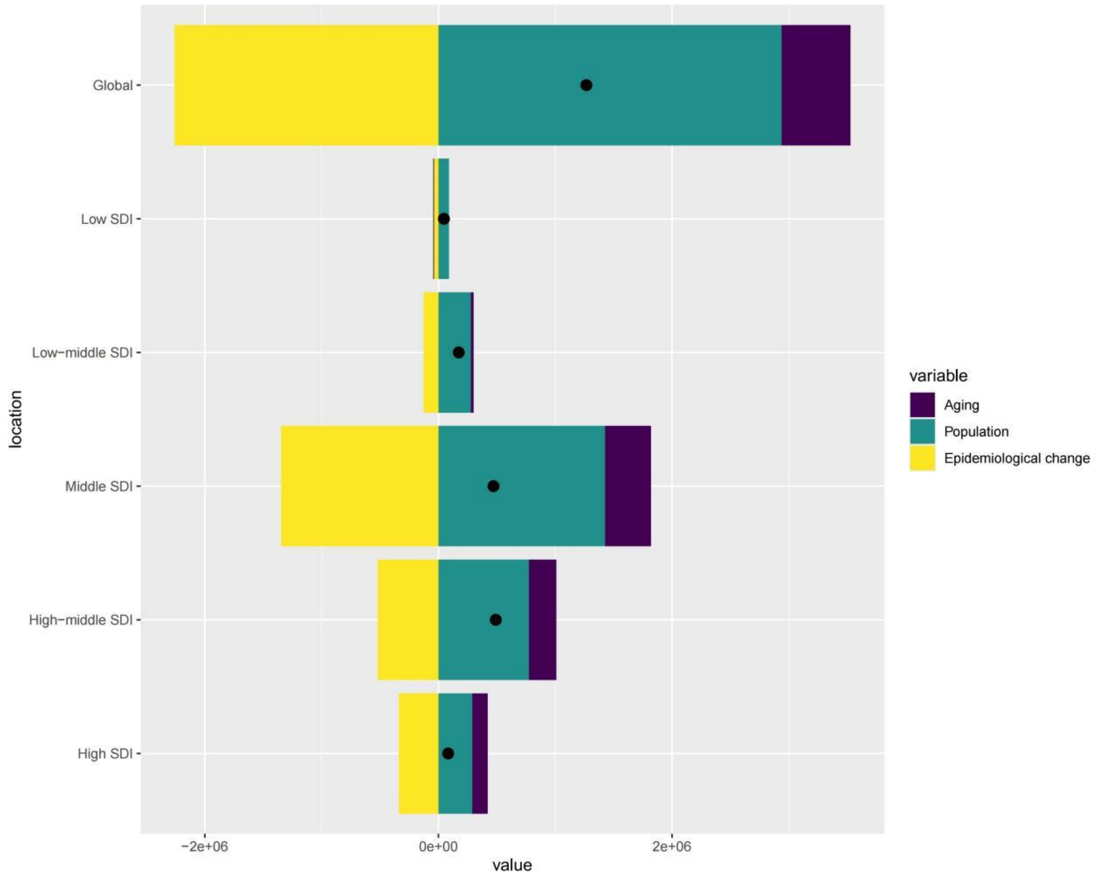

sFigure 14. Decomposition Analysis of Tobacco-Related Esophageal Cancer YLLs by Socio-demographic Index (SDI) Levels: Contributions of Population Growth, Population Aging, and Age-Specific Rates.

This figure presents a comprehensive decomposition analysis of tobacco-related esophageal cancer mortality burden, measured in Years of Life Lost (YLLs), across different Socio-demographic Index (SDI) levels. The visualization breaks down the YLL burden into three key contributing factors: population growth, population aging, and changes in age-specific rates. This three-factor decomposition framework reveals how demographic and epidemiological transitions influence the premature mortality burden of tobacco-related esophageal cancer across the socioeconomic spectrum.

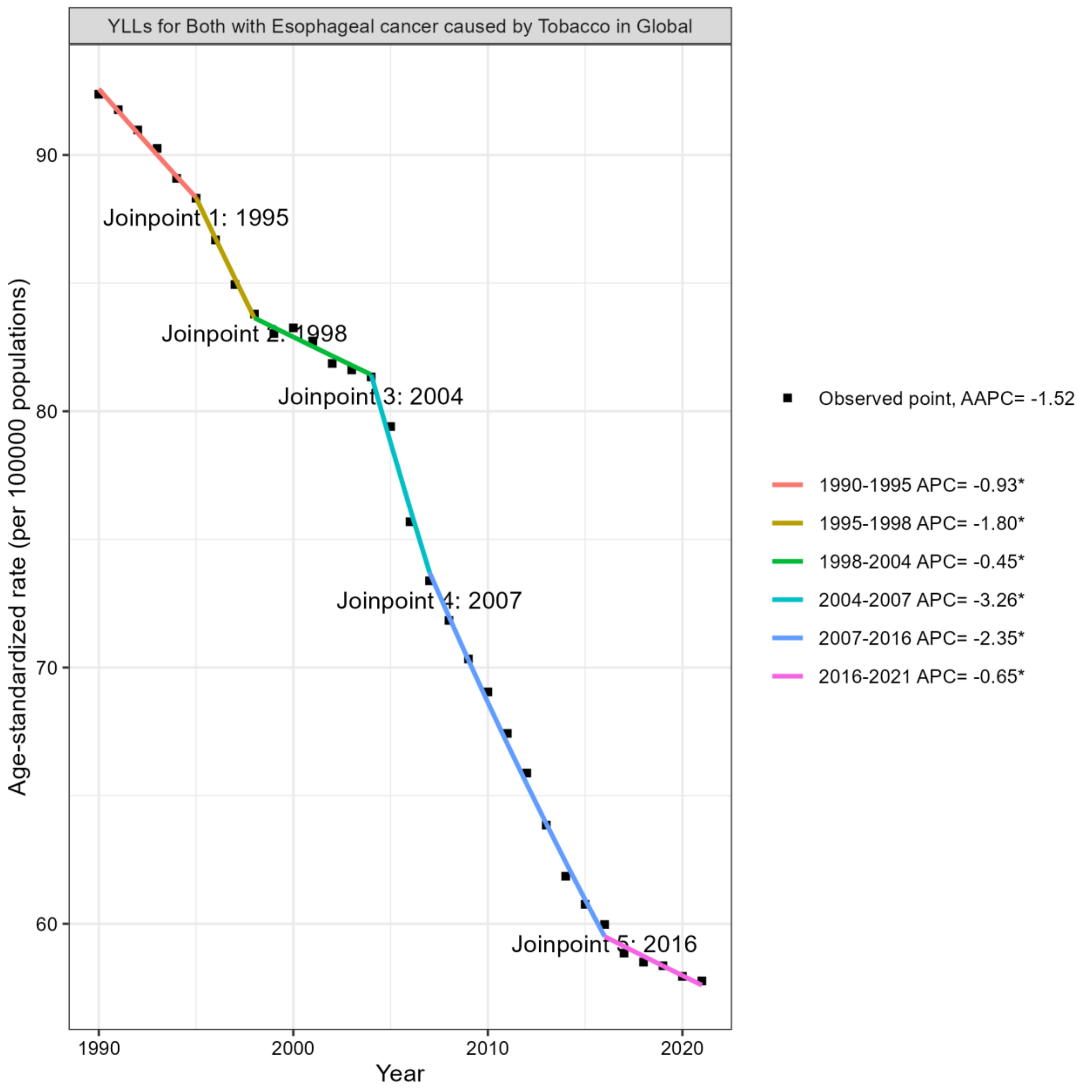

sFigure 15. Joinpoint Regression Analysis of Global Tobacco-Related Esophageal Cancer YLL Trends: Identification of Significant Trend Changes in Premature Mortality Burden (1990-2021). This figure illustrates the temporal trends in global tobacco-related esophageal cancer mortality burden, measured in Years of Life Lost (YLLs), from 1990 to 2021 using joinpoint regression analysis. The visualization depicts the annual percentage change (APC) in YLL rates over the 32-year period, with identified joinpoints marking statistically significant changes in premature mortality burden trends. Each segment between joinpoints represents a distinct period with a consistent rate of change.

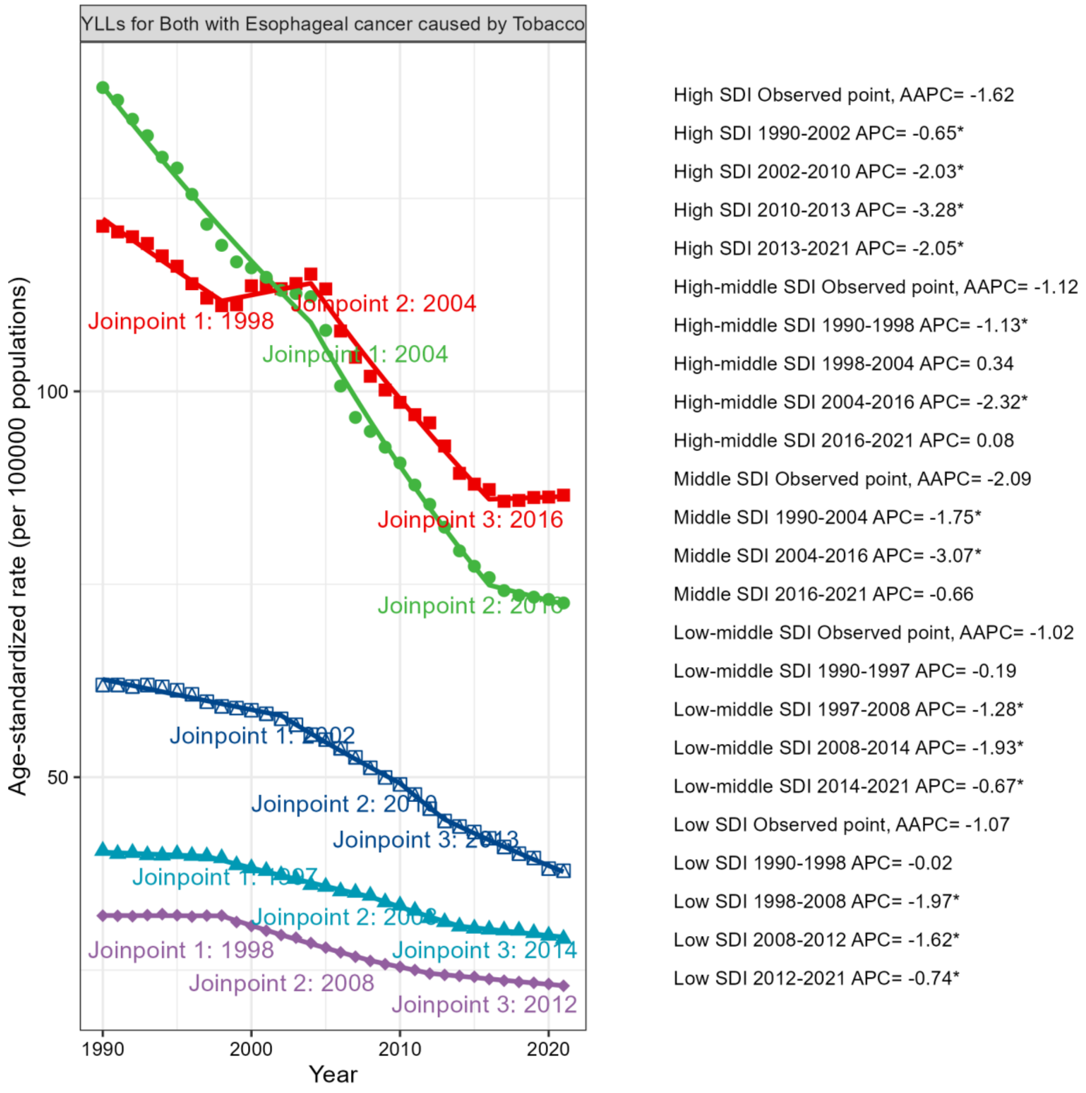

sFigure 16. Joinpoint Regression Analysis of Tobacco-Related Esophageal Cancer YLLs by Sociodemographic Index (SDI) Levels: Comparative Trend Analysis of Premature Mortality Burden Across Socioeconomic Strata (1990-2021).

This figure presents a comparative joinpoint regression analysis of tobacco-related esophageal cancer mortality burden trends, measured in Years of Life Lost (YLLs), across different Sociodemographic Index (SDI) levels from 1990 to 2021. The visualization depicts distinct YLL trajectories for high, high-middle, middle, low-middle, and low SDI regions, with identified joinpoints marking statistically significant changes in premature mortality burden trends.

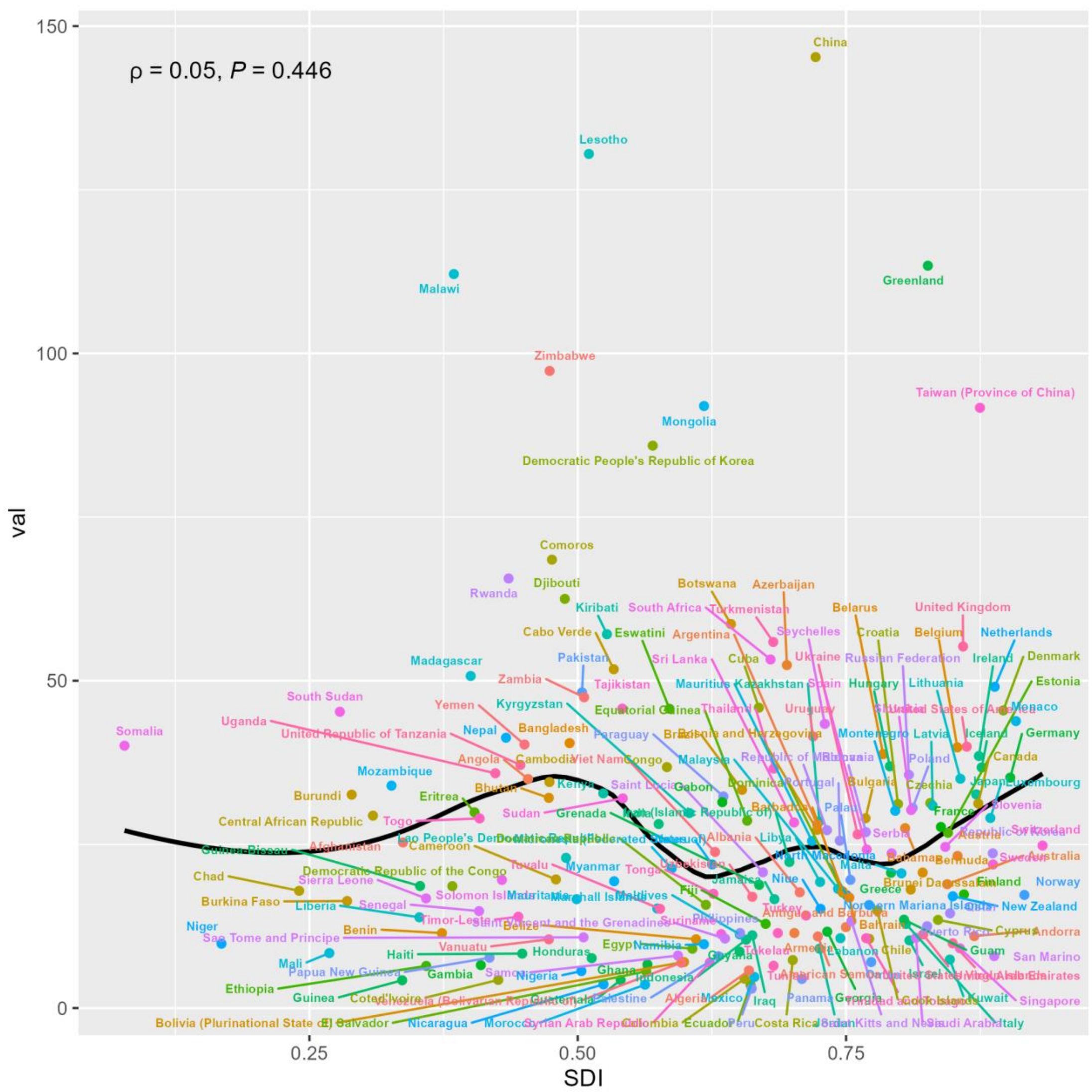

sFigure 17. Correlation between Sociodemographic Index (SDI) and Age-Standardized YLL Rates for Tobacco-Related Esophageal Cancer Across Countries: Global Socioeconomic Disparities in Non-Fatal Cancer Burden.

This figure illustrates the relationship between national socioeconomic development status, as measured by the Sociodemographic Index (SDI), and the non-fatal burden of tobacco-related esophageal cancer, represented by Age-Standardized YLL Rates. Each data point represents an individual country, allowing for cross-national comparison of cancer-related disability in relation to socioeconomic development.

sTable 1 Tobacco-Related Esophageal Cancer Deaths Between 1990 and 2021 for Both Sexes, Each SDI Regions and All countries, With EAPC Between 1990 and 2021.

	1990 Deaths		2021 Deaths		
	Case number (95% UI)	ASR/100,000 persons (95% UI)	Case number (95% UI)	ASR/100,000 persons (95% UI)	EAPC% (95% CI) 1990–2021
Global	143332.8 (117012.5 , 170295)	3.6 (3 , 4.3)	219185.3 (172166.4 , 270731.2)	2.5 (2 , 3.1)	-1.33 (-1.44 , -1.22)
**Sex**					
Male	128460.4 (104173.7 , 153779.4)	7.1 (5.8 , 8.5)	201411.2 (157094.9 , 250086.6)	5.1 (4 , 6.3)	-1.24 (-1.34 , -1.13)
Female	14872.4 (11144.9 , 18530.2)	0.7 (0.5 , 0.9)	17774.1 (13596.8 , 22344.1)	0.4 (0.3 , 0.5)	-2.37 (-2.55 , -2.19)
**SDI**					
High SDI	27941.6 (22890.9 , 32511.2)	2.5 (2.1 , 2.9)	36045.4 (28280.5 , 43800.9)	1.7 (1.3 , 2)	-1.45 (-1.59 , -1.32)
High-middle SDI	46617.9 (37143.7 , 56995.5)	4.7 (3.7 , 5.7)	75223.5 (56618.2 , 98193.9)	3.7 (2.8 , 4.9)	-0.85 (-0.99 , -0.71)
Middle SDI	56459.5 (44978 , 69933.3)	5.5 (4.4 , 6.8)	86733.1 (65047.8 , 112366.3)	3.3 (2.5 , 4.3)	-1.87 (-1.98 , -1.76)
Low-middle SDI	9478.5 (7884.3 , 11286.1)	1.6 (1.3 , 1.9)	16581.5 (13541.9 , 19828.7)	1.2 (1 , 1.4)	-1.1 (-1.18 , -1.02)
Low SDI	2773.9 (2252.8 , 3320)	1.3 (1 , 1.5)	4524 (3601.5 , 5573.8)	0.9 (0.7 , 1.1)	-1.2 (-1.29 , -1.11)
**Country**					
Afghanistan	76.2 (44.5 , 120.7)	1.1 (0.7 , 1.8)	98.2 (60.5 , 148.5)	1.1 (0.7 , 1.6)	-0.31 (-0.67 , 0.04)
Albania	19.1 (14 , 24.6)	1 (0.7 , 1.3)	34.4 (23.1 , 47.6)	0.8 (0.5 , 1.1)	-0.36 (-0.61 , -0.1)
Algeria	32.9 (24.6 , 42.4)	0.3 (0.2 , 0.4)	88.1 (60 , 121.2)	0.3 (0.2 , 0.4)	-0.31 (-0.39 , -0.23)
American Samoa	0.1 (0.1 , 0.1)	0.4 (0.2 , 0.5)	0.2 (0.1 , 0.3)	0.4 (0.3 , 0.6)	0.97 (0.64 , 1.3)
Andorra	0.6 (0.3 , 0.9)	1 (0.6 , 1.5)	0.7 (0.4 , 1.2)	0.5 (0.3 , 0.7)	-2.08 (-2.38 , -1.79)
Angola	83.6 (53.5 , 121.2)	2.1 (1.4 , 3)	160 (111.3 , 225.2)	1.4 (0.9 , 1.9)	-1.56 (-1.83 , -1.29)
Antigua and Barbuda	0.4 (0.3 , 0.5)	0.7 (0.5 , 0.9)	0.6 (0.4 , 0.8)	0.6 (0.4 , 0.7)	-0.58 (-0.87 , -0.29)
Argentina	794.7 (604.8 , 991.1)	2.4 (1.9 , 3)	664 (488.3 , 852.5)	1.2 (0.9 , 1.5)	-2.24 (-2.52 , -1.96)
Armenia	36.2 (30.5 , 41.5)	1.3 (1.1 , 1.5)	22.3 (18.3 , 26.8)	0.5 (0.4 , 0.6)	-3.1 (-3.44 , -2.75)
Australia	317.2 (243.1 , 395.8)	1.6 (1.2 , 2)	395.4 (278 , 530.7)	0.9 (0.6 , 1.1)	-2.13 (-2.23 , -2.03)
Austria	124.6 (96.8 , 153.5)	1.1 (0.8 , 1.3)	174.3 (136.8 , 218.6)	1 (0.8 , 1.2)	-0.09 (-0.23 , 0.04)
Azerbaijan	146.8 (107.3 , 188.3)	3 (2.2 , 3.8)	221.7 (153 , 298.9)	2.3 (1.6 , 3.1)	-0.4 (-0.64 , -0.15)
Bahamas	2.2 (1.4 , 3.2)	1.4 (0.9 , 2.1)	4.5 (3 , 6.3)	1.1 (0.7 , 1.6)	-0.23 (-0.51 , 0.06)
Bahrain	2.3 (1.6 , 3.1)	1.8 (1.2 , 2.3)	5.5 (3.6 , 8)	0.9 (0.6 , 1.3)	-2.97 (-3.33 , -2.61)
Bangladesh	1309 (886.4 , 1825.5)	2.9 (1.9 , 4)	2284.2 (1521.8 , 3240.6)	1.7 (1.1 , 2.4)	-1.62 (-1.78 , -1.45)
Barbados	4 (2.8 , 5.3)	1.3 (0.9 , 1.7)	4.5 (2.9 , 6.4)	0.9 (0.6 , 1.2)	-1.59 (-1.82 , -1.36)
Belarus	186.5 (149.9 , 224.1)	1.4 (1.1 , 1.7)	228.8 (167.3 , 291.6)	1.4 (1 , 1.8)	-0.4 (-0.56 , -0.24)
Belgium	315.2 (249.9 , 377.3)	2.1 (1.7 , 2.5)	406.9 (313.8 , 510.3)	1.7 (1.3 , 2.2)	-0.57 (-0.78 , -0.35)
Belize	0.4 (0.3 , 0.5)	0.4 (0.3 , 0.6)	1.3 (0.9 , 1.6)	0.4 (0.3 , 0.6)	0.29 (-0.2 , 0.77)
Benin	7.6 (5.6 , 10.5)	0.4 (0.3 , 0.5)	23.1 (15.9 , 31.5)	0.5 (0.3 , 0.6)	0.97 (0.8 , 1.14)
Bermuda	1 (0.7 , 1.4)	1.6 (1.1 , 2.2)	1.2 (0.8 , 1.7)	0.9 (0.6 , 1.2)	-1.39 (-1.68 , -1.09)
Bhutan	4.6 (3 , 6.5)	2 (1.3 , 2.9)	8.5 (5.8 , 11.9)	1.5 (1 , 2.1)	-1.12 (-1.33 , -0.92)
Bolivia (Plurinational State of)	13.7 (9.3 , 20.1)	0.5 (0.3 , 0.7)	27.7 (17.5 , 41.4)	0.3 (0.2 , 0.5)	-0.74 (-0.97 , -0.51)
Bosnia and Herzegovina	45.3 (34.1 , 58.2)	1.1 (0.8 , 1.4)	68.4 (46.7 , 92.6)	1.1 (0.7 , 1.5)	0.12 (-0.04 , 0.29)
Botswana	19.7 (13.3 , 27.9)	3.6 (2.5 , 5.2)	34 (23.7 , 47.6)	2.4 (1.7 , 3.4)	-1.56 (-1.83 , -1.29)

Brazil	2868.1 (2288.4 , 3429.9)	3.3 (2.6 , 4)	3493.3 (2666.8 , 4418.7)	1.4 (1.1 , 1.8)	-2.97 (-3.06 , -2.87)
Brunei Darussalam	1.5 (1.1 , 2)	1.6 (1.2 , 2.2)	2.6 (1.8 , 3.5)	0.8 (0.6 , 1.1)	-1.86 (-2.08 , -1.63)
Bulgaria	151.4 (119 , 184.4)	1.2 (0.9 , 1.5)	134.3 (100.2 , 169.5)	1 (0.8 , 1.3)	-0.74 (-0.96 , -0.53)
Burkina Faso	15.9 (11.4 , 21.9)	0.4 (0.3 , 0.5)	57.4 (37.6 , 81.1)	0.6 (0.4 , 0.9)	2.33 (2.1 , 2.56)
Burundi	75.4 (53.3 , 101.1)	3.2 (2.3 , 4.3)	61 (42.4 , 83.6)	1.2 (0.9 , 1.7)	-3.58 (-3.84 , -3.32)
Cabo Verde	2.8 (2.1 , 3.7)	1.3 (0.9 , 1.6)	8.9 (6.2 , 12.1)	2 (1.4 , 2.7)	0.63 (0.13 , 1.14)
Cambodia	96.9 (72.6 , 123.9)	2.3 (1.7 , 3)	174.8 (125.6 , 229.2)	1.5 (1.1 , 2)	-1.58 (-1.68 , -1.47)
Cameroon	20.7 (14.2 , 28.7)	0.5 (0.3 , 0.6)	95.1 (59.4 , 142.4)	0.7 (0.5 , 1.1)	2.04 (1.8 , 2.28)
Canada	671.1 (537.6 , 802)	2 (1.6 , 2.4)	1086.9 (822.3 , 1384.4)	1.4 (1.1 , 1.8)	-1.14 (-1.28 , -1)
Central African Republic	21.1 (14.2 , 29.7)	1.8 (1.2 , 2.5)	25.4 (16.9 , 36.7)	1.1 (0.7 , 1.5)	-1.76 (-1.92 , -1.6)
Chad	9.9 (6.9 , 13.2)	0.4 (0.3 , 0.5)	40.4 (26.9 , 59.8)	0.7 (0.5 , 1.1)	2.75 (2.52 , 2.97)
Chile	161.2 (120.3 , 206)	1.6 (1.2 , 2.1)	114.8 (85.6 , 149.6)	0.4 (0.3 , 0.6)	-4.52 (-4.79 , -4.26)
China	85093 (65881 , 106500.2)	10.5 (8.1 , 13)	140513.7 (103904.8 , 183437)	6.6 (4.9 , 8.7)	-1.63 (-1.79 , -1.47)
Colombia	143.5 (110.7 , 180.6)	0.9 (0.7 , 1.1)	112.6 (79.9 , 153)	0.2 (0.1 , 0.3)	-5.32 (-5.56 , -5.07)
Comoros	8.3 (5.7 , 11.8)	4.2 (3 , 5.9)	13.2 (9.1 , 19.4)	2.8 (1.9 , 4)	-1.77 (-1.92 , -1.62)
Congo	21.1 (14 , 31.3)	2 (1.3 , 3)	38.7 (25.3 , 55.7)	1.5 (1 , 2.1)	-1 (-1.23 , -0.76)
Cook Islands	0.1 (0.1 , 0.1)	0.9 (0.6 , 1.2)	0.2 (0.1 , 0.2)	0.6 (0.4 , 0.9)	-1.22 (-1.34 , -1.09)
Costa Rica	14.1 (10.6 , 17.9)	0.8 (0.6 , 1.1)	19.2 (13.8 , 25.6)	0.4 (0.3 , 0.5)	-3.02 (-3.23 , -2.82)
Coted'Ivoire	6.8 (4.8 , 9.3)	0.2 (0.1 , 0.2)	18.6 (12.5 , 26.7)	0.2 (0.1 , 0.2)	-0.41 (-0.75 , -0.07)
Croatia	130.9 (105.1 , 155.3)	2.1 (1.7 , 2.5)	110.2 (83.4 , 136.6)	1.3 (1 , 1.6)	-1.51 (-1.67 , -1.34)
Cuba	165.4 (128.3 , 203.1)	1.6 (1.3 , 2)	355.5 (263.3 , 453)	1.8 (1.3 , 2.3)	0.62 (0.5 , 0.74)
Cyprus	4.9 (3.5 , 6.6)	0.7 (0.5 , 0.9)	12.8 (8.9 , 17.2)	0.6 (0.4 , 0.8)	0.25 (0.03 , 0.47)
Czechia	182.1 (141.9 , 225.1)	1.3 (1 , 1.6)	260.7 (189.7 , 333.7)	1.2 (0.9 , 1.6)	-0.33 (-0.44 , -0.23)
Democratic People's Republic of Korea	621.5 (408.2 , 881.1)	3.8 (2.5 , 5.3)	1082.8 (736.3 , 1569.7)	3.2 (2.2 , 4.6)	-0.52 (-0.62 , -0.42)
Democratic Republic of the Congo	164.3 (108.3 , 227.9)	1 (0.7 , 1.4)	265.1 (162.8 , 383.8)	0.7 (0.4 , 1)	-1.33 (-1.51 , -1.15)
Denmark	229 (189.4 , 268)	2.8 (2.3 , 3.3)	266.9 (204.4 , 329.8)	2.2 (1.7 , 2.7)	-1.16 (-1.33 , -0.99)
Djibouti	4.3 (2.7 , 6.5)	3.3 (2.1 , 4.9)	15.6 (9.5 , 23.7)	2.6 (1.6 , 3.9)	-0.78 (-0.86 , -0.7)
Dominica	0.5 (0.3 , 0.6)	0.8 (0.6 , 1.1)	0.6 (0.4 , 0.9)	0.7 (0.5 , 1)	-0.26 (-0.51 , 0)
Dominican Republic	25.2 (17.3 , 34.4)	0.8 (0.5 , 1.1)	69.3 (46.6 , 99.4)	0.7 (0.5 , 1)	0.22 (-0.01 , 0.44)
Ecuador	23.3 (18 , 29.5)	0.5 (0.4 , 0.6)	27.8 (19.4 , 38.4)	0.2 (0.1 , 0.2)	-2.73 (-3.04 , -2.42)
Egypt	97.4 (72.6 , 125.8)	0.4 (0.3 , 0.5)	225.4 (164.6 , 302.1)	0.4 (0.3 , 0.6)	0.07 (-0.1 , 0.25)
El Salvador	6.4 (4.5 , 9.1)	0.2 (0.2 , 0.3)	14 (9.3 , 20.1)	0.2 (0.2 , 0.3)	-0.14 (-0.37 , 0.08)
Equatorial Guinea	3.1 (2.1 , 4.6)	1.5 (1 , 2.2)	5.6 (3.4 , 8.1)	1.1 (0.7 , 1.6)	-1.14 (-1.68 , -0.59)
Eritrea	26.6 (15.7 , 38.8)	2 (1.2 , 2.8)	32.3 (19.1 , 49.7)	1.1 (0.6 , 1.6)	-2.5 (-2.7 , -2.29)
Estonia	27.9 (21.9 , 34.4)	1.3 (1.1 , 1.7)	26.1 (19.3 , 33.5)	1 (0.8 , 1.3)	-1.04 (-1.23 , -0.85)
Eswatini	6.4 (4.4 , 8.9)	2.3 (1.6 , 3.2)	9.7 (6.3 , 13.9)	1.8 (1.2 , 2.5)	-0.76 (-1.33 , -0.18)
Ethiopia	119.4 (81.1 , 176.4)	0.6 (0.4 , 0.9)	109.1 (77.7 , 156.2)	0.3 (0.2 , 0.4)	-2.64 (-2.95 , -2.33)
Fiji	2.1 (1.5 , 2.9)	0.6 (0.4 , 0.8)	3.9 (2.7 , 5.5)	0.5 (0.3 , 0.7)	-0.62 (-0.74 , -0.49)
Finland	72.3 (53 , 94)	1 (0.8 , 1.3)	89.2 (63.5 , 116.9)	0.7 (0.5 , 0.9)	-1 (-1.09 , -0.91)
France	2835.4 (2199.2 , 3406)	3.5 (2.8 , 4.3)	1627.6 (1221.7 , 2066.1)	1.2 (0.9 , 1.5)	-3.53 (-3.65 , -3.41)
Gabon	7.1 (4.9 , 10)	1.2 (0.9 , 1.7)	13 (8.9 , 18.1)	1.2 (0.8 , 1.6)	-0.18 (-0.29 , -0.08)

Gambia	0.9 (0.6 , 1.3)	0.3 (0.2 , 0.4)	2.4 (1.6 , 3.4)	0.3 (0.2 , 0.3)	-0.26 (-0.39 , -0.13)
Georgia	56.8 (43.9 , 69.6)	0.9 (0.7 , 1.1)	28.7 (22.1 , 35.8)	0.5 (0.4 , 0.6)	-0.88 (-1.44 , -0.32)
Germany	1983.5 (1553.5 , 2397.8)	1.6 (1.3 , 1.9)	2801 (2125.2 , 3483.1)	1.5 (1.1 , 1.8)	-0.48 (-0.66 , -0.31)
Ghana	12 (8.4 , 16.2)	0.2 (0.1 , 0.3)	44.1 (30.3 , 59.6)	0.3 (0.2 , 0.4)	1.69 (1.47 , 1.91)
Greece	188.1 (156 , 220.6)	1.2 (1 , 1.4)	207.9 (167.1 , 250.8)	0.9 (0.7 , 1)	-1.28 (-1.46 , -1.09)
Greenland	3 (2.2 , 4)	9.1 (6.6 , 12)	3.4 (2.3 , 4.8)	4.7 (3.2 , 6.8)	-2.02 (-2.11 , -1.94)
Grenada	0.8 (0.6 , 1.1)	1.2 (0.9 , 1.6)	0.9 (0.6 , 1.1)	0.7 (0.5 , 0.9)	-1.36 (-1.96 , -0.76)
Guam	0.4 (0.3 , 0.6)	0.5 (0.4 , 0.8)	1 (0.7 , 1.3)	0.5 (0.3 , 0.6)	0.21 (-0.15 , 0.58)
Guatemala	10.1 (7.5 , 13.3)	0.3 (0.3 , 0.5)	20.7 (14.7 , 28.2)	0.2 (0.1 , 0.3)	-2.13 (-2.49 , -1.77)
Guinea	4.8 (3.4 , 6.6)	0.1 (0.1 , 0.2)	9.3 (5.9 , 13.8)	0.2 (0.1 , 0.2)	0.71 (0.52 , 0.89)
Guinea-Bissau	1.4 (0.9 , 2)	0.3 (0.2 , 0.5)	5.2 (3.5 , 7.2)	0.7 (0.5 , 0.9)	3.2 (2.8 , 3.61)
Guyana	1.4 (1 , 1.8)	0.4 (0.3 , 0.5)	2.1 (1.4 , 3)	0.3 (0.2 , 0.5)	0 (-0.19 , 0.18)
Haiti	19.3 (12.9 , 28.1)	0.6 (0.4 , 0.8)	23 (14.6 , 34.9)	0.3 (0.2 , 0.5)	-1.83 (-2.01 , -1.65)
Honduras	4.9 (3.5 , 6.7)	0.3 (0.2 , 0.4)	20.8 (14.2 , 29)	0.4 (0.2 , 0.5)	1.46 (1.25 , 1.67)
Hungary	304.4 (241.1 , 362.2)	2.1 (1.7 , 2.5)	248.6 (191.3 , 317.2)	1.4 (1.1 , 1.7)	-1.8 (-2.15 , -1.45)
Iceland	7.4 (5.8 , 8.9)	2.6 (2 , 3.1)	9.7 (7.1 , 12.7)	1.6 (1.2 , 2.1)	-1.59 (-1.75 , -1.43)
India	6815.7 (5532.3 , 8305.1)	1.5 (1.2 , 1.8)	13222.7 (10367.7 , 16310.8)	1.1 (0.9 , 1.4)	-1.17 (-1.28 , -1.05)
Indonesia	401.1 (302.7 , 502.3)	0.4 (0.3 , 0.6)	1014.6 (754.9 , 1317.1)	0.5 (0.3 , 0.6)	0.13 (0.01 , 0.25)
Iran (Islamic Republic of)	317.3 (228.4 , 416.3)	1.3 (1 , 1.7)	756.6 (561 , 956)	1.1 (0.8 , 1.3)	-0.59 (-0.75 , -0.43)
Iraq	40.3 (29 , 55.3)	0.5 (0.4 , 0.7)	107.3 (68.7 , 154.3)	0.5 (0.3 , 0.7)	-0.65 (-0.85 , -0.44)
Ireland	175.7 (141 , 212)	4.2 (3.4 , 5.1)	155.1 (114.2 , 201.7)	1.9 (1.4 , 2.5)	-2.53 (-2.67 , -2.38)
Israel	41.8 (32.5 , 52.7)	0.9 (0.7 , 1.1)	59.5 (44.1 , 77.8)	0.5 (0.4 , 0.6)	-2.17 (-2.34 , -2)
Italy	1364.6 (1076.8 , 1662.9)	1.5 (1.2 , 1.9)	840.8 (638 , 1069.8)	0.6 (0.4 , 0.7)	-3.18 (-3.24 , -3.13)
Jamaica	14.7 (10.8 , 19.1)	0.8 (0.6 , 1.1)	21.8 (14.6 , 31.8)	0.7 (0.5 , 1)	-0.7 (-1.2 , -0.2)
Japan	4629.4 (3852.1 , 5356.4)	2.7 (2.2 , 3.1)	5935.3 (4586 , 7310.1)	1.6 (1.2 , 1.9)	-1.99 (-2.16 , -1.82)
Jordan	6.4 (4.7 , 8.3)	0.5 (0.4 , 0.7)	27.6 (19.8 , 36.1)	0.4 (0.3 , 0.5)	-0.89 (-1.06 , -0.73)
Kazakhstan	666.7 (492.9 , 862.5)	5.2 (3.8 , 6.8)	208.4 (158.7 , 267.2)	1.2 (0.9 , 1.5)	-4.8 (-5.1 , -4.51)
Kenya	100.4 (71.1 , 147.4)	1.3 (0.9 , 1.9)	295.1 (209.1 , 425.2)	1.3 (0.9 , 1.9)	0.15 (-0.05 , 0.35)
Kiribati	0.9 (0.6 , 1.2)	2.4 (1.7 , 3.2)	1.6 (1.1 , 2.2)	2.2 (1.6 , 3.1)	-0.47 (-0.65 , -0.28)
Kuwait	3.4 (2.4 , 4.4)	0.6 (0.4 , 0.8)	9 (6.3 , 11.8)	0.4 (0.3 , 0.5)	-2.02 (-2.88 , -1.16)
Kyrgyzstan	104.4 (82.5 , 128.6)	3.5 (2.8 , 4.2)	57.8 (44.6 , 73.9)	1.3 (1 , 1.6)	-2.92 (-3.08 , -2.77)
Lao People's Democratic Republic	35.5 (24 , 49.8)	1.8 (1.2 , 2.4)	42.4 (30.6 , 59.1)	1 (0.7 , 1.4)	-1.92 (-2.01 , -1.83)
Latvia	46.8 (38.3 , 56.9)	1.3 (1.1 , 1.6)	40.7 (30.2 , 50.6)	1.1 (0.8 , 1.4)	-0.33 (-0.54 , -0.12)
Lebanon	13 (9.3 , 18)	0.6 (0.4 , 0.9)	30.4 (22.2 , 40.1)	0.5 (0.4 , 0.7)	-0.27 (-0.47 , -0.06)
Lesotho	26.6 (16.9 , 39.5)	3.3 (2.1 , 4.9)	53.7 (37.7 , 74.1)	5.1 (3.7 , 7.1)	2.07 (1.73 , 2.4)
Liberia	3.4 (2.5 , 4.6)	0.3 (0.2 , 0.4)	11.3 (7.3 , 16.6)	0.5 (0.3 , 0.8)	2.38 (2.05 , 2.7)
Libya	12.1 (7.8 , 18.1)	0.7 (0.4 , 1)	40.3 (26.5 , 57.3)	0.8 (0.5 , 1.1)	1.02 (0.84 , 1.2)
Lithuania	56.3 (43.3 , 69.9)	1.2 (1 , 1.5)	69.4 (52.8 , 86.1)	1.3 (1 , 1.6)	0.28 (0.09 , 0.48)
Luxembourg	11.9 (8.5 , 15.7)	2.2 (1.6 , 2.9)	13.6 (9.4 , 18.9)	1.3 (0.9 , 1.8)	-1.81 (-1.99 , -1.62)
Madagascar	168.3 (117.3 , 233.6)	3.2 (2.3 , 4.5)	225.2 (142.3 , 334.4)	1.8 (1.1 , 2.6)	-1.98 (-2.07 , -1.89)
Malawi	123.9 (90.5 , 160.7)	3.5 (2.6 , 4.5)	321.3 (226.8 , 435)	4.5 (3.2 , 6)	0.83 (0.37 , 1.28)
Malaysia	78.6 (60 , 99.7)	0.9 (0.7 , 1.2)	216.3 (164.1 , 277.1)	0.8 (0.6 , 1)	-0.74 (-0.92 , -0.56)

Maldives	1.1 (0.8 , 1.5)	1.3 (0.9 , 1.8)	1.6 (1.2 , 2.1)	0.5 (0.4 , 0.7)	-3.39 (-3.62 , -3.16)
Mali	10.2 (7.5 , 13.3)	0.3 (0.2 , 0.3)	29 (19.3 , 41.5)	0.3 (0.2 , 0.5)	1.36 (1.17 , 1.55)
Malta	5.9 (4.5 , 7.4)	1.4 (1.1 , 1.7)	7.8 (5.7 , 10.3)	0.8 (0.6 , 1.1)	-1.56 (-1.68 , -1.44)
Marshall Islands	0.1 (0.1 , 0.2)	0.7 (0.4 , 1.1)	0.2 (0.1 , 0.3)	0.6 (0.4 , 0.9)	-0.06 (-0.18 , 0.07)
Mauritania	4.6 (3.1 , 6.5)	0.5 (0.3 , 0.7)	14 (8.6 , 21.2)	0.6 (0.4 , 1)	1.51 (1.14 , 1.87)
Mauritius	7.4 (6 , 8.8)	1 (0.8 , 1.3)	19 (15.4 , 22.6)	1 (0.8 , 1.2)	-0.82 (-1.3 , -0.33)
Mexico	231.6 (179.1 , 286.7)	0.6 (0.5 , 0.8)	262.6 (194.6 , 334.7)	0.2 (0.2 , 0.3)	-3.54 (-3.63 , -3.45)
Micronesia (Federated States of)	0.5 (0.3 , 0.6)	0.9 (0.6 , 1.3)	0.6 (0.4 , 0.9)	0.8 (0.5 , 1.1)	-0.68 (-0.72 , -0.65)
Monaco	1.4 (0.9 , 2)	2 (1.3 , 2.9)	1.9 (1.2 , 2.8)	1.9 (1.2 , 2.9)	-0.17 (-0.39 , 0.06)
Mongolia	48 (35 , 63.5)	4.8 (3.5 , 6.4)	83.3 (58.2 , 111.4)	4 (2.7 , 5.2)	-0.74 (-0.87 , -0.6)
Montenegro	7.2 (5.1 , 9.6)	1.1 (0.8 , 1.5)	11.9 (8.2 , 16.2)	1.2 (0.8 , 1.6)	0.39 (0.26 , 0.51)
Morocco	23.7 (16.2 , 32.6)	0.2 (0.1 , 0.2)	51 (33.2 , 70.8)	0.1 (0.1 , 0.2)	-0.45 (-0.69 , -0.21)
Mozambique	72.5 (52.3 , 96.1)	1.3 (1 , 1.8)	146.3 (102.1 , 195.3)	1.4 (1 , 1.9)	0.72 (0.51 , 0.93)
Myanmar	422.5 (302.8 , 571.6)	1.8 (1.3 , 2.5)	380 (270 , 530.4)	0.8 (0.6 , 1.1)	-3.12 (-3.27 , -2.98)
Namibia	3 (2.2 , 4)	0.5 (0.4 , 0.7)	5.3 (3.8 , 7)	0.4 (0.3 , 0.5)	-0.96 (-1.32 , -0.59)
Nauru	0.1 (0 , 0.1)	1.1 (0.7 , 1.6)	0 (0 , 0.1)	0.8 (0.5 , 1.1)	-1.39 (-1.56 , -1.22)
Nepal	225.2 (162.7 , 303.3)	2.5 (1.8 , 3.4)	393.3 (271.2 , 549.8)	1.8 (1.2 , 2.5)	-1.12 (-1.46 , -0.78)
Netherlands	539.4 (427.9 , 644.5)	2.7 (2.1 , 3.2)	887.1 (660.8 , 1114.5)	2.4 (1.8 , 3)	-0.35 (-0.62 , -0.07)
New Zealand	69.9 (52.9 , 86.8)	1.8 (1.3 , 2.2)	75.3 (55.8 , 98.1)	0.9 (0.6 , 1.1)	-2.47 (-2.67 , -2.28)
Nicaragua	3.2 (2.3 , 4.2)	0.2 (0.2 , 0.3)	7.6 (5.2 , 10.7)	0.2 (0.1 , 0.2)	-0.83 (-1.04 , -0.61)
Niger	7.4 (4.9 , 10.3)	0.3 (0.2 , 0.4)	31.4 (21.1 , 46.4)	0.4 (0.3 , 0.6)	1.88 (1.65 , 2.12)
Nigeria	77.8 (54.4 , 113.2)	0.2 (0.1 , 0.3)	202.9 (136.6 , 286.3)	0.2 (0.2 , 0.3)	1.18 (0.95 , 1.41)
Niue	0 (0 , 0)	0.6 (0.4 , 0.9)	0 (0 , 0)	0.6 (0.4 , 0.9)	-0.32 (-0.45 , -0.19)
North Macedonia	14 (10 , 18.1)	0.7 (0.5 , 0.9)	23.3 (16.1 , 31.3)	0.7 (0.5 , 0.9)	-0.22 (-0.54 , 0.09)
Northern Mariana Islands	0.1 (0 , 0.1)	0.4 (0.3 , 0.6)	0.3 (0.2 , 0.4)	0.6 (0.4 , 0.9)	2.68 (2.09 , 3.28)
Norway	73.4 (58.9 , 89.1)	1.1 (0.9 , 1.3)	80.5 (58.3 , 103.1)	0.8 (0.6 , 1)	-1.45 (-1.7 , -1.21)
Oman	2.7 (1.7 , 4)	0.4 (0.3 , 0.6)	5.5 (3.7 , 8)	0.3 (0.2 , 0.5)	-0.4 (-0.63 , -0.16)
Pakistan	1303.9 (1018.2 , 1583.5)	2.4 (1.9 , 3)	2311.2 (1734.8 , 3052.3)	2 (1.5 , 2.7)	-0.98 (-1.25 , -0.71)
Palau	0.1 (0.1 , 0.1)	0.9 (0.6 , 1.2)	0.2 (0.1 , 0.2)	0.7 (0.5 , 1)	-0.67 (-0.74 , -0.6)
Palestine	4.7 (3.2 , 6.8)	0.6 (0.4 , 0.9)	8.3 (5.7 , 11.5)	0.4 (0.3 , 0.5)	-1.89 (-2.15 , -1.63)
Panama	6.9 (5.1 , 8.9)	0.5 (0.4 , 0.6)	9.6 (6.6 , 13.8)	0.2 (0.1 , 0.3)	-2.74 (-2.87 , -2.62)
Papua New Guinea	7.3 (4.5 , 11.4)	0.4 (0.2 , 0.6)	15.1 (10 , 23.1)	0.3 (0.2 , 0.4)	-1.31 (-1.43 , -1.19)
Paraguay	32.6 (23.8 , 42.2)	1.5 (1.1 , 2)	79.9 (53.7 , 115.1)	1.4 (0.9 , 2)	-0.48 (-0.67 , -0.29)
Peru	22.1 (15.4 , 31.2)	0.2 (0.1 , 0.3)	47 (31 , 70.5)	0.1 (0.1 , 0.2)	-1.56 (-1.89 , -1.23)
Philippines	164.6 (130.2 , 208.7)	0.6 (0.5 , 0.7)	372.7 (280.4 , 475.5)	0.5 (0.3 , 0.6)	-0.82 (-0.99 , -0.65)
Poland	770.1 (630.8 , 909.6)	1.8 (1.4 , 2.1)	830.7 (647 , 1027.8)	1.2 (0.9 , 1.5)	-1.5 (-1.69 , -1.32)
Portugal	237 (177 , 301.6)	1.7 (1.3 , 2.2)	204.3 (152.7 , 260.4)	0.9 (0.7 , 1.2)	-1.78 (-1.95 , -1.6)
Puerto Rico	60.4 (39 , 90.1)	1.7 (1.1 , 2.5)	38.1 (24 , 59)	0.5 (0.3 , 0.8)	-3.71 (-3.91 , -3.51)
Qatar	1.3 (0.8 , 1.9)	1.8 (1.1 , 2.5)	4.8 (2.8 , 7.2)	0.7 (0.4 , 1.1)	-3.2 (-3.93 , -2.47)
Republic of Korea	1019.4 (765.7 , 1291.9)	3.5 (2.6 , 4.4)	1137.5 (810.2 , 1522.4)	1.2 (0.8 , 1.6)	-4.05 (-4.25 , -3.85)
Republic of Moldova	54.7 (40.8 , 68.7)	1.2 (0.9 , 1.5)	60.7 (47.5 , 75.1)	1 (0.8 , 1.2)	-0.41 (-0.86 , 0.04)
Romania	162.8 (126.9 , 202.2)	0.6 (0.4 , 0.7)	318.4 (244.5 , 402.5)	0.9 (0.7 , 1.2)	1.12 (0.79 , 1.45)

Russian Federation	3448.9 (2832.6 , 4026.7)	1.8 (1.5 , 2.2)	3147.3 (2526.4 , 3809.9)	1.3 (1 , 1.6)	-1.32 (-1.46 , -1.18)
Rwanda	128.9 (83.9 , 176.7)	4.8 (3.1 , 6.5)	165.2 (112.7 , 232.1)	2.9 (2 , 4.2)	-2.5 (-2.87 , -2.13)
Saint Kitts and Nevis	0.3 (0.2 , 0.4)	0.8 (0.5 , 1.1)	0.4 (0.3 , 0.5)	0.6 (0.4 , 0.8)	-0.66 (-0.78 , -0.54)
Saint Lucia	1.1 (0.8 , 1.4)	1.3 (0.9 , 1.6)	2.1 (1.4 , 2.8)	0.8 (0.6 , 1.1)	-1.38 (-1.58 , -1.17)
Saint Vincent and the Grenadines	0.3 (0.2 , 0.4)	0.4 (0.3 , 0.5)	0.6 (0.4 , 0.8)	0.4 (0.3 , 0.6)	0.23 (-0.06 , 0.52)
Samoa	0.3 (0.2 , 0.4)	0.4 (0.3 , 0.5)	0.5 (0.3 , 0.6)	0.3 (0.2 , 0.4)	-1.01 (-1.12 , -0.89)
San Marino	0.3 (0.2 , 0.4)	0.8 (0.5 , 1.1)	0.3 (0.2 , 0.4)	0.3 (0.2 , 0.6)	-1.75 (-2.08 , -1.41)
Sao Tome and Principe	0.1 (0.1 , 0.1)	0.2 (0.1 , 0.2)	0.5 (0.3 , 0.7)	0.4 (0.3 , 0.6)	3.5 (3.29 , 3.71)
Saudi Arabia	27.6 (16.6 , 43.2)	0.5 (0.3 , 0.8)	78.6 (49.4 , 113.1)	0.5 (0.3 , 0.7)	-0.65 (-0.8 , -0.5)
Senegal	13.7 (9.8 , 19)	0.4 (0.3 , 0.6)	44 (30 , 64)	0.6 (0.4 , 0.8)	1.37 (1.06 , 1.68)
Serbia	112.6 (75.7 , 161.6)	1 (0.7 , 1.4)	144.5 (95 , 211.9)	0.9 (0.6 , 1.3)	-0.3 (-0.51 , -0.08)
Seychelles	1.2 (0.9 , 1.6)	2.2 (1.6 , 2.9)	2.1 (1.6 , 2.7)	1.8 (1.4 , 2.3)	-0.62 (-0.8 , -0.43)
Sierra Leone	9 (6.5 , 12.3)	0.4 (0.3 , 0.6)	24 (16.9 , 33.9)	0.6 (0.4 , 0.9)	1.91 (1.61 , 2.22)
Singapore	34.8 (25 , 45.2)	1.6 (1.2 , 2.1)	37 (26.7 , 48.2)	0.4 (0.3 , 0.6)	-4.35 (-4.69 , -4)
Slovakia	100.5 (69.1 , 138.8)	1.7 (1.2 , 2.3)	105.4 (72.1 , 149)	1.1 (0.8 , 1.6)	-1.3 (-1.4 , -1.2)
Slovenia	40.5 (30.9 , 50)	1.6 (1.2 , 2)	40.4 (28.7 , 52.2)	1 (0.7 , 1.2)	-1.88 (-2.04 , -1.71)
Solomon Islands	1.2 (0.8 , 1.7)	0.9 (0.6 , 1.3)	2.6 (1.6 , 3.8)	0.7 (0.5 , 1.1)	-0.51 (-0.62 , -0.4)
Somalia	62.5 (37.5 , 94.4)	2.6 (1.6 , 3.8)	92.5 (60.7 , 140.1)	1.5 (1 , 2.2)	-1.95 (-2.07 , -1.84)
South Africa	741 (577.3 , 927.6)	3.7 (2.8 , 4.6)	949.4 (745.6 , 1174.5)	2.1 (1.6 , 2.5)	-2.55 (-3.04 , -2.06)
South Sudan	66.8 (44.8 , 97.5)	2.6 (1.8 , 3.8)	68.1 (43.4 , 101.1)	1.8 (1.2 , 2.6)	-1.58 (-1.79 , -1.37)
Spain	1208.6 (991.1 , 1431.6)	2.3 (1.8 , 2.7)	942.8 (715.1 , 1149.6)	1 (0.8 , 1.2)	-2.6 (-2.73 , -2.46)
Sri Lanka	197.6 (152.2 , 253.3)	2 (1.5 , 2.5)	317.4 (188.2 , 449.9)	1.2 (0.7 , 1.7)	-1.23 (-1.49 , -0.97)
Sudan	122 (79.8 , 174.4)	1.4 (0.9 , 2)	254.2 (154.7 , 384.2)	1.5 (0.9 , 2.2)	0.09 (0.04 , 0.14)
Suriname	1.3 (1 , 1.8)	0.5 (0.4 , 0.7)	2.8 (1.8 , 4)	0.4 (0.3 , 0.6)	-0.56 (-0.79 , -0.33)
Sweden	185.4 (145.9 , 228.7)	1.2 (0.9 , 1.4)	241.8 (176.3 , 314.9)	1.1 (0.8 , 1.4)	-0.27 (-0.43 , -0.12)
Switzerland	236.4 (186.5 , 286.3)	2.3 (1.8 , 2.8)	229.1 (175.5 , 287.4)	1.2 (0.9 , 1.5)	-1.69 (-1.86 , -1.51)
Syrian Arab Republic	16.3 (11.5 , 22)	0.3 (0.2 , 0.5)	38.5 (25.1 , 54.5)	0.3 (0.2 , 0.5)	-0.25 (-0.42 , -0.08)
Taiwan (Province of China)	774.6 (630.7 , 916.6)	4.9 (4 , 5.8)	1383.5 (1096.3 , 1661.6)	3.3 (2.6 , 3.9)	-1.38 (-1.67 , -1.09)
Tajikistan	129.4 (91.7 , 168.7)	4.8 (3.5 , 6.3)	105.3 (63.7 , 164.8)	2.1 (1.3 , 3.3)	-2.5 (-2.79 , -2.21)
Thailand	742.8 (530.1 , 1060.5)	2.2 (1.6 , 3.1)	1554.4 (1130.9 , 2182.9)	1.4 (1 , 2)	-1.82 (-1.96 , -1.68)
Timor-Leste	1.9 (1.2 , 2.8)	0.8 (0.5 , 1.1)	4.8 (3.2 , 6.8)	0.6 (0.4 , 0.8)	-0.8 (-1 , -0.59)
Togo	8.4 (5.6 , 12.5)	0.7 (0.5 , 1.1)	43.3 (28.1 , 63.3)	1.1 (0.8 , 1.7)	1.92 (1.72 , 2.12)
Tokelau	0 (0 , 0)	0.6 (0.4 , 0.9)	0 (0 , 0)	0.5 (0.3 , 0.7)	-1.11 (-1.21 , -1.02)
Tonga	0.4 (0.3 , 0.6)	0.8 (0.6 , 1.1)	0.6 (0.4 , 0.8)	0.8 (0.5 , 1.1)	-0.6 (-0.85 , -0.35)
Trinidad and Tobago	5.1 (3.9 , 6.5)	0.6 (0.5 , 0.8)	8.1 (5.3 , 11.4)	0.4 (0.3 , 0.6)	-1.25 (-1.47 , -1.04)
Tunisia	14.5 (10.6 , 19.1)	0.3 (0.2 , 0.4)	38.5 (24 , 56.6)	0.3 (0.2 , 0.4)	-0.28 (-0.35 , -0.2)
Turkey	339.5 (245.3 , 449.2)	1 (0.7 , 1.3)	570.1 (389.2 , 764.6)	0.6 (0.4 , 0.8)	-1.84 (-2.08 , -1.6)
Turkmenistan	197.7 (161.9 , 234)	10.4 (8.6 , 12.3)	89.9 (62 , 123.5)	2.3 (1.6 , 3.2)	-4.89 (-5.37 , -4.41)
Tuvalu	0.1 (0 , 0.1)	0.7 (0.5 , 1)	0.1 (0 , 0.1)	0.6 (0.4 , 0.8)	-0.82 (-0.88 , -0.76)
Uganda	98.2 (69.5 , 131.1)	1.6 (1.1 , 2.1)	204.2 (145.8 , 281.3)	1.4 (1 , 2)	-0.98 (-1.37 , -0.6)
Ukraine	1073.3 (872.7 , 1278.3)	1.5 (1.2 , 1.7)	685.1 (431.3 , 1013.3)	0.9 (0.6 , 1.3)	-1.9 (-2.05 , -1.75)

United Arab Emirates	2.5 (1.6 , 3.8)	0.7 (0.4 , 1.1)	11.9 (7.8 , 16.9)	0.6 (0.4 , 0.8)	0.66 (0.05 , 1.28)
United Kingdom	3511.6 (2772.4 , 4143)	3.8 (3 , 4.5)	3772.7 (2809.3 , 4721)	2.7 (2 , 3.4)	-1.22 (-1.43 , -1)
United Republic of Tanzania	298.2 (217.5 , 402.8)	2.8 (2.1 , 3.8)	371.2 (253.8 , 526.2)	1.5 (1 , 2.1)	-2.55 (-2.75 , -2.36)
United States of America	6956.3 (5663.1 , 8150.2)	2.2 (1.8 , 2.6)	10453.4 (8063.1 , 12806.5)	1.7 (1.4 , 2.1)	-0.9 (-1.09 , -0.71)
United States Virgin Islands	0.7 (0.4 , 1.1)	0.8 (0.5 , 1.3)	0.9 (0.5 , 1.5)	0.5 (0.3 , 0.8)	-1.8 (-2 , -1.59)
Uruguay	117.4 (89.7 , 146)	3 (2.3 , 3.7)	96.3 (72 , 121.5)	1.7 (1.3 , 2.2)	-2.04 (-2.31 , -1.78)
Uzbekistan	252.2 (186.9 , 322.7)	2.2 (1.6 , 2.8)	176 (131.2 , 227.8)	0.7 (0.5 , 0.9)	-4.05 (-4.66 , -3.44)
Vanuatu	0.4 (0.2 , 0.5)	0.6 (0.4 , 0.8)	0.7 (0.5 , 1)	0.4 (0.3 , 0.6)	-1.34 (-1.45 , -1.23)
Venezuela (Bolivarian Republic of)	62.1 (48 , 78.3)	0.7 (0.5 , 0.9)	88.9 (53.9 , 125.4)	0.3 (0.2 , 0.4)	-2.82 (-2.98 , -2.65)
Viet Nam	349.1 (244.9 , 480)	0.9 (0.6 , 1.2)	945.5 (666.8 , 1259.1)	0.9 (0.7 , 1.2)	0.23 (0.18 , 0.27)
Yemen	107.5 (72.5 , 152.6)	2.3 (1.5 , 3.2)	225.5 (142.4 , 321.5)	1.8 (1.1 , 2.5)	-1.16 (-1.32 , -1.01)
Zambia	73.3 (52.2 , 99)	2.7 (1.9 , 3.7)	125.9 (84.7 , 177.4)	2 (1.4 , 2.8)	-1.61 (-1.9 , -1.32)
Zimbabwe	153.6 (105 , 209.9)	4 (2.7 , 5.5)	259.9 (181.5 , 346.4)	4 (2.9 , 5.3)	-0.05 (-0.39 , 0.3)

sTable 2 Tobacco-Related Esophageal Cancer Disability-Adjusted Life Years (DALYs) Between 1990 and 2021 for Both Sexes, Each SDI Regions and All countries, With EAPC Between 1990 and 2021.

	1990 DALYs		2021 DALYs		EAPC% (95% CI) 1990–2021
	Case number (95% UI)	ASR/100,000 persons (95% UI)	Case number (95% UI)	ASR/100,000 persons (95% UI)	
Global	3844095.6 (3139093.9 , 4585376.4)	93.3 (76.2 , 111.3)	5136277 (4040644.3 , 6350151.2)	58.5 (46 , 72.3)	-1.69 (-1.8 , -1.58)
**Sex**					
Male	3500527.9 (2831848.8 , 4207047)	179.7 (145.8 , 215.2)	4754997.4 (3709201.3 , 5904764.1)	114.8 (89.5 , 142.6)	-1.62 (-1.72 , -1.51)
Female	343567.7 (259972.5 , 426105.1)	16.1 (12.2 , 20)	381279.6 (292975 , 487652.5)	8.3 (6.3 , 10.6)	-2.46 (-2.61 , -2.31)
**SDI**					
High SDI	676154.4 (558730.9 , 787082.5)	62.7 (51.9 , 73)	763421.5 (608706.6 , 920289.8)	38.5 (30.9 , 46.3)	-1.71 (-1.85 , -1.56)
High-middle SDI	1262909.4 (1002474.6 , 1542666.1)	122.6 (97.5 , 149.8)	1763633.7 (1324867.1 , 2308908.3)	87.6 (65.8 , 114.5)	-1.24 (-1.38 , -1.11)
Middle SDI	1556511.5 (1242518 , 1932994.1)	140.7 (112.5 , 174.5)	2037140.5 (1540952.1 , 2630987.7)	73.4 (55.4 , 94.9)	-2.31 (-2.42 , -2.21)
Low-middle SDI	267895.1 (224058.1 , 319292.7)	40.9 (34 , 48.7)	443711 (362461.1 , 531665.3)	29.4 (24 , 35.1)	-1.2 (-1.28 , -1.13)
Low SDI	79010.8 (64043.5 , 94590.9)	32.4 (26.2 , 38.7)	126444.4 (100439.6 , 155506.9)	23.2 (18.5 , 28.5)	-1.31 (-1.41 , -1.22)
**Country**					
Afghanistan	2030.6 (1161.4 , 3192.1)	27.8 (16.2 , 43.8)	2902.2 (1772 , 4523.3)	25.5 (15.6 , 38.6)	-0.43 (-0.78 , -0.08)
Albania	483.3 (356.9 , 628.8)	22.8 (16.9 , 29.4)	777.2 (522.5 , 1075.5)	17.9 (12.1 , 24.7)	-0.41 (-0.66 , -0.17)
Algeria	793.4 (593 , 1028)	6.7 (5 , 8.6)	2035.5 (1380 , 2812)	5.8 (4 , 8)	-0.49 (-0.55 , -0.43)
American Samoa	2.3 (1.6 , 3.2)	9.2 (6.4 , 12.6)	5.7 (4 , 7.8)	11.1 (7.7 , 14.9)	1.04 (0.71 , 1.37)
Andorra	13.9 (8.4 , 21.4)	23.7 (14.4 , 36.4)	17.1 (9.1 , 27)	11.2 (5.9 , 17.5)	-2.19 (-2.44 , -1.93)
Angola	2512.5 (1603.8 , 3670.3)	56.8 (36.5 , 82.1)	4763.3 (3264.7 , 6762.2)	35.3 (24.6 , 49.7)	-1.66 (-1.94 , -1.37)
Antigua and Barbuda	8.3 (5.9 , 11)	16.3 (11.7 , 21.6)	13.9 (9.9 , 18.2)	12.5 (8.9 , 16.5)	-0.73 (-1.01 , -0.45)
Argentina	20331.2 (15717.6 , 24684.9)	61.9 (48 , 75.4)	15533.5 (11637.5 , 19551.5)	28.4 (21.3 , 35.5)	-2.45 (-2.73 , -2.17)
Armenia	955.8 (806.1 , 1096.6)	33 (27.8 , 37.7)	524.9 (427.1 , 629.9)	11.6 (9.4 , 13.9)	-3.26 (-3.64 , -2.88)
Australia	7347.4 (5725.2 , 9100.5)	37.4 (29.1 , 46.3)	8207.2 (5932 , 10692.2)	19.2 (14.1 , 24.8)	-2.2 (-2.29 , -2.11)
Austria	3075.6 (2418.8 , 3751.6)	28.6 (22.4 , 34.8)	3885.1 (3086 , 4786.6)	23.6 (18.8 , 29.1)	-0.4 (-0.56 , -0.24)
Azerbaijan	4006.1 (2919.5 , 5123)	75.3 (54.7 , 96.3)	5714.7 (3965.8 , 7699.5)	52.9 (36.6 , 71.5)	-0.9 (-1.12 , -0.68)
Bahamas	60.1 (39.3 , 86.4)	37.7 (24.5 , 54.7)	121.5 (80.6 , 166.7)	27.8 (18.4 , 38.5)	-0.41 (-0.7 , -0.12)
Bahrain	59.1 (40.3 , 80.8)	36.4 (25.2 , 49.1)	139.9 (91 , 207.9)	17.1 (11.7 , 24.5)	-3.32 (-3.66 , -2.98)
Bangladesh	36569.7 (25041.9 , 51775.1)	74.1 (50.2 , 104.1)	58118.1 (38558 , 83419)	40.9 (27.2 , 58.5)	-1.81 (-1.94 , -1.68)
Barbados	80.8 (59.1 , 106.8)	28.6 (20.9 , 37.6)	93 (60.4 , 130)	18 (11.8 , 25.3)	-1.68 (-1.87 , -1.49)
Belarus	5214.2 (4202.5 , 6313.7)	39.3 (31.8 , 47.5)	6259.3 (4524.6 , 8053.8)	39.2 (28.4 , 50.2)	-0.44 (-0.62 , -0.27)
Belgium	7302.7 (5875 , 8665.9)	50.2 (40.7 , 59.5)	8634.1 (6637.8 , 10704.6)	40.4 (31.2 , 49.8)	-0.76 (-1.03 , -0.48)
Belize	10.1 (7.7 , 12.8)	10.9 (8.3 , 13.9)	33.9 (25.1 , 44.4)	10.6 (7.8 , 13.9)	0.32 (-0.15 , 0.79)
Benin	199.4 (145.3 , 273.4)	9.9 (7.2 , 13.5)	624.3 (427.6 , 860.9)	11.6 (8 , 15.8)	0.87 (0.7 , 1.03)
Bermuda	24.3 (16.8 , 33.6)	38.3 (26.5 , 53)	26.9 (18.8 , 36.4)	21 (14.5 , 28.1)	-1.53 (-1.83 , -1.22)
Bhutan	130.7 (85.5 , 185.5)	50.3 (32.9 , 71.2)	198.4 (134 , 285.2)	32.5 (22 , 46.6)	-1.48 (-1.69 , -1.27)
Bolivia (Plurinational State of)	347.1 (240.1 , 503.6)	10.7 (7.3 , 15.5)	655.6 (417.7 , 989.2)	7.1 (4.5 , 10.7)	-0.99 (-1.23 , -0.75)
Bosnia and Herzegovina	1257.2 (955 , 1617.5)	27.9 (21.1 , 35.7)	1669.8 (1144 , 2281.3)	27.5 (18.8 , 37.6)	0.12 (-0.08 , 0.31)
Botswana	556.1 (373.8 , 798.5)	92.3 (62 , 131.3)	934.8 (648.1 , 1309.8)	59.2 (41.3 , 82.8)	-1.8 (-2.12 , -1.48)

Brazil	77032.5 (62248.1 , 91596)	82.5 (66.4 , 98.6)	86633.6 (65959.4 , 109005.2)	33.7 (25.7 , 42.4)	-3.09 (-3.22 , -2.96)
Brunei Darussalam	37.5 (27 , 50.5)	38.1 (27.2 , 50.6)	67.5 (46.9 , 93.4)	18.3 (12.8 , 25.1)	-2.03 (-2.25 , -1.81)
Bulgaria	4325.2 (3459.7 , 5207.2)	34.3 (27.7 , 41.2)	3601.2 (2745 , 4529.3)	29.3 (22.5 , 36.7)	-0.74 (-0.96 , -0.51)
Burkina Faso	465.6 (332.8 , 632.8)	9.9 (7.1 , 13.6)	1685.1 (1108.3 , 2396)	16.5 (10.8 , 23.5)	2.27 (2.04 , 2.5)
Burundi	2209.5 (1533.1 , 2969.7)	89.6 (62.1 , 120.4)	1846.4 (1275.3 , 2548.9)	32.9 (22.9 , 45)	-3.69 (-3.93 , -3.44)
Cabo Verde	71.1 (52.8 , 92.9)	33 (24.7 , 43.5)	246.2 (170 , 334.4)	52.2 (36.7 , 71.7)	0.74 (0.31 , 1.17)
Cambodia	2641 (1953.6 , 3365.9)	56 (42 , 71.7)	4502.6 (3164 , 6011.7)	34.9 (24.9 , 46.1)	-1.82 (-1.94 , -1.71)
Cameroon	606.9 (402.2 , 850.7)	12.3 (8.3 , 17.1)	2820.6 (1742.5 , 4277.4)	19.8 (12.4 , 29.7)	1.99 (1.74 , 2.24)
Canada	15296.8 (12350.4 , 18255)	47.4 (38.4 , 56.6)	22312.1 (16982 , 28102.7)	31.7 (24.4 , 40.1)	-1.31 (-1.45 , -1.17)
Central African Republic	643.9 (425.9 , 910.7)	48.5 (32.8 , 68.2)	800.5 (522 , 1181.4)	29.7 (19.9 , 42.7)	-1.82 (-1.98 , -1.66)
Chad	253.4 (177.1 , 340.3)	8.8 (6.2 , 11.8)	1112.2 (715.3 , 1687.9)	18.1 (11.9 , 26.9)	2.73 (2.5 , 2.97)
Chile	4176.2 (3187.9 , 5233.9)	40.6 (30.8 , 50.8)	2719.3 (2075.4 , 3450.1)	10.7 (8.2 , 13.6)	-4.64 (-4.87 , -4.41)
China	2324652.1 (1803868.8 , 2921620.2)	260.8 (202.2 , 326.7)	3238100.2 (2353463 , 4262162.7)	147.1 (107.3 , 192.8)	-2.04 (-2.19 , -1.9)
Colombia	3695.2 (2888.3 , 4553.7)	20.5 (15.8 , 25.5)	2511.4 (1794.4 , 3373.5)	4.5 (3.2 , 6.1)	-5.45 (-5.69 , -5.21)
Comoros	235.3 (160.9 , 338.9)	109.7 (75.5 , 156.7)	361.7 (243.5 , 533.1)	69.2 (47 , 101.7)	-1.91 (-2.08 , -1.74)
Congo	603.6 (394.6 , 903)	51.9 (34.3 , 77.2)	1116.1 (728.8 , 1671.5)	37.2 (24.4 , 53.2)	-1.24 (-1.49 , -1)
Cook Islands	2.7 (1.8 , 3.8)	20.9 (13.9 , 29.2)	3.9 (2.7 , 5.5)	15.1 (10.5 , 21.4)	-1.12 (-1.22 , -1.02)
Costa Rica	314.1 (237.1 , 394.3)	18.2 (13.8 , 23)	409.7 (295.4 , 536)	7.4 (5.4 , 9.7)	-3.19 (-3.38 , -3.01)
Coted'Ivoire	202.7 (142.5 , 276.2)	4.5 (3.2 , 6.1)	546.2 (362.6 , 778.8)	4.3 (2.9 , 6.2)	-0.46 (-0.82 , -0.11)
Croatia	3550.1 (2863.8 , 4205.8)	54.9 (44.2 , 65)	2548.4 (1946.3 , 3167.1)	31.5 (24.2 , 39.3)	-1.68 (-1.88 , -1.49)
Cuba	3866.2 (3089.2 , 4670.3)	38.1 (30.5 , 46)	9033 (6752.4 , 11411.8)	46.4 (34.8 , 58.5)	0.99 (0.87 , 1.11)
Cyprus	112.5 (79 , 150.3)	14.3 (10.1 , 19.1)	279.9 (194.2 , 381.9)	13.7 (9.5 , 18.6)	0.56 (0.31 , 0.8)
Czechia	4770.9 (3783.4 , 5843.3)	35.7 (28.3 , 43.7)	6208.6 (4591.4 , 7924.4)	31.7 (23.5 , 40.5)	-0.53 (-0.67 , -0.4)
Democratic People's Republic of Korea	18218.4 (11903.2 , 25792.1)	100.6 (66.1 , 142.1)	30361 (20514.7 , 44462.5)	86.8 (58.8 , 126.4)	-0.51 (-0.63 , -0.38)
Democratic Republic of the Congo	4864 (3162.7 , 6771.1)	27.5 (18.1 , 38.3)	7957 (4914.8 , 11563.7)	18.8 (11.6 , 27.2)	-1.36 (-1.53 , -1.18)
Denmark	5081.8 (4186.2 , 5952.2)	67.2 (55.2 , 78.6)	5272 (4103.3 , 6454)	46.1 (35.9 , 56.2)	-1.46 (-1.62 , -1.3)
Djibouti	128.2 (80.9 , 199.3)	82.7 (51.7 , 124.8)	450.9 (268.6 , 700.7)	63.1 (39 , 96.1)	-0.94 (-1.05 , -0.83)
Dominica	11.2 (8 , 15.1)	19.4 (14 , 26)	15.6 (10.5 , 22.8)	17.8 (11.9 , 26)	-0.05 (-0.31 , 0.2)
Dominican Republic	599.7 (411.7 , 818.4)	16.4 (11.2 , 22.4)	1601.1 (1076.3 , 2304.8)	15.9 (10.6 , 22.9)	0.34 (0.16 , 0.51)
Ecuador	539.5 (419 , 673.7)	10.4 (8.1 , 13)	583.1 (406.5 , 797.1)	3.6 (2.5 , 4.9)	-2.93 (-3.24 , -2.62)
Egypt	2753.8 (2032.3 , 3592.1)	9.6 (7.1 , 12.4)	5884 (4278.4 , 7938.6)	9.2 (6.7 , 12.3)	-0.26 (-0.45 , -0.08)
El Salvador	164.3 (118.1 , 228)	5.5 (3.9 , 7.6)	342.9 (229.4 , 489.9)	5.7 (3.8 , 8.1)	-0.05 (-0.28 , 0.17)
Equatorial Guinea	94.6 (61.7 , 141.2)	42.8 (28.3 , 63.5)	165.1 (98.4 , 243.2)	28.9 (17.7 , 42)	-1.44 (-2.02 , -0.86)
Eritrea	909 (533.2 , 1329.6)	59 (35.3 , 85.9)	1063.2 (608.7 , 1653.3)	30.2 (17.9 , 46.4)	-2.69 (-2.9 , -2.48)
Estonia	787.2 (618.3 , 966.1)	38.3 (30.1 , 46.8)	635.8 (473.2 , 818.5)	27.1 (20.3 , 34.7)	-1.35 (-1.55 , -1.16)
Eswatini	181.5 (123 , 254.6)	59.3 (40.8 , 83)	284.8 (179.7 , 413.2)	46.1 (29.9 , 65.8)	-0.7 (-1.34 , -0.07)
Ethiopia	3492.9 (2369.6 , 5209.9)	15.8 (10.7 , 23.4)	2932.4 (2057.3 , 4086.5)	6.5 (4.6 , 9.2)	-3.01 (-3.32 , -2.69)
Fiji	63 (43.5 , 86.3)	15.8 (11 , 21.6)	111.3 (76.3 , 157.9)	13 (9 , 18.4)	-0.56 (-0.71 , -0.41)
Finland	1766.5 (1346.7 , 2250.2)	26 (20 , 33)	1944.9 (1437.6 , 2517.8)	17.7 (13.4 , 22.5)	-1.11 (-1.21 , -1.02)
France	70191.5 (54849.1 , 83740.5)	93 (73.4 , 110.7)	34625.8 (27025.1 , 43072.4)	28.2 (22.2 , 34.8)	-3.8 (-3.92 , -3.67)
Gabon	198.6 (138.2 , 280.1)	33.2 (23 , 46.8)	375.8 (250.8 , 533.8)	31.7 (21.6 , 44.2)	-0.25 (-0.36 , -0.14)

Gambia	26.4 (17.9 , 35.8)	7 (4.8 , 9.6)	69.1 (45.9 , 95.6)	6.6 (4.4 , 9.1)	-0.34 (-0.49 , -0.19)
Georgia	1490.5 (1153.4 , 1833.5)	22.8 (17.7 , 27.9)	683.3 (525.6 , 848.2)	11.8 (9.1 , 14.6)	-1.04 (-1.59 , -0.49)
Germany	51249 (40369.2 , 61931.6)	43.6 (34.4 , 52.6)	61206.5 (47333.9 , 74916.8)	35.8 (27.9 , 43.6)	-0.92 (-1.12 , -0.72)
Ghana	331.2 (227.3 , 444.5)	5 (3.5 , 6.7)	1187 (820.8 , 1606.4)	6.7 (4.6 , 9.1)	1.61 (1.38 , 1.84)
Greece	4118 (3413.4 , 4841.8)	27 (22.4 , 31.7)	4283.4 (3494.5 , 5068.5)	20.9 (17.2 , 24.7)	-0.95 (-1.1 , -0.8)
Greenland	87.7 (64.8 , 116.8)	233.5 (170.1 , 309)	89.1 (62.8 , 122)	114.6 (80.5 , 160.2)	-2.18 (-2.28 , -2.08)
Grenada	20.6 (14.9 , 28)	32.5 (23.5 , 43.8)	23.8 (17.5 , 30.8)	19 (14 , 24.6)	-1.28 (-1.79 , -0.76)
Guam	12.9 (9.7 , 16.8)	14.5 (10.8 , 19.3)	28.2 (20.6 , 36.7)	13.6 (10 , 17.7)	0.62 (0.26 , 0.97)
Guatemala	255.3 (192.8 , 330.2)	7.4 (5.5 , 9.8)	479.6 (338.9 , 644.4)	4.3 (3.1 , 5.9)	-2.02 (-2.39 , -1.66)
Guinea	127.9 (91.3 , 178.3)	3.7 (2.7 , 5.2)	253.8 (161.3 , 379.1)	4.3 (2.7 , 6.4)	0.7 (0.49 , 0.91)
Guinea-Bissau	41.1 (27.2 , 58.7)	9.5 (6.2 , 13.5)	159.2 (106 , 226)	18.8 (12.7 , 26.4)	3.24 (2.84 , 3.65)
Guyana	38.7 (28.4 , 50.2)	9.6 (7.1 , 12.5)	62.4 (40.7 , 91.5)	8.7 (5.7 , 12.7)	0.17 (-0.02 , 0.36)
Haiti	591.6 (396.8 , 877)	16.3 (10.9 , 24)	688.3 (433.5 , 1043.4)	8.4 (5.3 , 12.8)	-2.04 (-2.24 , -1.85)
Honduras	122.9 (86.4 , 169.5)	6 (4.2 , 8.2)	486.2 (331.8 , 682.1)	7.7 (5.3 , 10.8)	1.2 (1.01 , 1.4)
Hungary	8969.6 (7186.2 , 10702.9)	63.9 (51.3 , 76.4)	6408 (4954.9 , 8109.5)	37.3 (29 , 47)	-2.23 (-2.64 , -1.82)
Iceland	165.9 (131.9 , 198.5)	60.3 (47.9 , 71.8)	207.5 (153.7 , 265)	37.3 (27.7 , 47.3)	-1.71 (-1.87 , -1.55)
India	201502.4 (164016.2 , 245334.9)	39.1 (31.8 , 47.8)	355612.7 (276612.3 , 444121.8)	28.4 (22.1 , 35.3)	-1.34 (-1.45 , -1.22)
Indonesia	11055.6 (8429.1 , 13870)	10.7 (8.1 , 13.4)	27159.3 (19975.3 , 35259.3)	10.6 (7.8 , 13.7)	0.01 (-0.1 , 0.12)
Iran (Islamic Republic of)	8513 (6179.4 , 11178.9)	30.9 (22.3 , 40.5)	17655.3 (13350.9 , 22163.8)	22.6 (17.1 , 28.5)	-0.9 (-1.06 , -0.74)
Iraq	1041.6 (741.1 , 1419.2)	13.1 (9.3 , 17.9)	2722.2 (1720 , 3917.6)	11.2 (7.2 , 16.1)	-0.95 (-1.13 , -0.78)
Ireland	3690 (2955.3 , 4438.3)	90.7 (72.8 , 109.2)	3076.3 (2301.3 , 3986.7)	39.1 (29.3 , 50.6)	-2.61 (-2.75 , -2.47)
Israel	909.6 (722 , 1129.7)	19.1 (15.2 , 23.7)	1238.2 (946.3 , 1574.7)	10.5 (8 , 13.3)	-2.11 (-2.3 , -1.91)
Italy	32245.6 (25741.5 , 38909)	37.6 (30.1 , 45.3)	16960.2 (13112.6 , 21327.7)	13 (10.2 , 16.2)	-3.39 (-3.46 , -3.33)
Jamaica	336.6 (253 , 426.1)	19.5 (14.7 , 24.5)	516.7 (345.8 , 762.7)	16.8 (11.3 , 24.8)	-0.75 (-1.29 , -0.21)
Japan	111069.9 (92742.7 , 128078.4)	63.7 (53.2 , 73.5)	108771 (85520.2 , 133228)	33.5 (26.7 , 40.5)	-2.33 (-2.55 , -2.1)
Jordan	173.2 (129.8 , 227.4)	12.4 (9.3 , 16.1)	715.8 (513.8 , 953.2)	9.2 (6.6 , 12.1)	-1.15 (-1.34 , -0.96)
Kazakhstan	18471.8 (13874.5 , 23421.4)	138 (103.1 , 175.4)	5565.3 (4292.3 , 7085.6)	28.8 (22.1 , 36.7)	-5 (-5.27 , -4.73)
Kenya	2728.9 (1944 , 3956.3)	31.8 (22.6 , 46.2)	8238.6 (5836.5 , 11985.9)	33.1 (23.5 , 47.9)	0.11 (-0.13 , 0.35)
Kiribati	25.1 (18.3 , 32.9)	62.9 (45.2 , 83.3)	46.9 (31.8 , 65.5)	57.7 (39.7 , 80.4)	-0.46 (-0.64 , -0.29)
Kuwait	96.9 (70.3 , 127.4)	14.9 (10.7 , 19.5)	212.5 (151.3 , 279.9)	7.5 (5.3 , 9.9)	-2.62 (-3.44 , -1.78)
Kyrgyzstan	2888 (2286.2 , 3557.5)	92.5 (73.5 , 113.6)	1525.8 (1161.4 , 1968.8)	30.1 (23.1 , 38.7)	-3.43 (-3.57 , -3.28)
Lao People's Democratic Republic	980.1 (655.3 , 1390.7)	44.5 (30 , 62.7)	1113.9 (804 , 1571.6)	23.2 (16.8 , 32.4)	-2.2 (-2.29 , -2.1)
Latvia	1322 (1091.9 , 1603.4)	36.8 (30.4 , 44.5)	1056.1 (796.6 , 1304.2)	31.2 (23.5 , 38.2)	-0.52 (-0.74 , -0.3)
Lebanon	335.6 (239.1 , 463.3)	14.8 (10.6 , 20.4)	637.6 (467.4 , 854.7)	10.8 (7.9 , 14.5)	-0.57 (-0.77 , -0.36)
Lesotho	663.8 (436.1 , 968.9)	77.4 (50.5 , 113.1)	1513.1 (1045.4 , 2104.1)	131.6 (91.4 , 182)	2.36 (1.98 , 2.74)
Liberia	95.1 (68.7 , 127.8)	8 (5.8 , 10.8)	343.4 (223 , 509)	14 (9.1 , 20.7)	2.44 (2.11 , 2.77)
Libya	318.6 (206.1 , 479.1)	16.4 (10.6 , 24.6)	1096.3 (715.4 , 1575.4)	19.5 (12.8 , 27.5)	0.92 (0.76 , 1.08)
Lithuania	1541.5 (1202 , 1887.4)	33.9 (26.5 , 41.5)	1779.6 (1377.8 , 2204.5)	35.4 (27.5 , 43.7)	0.2 (-0.04 , 0.43)
Luxembourg	290.5 (208.8 , 386)	54.9 (39.6 , 73)	302.4 (211.4 , 413.5)	29.4 (20.6 , 40.1)	-2.14 (-2.34 , -1.95)
Madagascar	5109.2 (3552.6 , 7092.4)	90.7 (63.3 , 124.9)	7394.7 (4646.5 , 11010.1)	51.2 (32.5 , 75.6)	-1.9 (-1.97 , -1.82)
Malawi	3398.3 (2521.7 , 4402.1)	84.6 (62.3 , 109.2)	9000.8 (6283.1 , 12315.3)	113.2 (80 , 153.4)	0.88 (0.38 , 1.37)
Malaysia	2005.8 (1520.6 , 2561)	21.5 (16.4 , 27.4)	5398.4 (4068.2 , 6957.5)	18.4 (13.9 , 23.6)	-0.72 (-0.89 , -0.56)

Maldives	31.1 (21.5 , 42.6)	31.9 (22.2 , 43.5)	40 (29.2 , 52.3)	11.4 (8.4 , 14.8)	-3.84 (-4.11 , -3.56)
Mali	284.5 (206.9 , 372.8)	6.6 (4.9 , 8.7)	798.9 (538.4 , 1153.1)	8.5 (5.7 , 12.1)	1.27 (1.08 , 1.46)
Malta	144.2 (111.5 , 178.5)	33.5 (25.9 , 41.4)	176.1 (131.3 , 225.9)	20.9 (15.8 , 26.8)	-1.39 (-1.5 , -1.28)
Marshall Islands	3 (1.8 , 4.7)	16.9 (10 , 27.3)	6.2 (3.9 , 9.7)	15.4 (9.4 , 23.3)	-0.13 (-0.24 , -0.02)
Mauritania	131.9 (87.4 , 187.9)	12.6 (8.3 , 17.8)	389.2 (232.4 , 597.1)	16.8 (10.1 , 25.7)	1.35 (0.97 , 1.72)
Mauritius	198 (161.3 , 236.4)	26.1 (21.2 , 31)	494.3 (400.4 , 587.9)	25.8 (21 , 30.7)	-0.79 (-1.3 , -0.27)
Mexico	5336 (4200.2 , 6464)	12.9 (10 , 15.7)	6176.5 (4605.2 , 7804.1)	4.8 (3.6 , 6.1)	-3.27 (-3.35 , -3.18)
Micronesia (Federated States of)	13.3 (9.2 , 18.6)	25.6 (17.7 , 35.3)	18.7 (12.2 , 26.6)	21.5 (14.2 , 30.5)	-0.64 (-0.68 , -0.61)
Monaco	30.2 (19.3 , 42.8)	49.4 (31.8 , 69.5)	38.1 (24.4 , 57.7)	44.5 (28.8 , 67.6)	-0.25 (-0.48 , -0.03)
Mongolia	1186.4 (865.1 , 1611.4)	112.8 (82.2 , 152.4)	2226.7 (1553.9 , 3051.4)	92.9 (64.9 , 124)	-0.73 (-0.88 , -0.58)
Montenegro	203.2 (146.3 , 270)	30.7 (22.1 , 40.8)	302 (210.1 , 410.7)	30.5 (21.2 , 41.4)	0.14 (-0.02 , 0.31)
Morocco	619.3 (432.7 , 834.9)	4.2 (3 , 5.7)	1308.4 (851.5 , 1837)	3.6 (2.3 , 5)	-0.5 (-0.75 , -0.26)
Mozambique	1882.3 (1363 , 2511.7)	31 (22.4 , 41)	4006.4 (2793.9 , 5412.8)	34.3 (24.1 , 45.9)	0.95 (0.7 , 1.19)
Myanmar	11975.5 (8528.7 , 16261.4)	48.1 (34.5 , 65.3)	10225.4 (7302.9 , 14306.2)	19.6 (13.9 , 27.4)	-3.28 (-3.43 , -3.14)
Namibia	80.7 (59.1 , 109.5)	12.1 (8.9 , 16.2)	141.7 (98.9 , 193.5)	9.9 (7.1 , 13.1)	-0.97 (-1.36 , -0.57)
Nauru	1.6 (1 , 2.2)	29.7 (18.6 , 42.5)	1.5 (1 , 2.1)	22.1 (14.4 , 31.2)	-1.3 (-1.48 , -1.11)
Nepal	6351.6 (4573 , 8629.4)	63.2 (45.6 , 85.2)	10002.2 (6806.8 , 14419)	41.7 (28.6 , 59.4)	-1.33 (-1.68 , -0.98)
Netherlands	12040.4 (9597.5 , 14248.5)	62.4 (49.8 , 73.7)	17411.8 (13205.4 , 21651.9)	49.8 (38.1 , 61.6)	-0.68 (-0.99 , -0.38)
New Zealand	1529.3 (1171.5 , 1870)	38.9 (29.8 , 47.4)	1459.2 (1108 , 1870)	17.4 (13.3 , 22.2)	-2.8 (-2.97 , -2.63)
Nicaragua	77.8 (56.8 , 101.5)	5.1 (3.7 , 6.6)	180.8 (123.6 , 255.3)	3.7 (2.5 , 5.2)	-0.86 (-1.06 , -0.67)
Niger	212.5 (141.4 , 301.6)	7 (4.7 , 9.8)	884.8 (574.3 , 1327.7)	9.9 (6.6 , 14.7)	1.83 (1.58 , 2.08)
Nigeria	2138.2 (1451 , 3157.6)	4.6 (3.2 , 6.7)	5811.5 (3834.5 , 8329.9)	5.7 (3.8 , 8.1)	1.14 (0.91 , 1.36)
Niue	0.3 (0.2 , 0.5)	16 (10.5 , 23.8)	0.3 (0.2 , 0.5)	15.3 (10.2 , 23)	-0.45 (-0.58 , -0.32)
North Macedonia	382.4 (277.4 , 489.6)	19.2 (13.9 , 24.6)	595.8 (408.6 , 818.8)	17.4 (11.9 , 23.9)	-0.37 (-0.67 , -0.08)
Northern Mariana Islands	2.1 (1.5 , 2.9)	9.7 (6.9 , 13.9)	9.3 (6.9 , 12.3)	15.9 (11.7 , 21.3)	2.71 (2.12 , 3.3)
Norway	1593.5 (1290.3 , 1914.5)	25.5 (20.6 , 30.4)	1664.5 (1254.2 , 2098.9)	17.5 (13.4 , 21.9)	-1.56 (-1.78 , -1.34)
Oman	73.7 (46.4 , 109.3)	10.5 (6.6 , 15.4)	151 (98.5 , 218.2)	7.1 (4.7 , 10.4)	-0.78 (-0.96 , -0.59)
Pakistan	33700.2 (26553.2 , 41196.3)	58.5 (46 , 71.3)	63042.4 (46967.6 , 83794.7)	48.7 (36.6 , 64.5)	-1.02 (-1.31 , -0.74)
Palau	2.4 (1.7 , 3.4)	23 (16 , 31.8)	5 (3.6 , 7)	19.8 (14.4 , 27.4)	-0.57 (-0.64 , -0.49)
Palestine	106.5 (70.2 , 154.8)	12.6 (8.4 , 18.3)	199.5 (136.9 , 279)	8 (5.5 , 11.2)	-1.85 (-2.08 , -1.63)
Panama	149 (113.4 , 191.3)	10.2 (7.8 , 13.1)	198.5 (132.4 , 279.5)	4.5 (3 , 6.3)	-2.74 (-2.87 , -2.61)
Papua New Guinea	231.1 (139 , 364.6)	10.8 (6.6 , 16.8)	484.6 (321.2 , 732.4)	7.8 (5.1 , 11.9)	-1.32 (-1.44 , -1.19)
Paraguay	784.9 (580 , 1019.5)	35.3 (26 , 45.6)	1932.2 (1289.4 , 2827.9)	32.7 (21.8 , 47.4)	-0.53 (-0.73 , -0.34)
Peru	507 (355 , 695.6)	4.3 (3 , 5.9)	1003.1 (666.8 , 1513.9)	3 (2 , 4.5)	-1.7 (-2.04 , -1.36)
Philippines	4743.9 (3713.9 , 6009.1)	14.8 (11.7 , 18.8)	10284.1 (7749.1 , 13152.7)	11.6 (8.7 , 14.8)	-0.89 (-1.08 , -0.69)
Poland	20743.5 (17203.2 , 24318.5)	47.5 (39.4 , 55.7)	20595.5 (16206 , 25338.1)	30.9 (24.4 , 37.9)	-1.59 (-1.77 , -1.4)
Portugal	5911.5 (4496.6 , 7439.6)	43.2 (32.9 , 54.1)	5085.7 (3884.7 , 6358.8)	25.9 (19.9 , 32.2)	-1.48 (-1.73 , -1.22)
Puerto Rico	1376.1 (921.9 , 2006.9)	38.1 (25.6 , 55.5)	804.6 (521.2 , 1243.1)	12.6 (8.3 , 19)	-3.49 (-3.69 , -3.28)
Qatar	38 (23.3 , 56.8)	37.1 (23 , 53.2)	141.4 (83.5 , 210.3)	14.7 (8.4 , 22.7)	-3.24 (-3.91 , -2.56)
Republic of Korea	28055.7 (20966.3 , 35520.1)	87.2 (65.5 , 110.5)	23348.2 (16493.7 , 31424.2)	24.2 (17.1 , 32.7)	-4.66 (-4.85 , -4.47)
Republic of Moldova	1570.5 (1162.6 , 1997.6)	33.4 (24.8 , 42.2)	1651.5 (1284.6 , 2045.6)	27.5 (21.4 , 33.9)	-0.45 (-0.89 , -0.02)
Romania	4774.1 (3752.3 , 5873.8)	16.6 (13.1 , 20.4)	8736.2 (6747.5 , 10936.6)	27.1 (21.1 , 33.8)	1.13 (0.77 , 1.5)

Russian Federation	99270.2 (82162.4 , 115375.9)	52.7 (43.6 , 61.2)	85155.8 (68286.8 , 102693)	36 (28.9 , 43.3)	-1.45 (-1.61 , -1.29)
Rwanda	3589.9 (2295.7 , 4923.7)	119.6 (78.1 , 163.4)	4294.1 (2898.3 , 6056.6)	66.3 (45.3 , 93.5)	-2.95 (-3.36 , -2.55)
Saint Kitts and Nevis	6.5 (4.4 , 9.3)	18.7 (12.9 , 26)	10.1 (7 , 13.8)	13.4 (9.2 , 18.5)	-0.81 (-0.94 , -0.67)
Saint Lucia	26.8 (19.9 , 34.1)	31.1 (23.1 , 39.7)	51.9 (35.9 , 70.8)	21 (14.5 , 28.5)	-1.33 (-1.52 , -1.14)
Saint Vincent and the Grenadines	7.1 (5.3 , 9)	10.3 (7.6 , 13)	15.8 (11.6 , 20.9)	10.7 (7.9 , 14.2)	0.27 (-0.02 , 0.55)
Samoa	9.2 (6.5 , 12.1)	10.2 (7.2 , 13.3)	12.3 (8.9 , 16.6)	8.1 (5.9 , 10.9)	-1 (-1.12 , -0.89)
San Marino	6.2 (4.2 , 8.8)	18.5 (12.6 , 26.1)	5.4 (3 , 8.7)	8.1 (4.4 , 13.2)	-1.86 (-2.17 , -1.56)
Sao Tome and Principe	3 (2.1 , 4.1)	4.4 (3.1 , 6.1)	13.6 (9 , 19.4)	10.9 (7.3 , 15.4)	3.43 (3.22 , 3.65)
Saudi Arabia	743.2 (449 , 1179.1)	12.4 (7.4 , 19.5)	2314.7 (1457.4 , 3365.6)	10.9 (6.7 , 15.7)	-0.77 (-0.91 , -0.62)
Senegal	403.6 (285.9 , 558.1)	11.6 (8.2 , 16)	1270.3 (859.3 , 1869.4)	15 (10.1 , 21.7)	1.25 (0.94 , 1.55)
Serbia	3162.7 (2099.1 , 4530.9)	25.9 (17.4 , 36.9)	3637 (2361.7 , 5362.5)	23.9 (15.4 , 35.3)	-0.31 (-0.55 , -0.07)
Seychelles	31.6 (22.9 , 41.8)	56.9 (41.3 , 75.1)	54.6 (40.5 , 71.8)	43.9 (33 , 57.4)	-0.79 (-0.97 , -0.62)
Sierra Leone	249 (175.3 , 341.8)	11.9 (8.4 , 16.3)	695.3 (486.5 , 991.9)	16.9 (11.9 , 23.9)	1.97 (1.65 , 2.29)
Singapore	875.5 (641.7 , 1137.6)	39 (28.2 , 50.8)	831 (612.9 , 1062.7)	9.4 (6.9 , 11.9)	-4.53 (-4.85 , -4.21)
Slovakia	2882.8 (1970.7 , 4016)	49.3 (34 , 68.4)	2779.5 (1865.4 , 3981.3)	30.6 (20.4 , 44.1)	-1.55 (-1.67 , -1.42)
Slovenia	1139.5 (880.7 , 1403.4)	45.6 (35.3 , 56.1)	985.3 (714.5 , 1252.7)	24.9 (18 , 31.8)	-2.1 (-2.28 , -1.93)
Solomon Islands	34.9 (21.4 , 51.2)	22.8 (14.4 , 33.3)	78.3 (49.3 , 117.7)	19.8 (12.8 , 29.4)	-0.4 (-0.52 , -0.29)
Somalia	1971.4 (1173.1 , 3010.3)	70.2 (42.4 , 105.8)	2840.6 (1800.4 , 4373.1)	40.4 (26.6 , 60.7)	-2.05 (-2.18 , -1.93)
South Africa	21305.4 (16822.1 , 26605.5)	96.5 (75.9 , 120.7)	26759.2 (20793.9 , 33463)	53.7 (42 , 66.9)	-2.58 (-3.05 , -2.11)
South Sudan	1817.2 (1190.9 , 2676.7)	68.2 (44.6 , 100.6)	2000 (1234.8 , 2931.9)	45.7 (29.1 , 67.5)	-1.65 (-1.89 , -1.41)
Spain	30742.6 (25166.7 , 36355.9)	59.7 (49.2 , 70.3)	21147.7 (16267 , 25598.8)	24.6 (19 , 29.6)	-2.89 (-3.06 , -2.73)
Sri Lanka	5231.8 (3995 , 6803.5)	46.7 (35.9 , 60.4)	8016.4 (4784 , 11643.5)	28.7 (17.2 , 41.4)	-1.17 (-1.45 , -0.88)
Sudan	3151 (2036.1 , 4563.2)	33.1 (21.5 , 47.9)	6356.5 (3821.9 , 9683.1)	32.4 (19.8 , 49.4)	-0.18 (-0.24 , -0.13)
Suriname	37.2 (27.5 , 50.2)	13.8 (10.3 , 18.6)	77.3 (51.5 , 110.7)	11.4 (7.6 , 16.3)	-0.48 (-0.7 , -0.25)
Sweden	3750.8 (2984.3 , 4558.5)	26 (20.8 , 31.4)	4578.8 (3368.5 , 5886)	22.3 (16.4 , 28.5)	-0.38 (-0.54 , -0.21)
Switzerland	5374 (4297.5 , 6489.8)	55.4 (44.7 , 67.1)	4369.3 (3353.3 , 5430.9)	25.2 (19.4 , 31.2)	-2.24 (-2.41 , -2.06)
Syrian Arab Republic	413.5 (291.6 , 565.8)	7.8 (5.5 , 10.5)	939.1 (602.9 , 1345.5)	7 (4.6 , 9.9)	-0.61 (-0.78 , -0.44)
Taiwan (Province of China)	21231.5 (17163.2 , 25103.5)	125.4 (102.2 , 148.1)	37800.3 (29999.1 , 45812.2)	92.9 (73.5 , 112.5)	-0.96 (-1.31 , -0.6)
Tajikistan	3355.4 (2360.2 , 4473.2)	118.7 (83.5 , 156.4)	2683.1 (1615.2 , 4211.3)	46.2 (27.8 , 72.1)	-2.95 (-3.22 , -2.69)
Thailand	20339.2 (14442.5 , 29468.3)	53.8 (38.2 , 77.2)	40832.6 (29373.7 , 56970)	37 (26.7 , 51.6)	-1.57 (-1.71 , -1.43)
Timor-Leste	56.1 (34.1 , 83.1)	18.3 (11.5 , 27.1)	123.2 (80.3 , 174.6)	14.1 (9.2 , 20.1)	-0.85 (-1.09 , -0.61)
Togo	231.6 (152.3 , 337.1)	18 (11.9 , 26.4)	1247.1 (809.1 , 1837.7)	29.2 (19 , 42.7)	2.04 (1.84 , 2.25)
Tokelau	0.2 (0.1 , 0.3)	15.1 (9 , 22.8)	0.2 (0.1 , 0.2)	11.6 (7.7 , 16.3)	-1.06 (-1.14 , -0.97)
Tonga	11.2 (7.8 , 15.1)	19.7 (13.7 , 26.6)	14.3 (9.9 , 20.4)	17.6 (12.3 , 25.1)	-0.57 (-0.79 , -0.34)
Trinidad and Tobago	133.2 (103.1 , 164.3)	15.8 (12.2 , 19.6)	211.6 (140.6 , 298.1)	10.8 (7.2 , 15.2)	-1.13 (-1.35 , -0.92)
Tunisia	350.5 (251.6 , 471.1)	6.9 (5 , 9.1)	893.4 (546.1 , 1327)	6.5 (4 , 9.7)	-0.33 (-0.4 , -0.26)
Turkey	9302.6 (6740 , 12490)	25.2 (18.4 , 33.6)	13809.1 (9371 , 18683.3)	14.3 (9.7 , 19.3)	-2.11 (-2.34 , -1.88)
Turkmenistan	5421.5 (4407.5 , 6456.1)	268.4 (219.3 , 316.7)	2388.5 (1630.8 , 3289.6)	56.5 (38.8 , 77.8)	-5.13 (-5.59 , -4.67)
Tuvalu	1.4 (1 , 2)	19.4 (13.4 , 26.9)	1.7 (1.2 , 2.4)	15.3 (10.7 , 21.6)	-0.8 (-0.87 , -0.74)
Uganda	2698.3 (1913.3 , 3630.8)	39.9 (28.2 , 53.5)	5790.7 (4120.5 , 7976)	36.2 (25.8 , 49.9)	-1.04 (-1.45 , -0.63)
Ukraine	31189 (25266.1 , 37272.9)	43.2 (35 , 51.6)	19525.8 (12182.7 , 29208.1)	26.8 (16.8 , 40)	-1.9 (-2.05 , -1.74)

United Arab Emirates	73.3 (47.9 , 113.9)	15.5 (10 , 24.4)	376 (251.3 , 536.4)	10 (6.6 , 14.1)	-0.42 (-1.01 , 0.18)
United Kingdom	73984.3 (59094.4 , 86817.8)	84.5 (67.7 , 99)	71016.2 (53586.5 , 88110.7)	56 (42.7 , 69.4)	-1.46 (-1.69 , -1.23)
United Republic of Tanzania	8285.5 (5989.4 , 11183.5)	71.9 (52.3 , 98.1)	10312.9 (7030.3 , 14813.9)	37.5 (25.6 , 53.8)	-2.63 (-2.83 , -2.43)
United States of America	168945.8 (138654.1 , 196419.7)	56.5 (46.3 , 65.6)	231871.1 (180692.4 , 279640.3)	40.5 (31.7 , 48.8)	-1.19 (-1.37 , -1.01)
United States Virgin Islands	17.9 (10.4 , 28.4)	20 (11.5 , 31.6)	20.1 (11.1 , 33.2)	11.4 (6.4 , 18.5)	-1.78 (-1.96 , -1.6)
Uruguay	2882.4 (2256.8 , 3546.7)	74.8 (58.6 , 91.9)	2142.3 (1653.8 , 2644.9)	41.9 (32.4 , 51.6)	-2.22 (-2.5 , -1.95)
Uzbekistan	7127.4 (5412.4 , 8978.3)	59.3 (45 , 74.9)	4834 (3621.9 , 6232.8)	17.2 (12.8 , 22.3)	-4.44 (-5.03 , -3.84)
Vanuatu	10.4 (7.1 , 15.2)	14.9 (10.2 , 21.9)	20.8 (14.5 , 28.8)	10.6 (7.4 , 14.7)	-1.31 (-1.42 , -1.21)
Venezuela (Bolivarian Republic of)	1523.4 (1194.1 , 1912.2)	15.7 (12.2 , 19.8)	2165.4 (1315.1 , 3080)	7 (4.3 , 10)	-2.77 (-2.92 , -2.62)
Viet Nam	9148.4 (6324.3 , 12857.5)	22.1 (15.4 , 30.9)	26254.5 (18083.4 , 35587.5)	24.1 (16.8 , 32.4)	0.42 (0.36 , 0.49)
Yemen	3034.7 (2020.4 , 4345)	57.6 (38.8 , 81.7)	5944.8 (3666.2 , 8615.1)	40.7 (25.7 , 58.2)	-1.49 (-1.65 , -1.32)
Zambia	1993.6 (1415.9 , 2720.2)	67.4 (48 , 91.1)	3519 (2319.4 , 4978.1)	47.9 (32.4 , 67.5)	-1.7 (-2.02 , -1.38)
Zimbabwe	4037.4 (2781.8 , 5508)	96.1 (66.2 , 131.5)	7297.2 (4945.4 , 9877.7)	98.2 (68 , 129.9)	-0.03 (-0.39 , 0.33)

sTable 3 Tobacco-Related Esophageal Cancer Years Lived with Disability (YLDs) Between 1990 and 2021 for Both Sexes, Each SDI Regions and All countries, With EAPC Between 1990 and 2021.

	1990 YLDs		2021 YLDs		
	Case number (95% UI)	ASR/100,000 persons (95% UI)	Case number (95% UI)	ASR/100,000 persons (95% UI)	EAPC% (95% CI) 1990–2021
Global	37913.2 (26500.5 , 51322.3)	0.9 (0.7 , 1.3)	63202.3 (42826.7 , 87266.3)	0.7 (0.5 , 1)	-0.96 (-1.07 , -0.86)
**Sex**					
Male	33972.2 (23632 , 46126.9)	1.8 (1.3 , 2.4)	57905.6 (39226.3 , 80320.3)	1.4 (1 , 2)	-0.88 (-0.98 , -0.78)
Female	3940.9 (2629 , 5563.8)	0.2 (0.1 , 0.3)	5296.7 (3524.2 , 7611.1)	0.1 (0.1 , 0.2)	-1.84 (-1.99 , -1.68)
**SDI**					
High SDI	8505.8 (6027.1 , 11378.1)	0.8 (0.6 , 1)	12728 (8788.3 , 17509.8)	0.6 (0.4 , 0.9)	-0.81 (-1.04 , -0.57)
High-middle SDI	11901.8 (8016.8 , 16326)	1.2 (0.8 , 1.6)	21524.6 (14106.3 , 31099.1)	1.1 (0.7 , 1.5)	-0.37 (-0.47 , -0.26)
Middle SDI	14279.9 (9611 , 19996)	1.3 (0.9 , 1.9)	23381.7 (15228.1 , 32589.6)	0.9 (0.6 , 1.2)	-1.61 (-1.7 , -1.52)
Low-middle SDI	2492.3 (1710.7 , 3329.8)	0.4 (0.3 , 0.5)	4367 (2990.6 , 6078.5)	0.3 (0.2 , 0.4)	-1.05 (-1.12 , -0.98)
Low SDI	717.1 (503.1 , 984.7)	0.3 (0.2 , 0.4)	1179.3 (797.2 , 1650.8)	0.2 (0.2 , 0.3)	-1.17 (-1.26 , -1.09)
**Country**					
Afghanistan	18.8 (9 , 31.1)	0.3 (0.1 , 0.4)	25.5 (14.1 , 43.1)	0.2 (0.1 , 0.4)	-0.33 (-0.67 , 0.02)
Albania	5 (3.2 , 7.3)	0.2 (0.2 , 0.4)	9 (5.3 , 14)	0.2 (0.1 , 0.3)	-0.23 (-0.47 , 0.02)
Algeria	8.7 (5.5 , 12.7)	0.1 (0 , 0.1)	24.3 (14.7 , 36.3)	0.1 (0 , 0.1)	-0.2 (-0.27 , -0.12)
American Samoa	0 (0 , 0)	0.1 (0.1 , 0.1)	0.1 (0 , 0.1)	0.1 (0.1 , 0.2)	1.05 (0.73 , 1.37)
Andorra	0.2 (0.1 , 0.3)	0.3 (0.2 , 0.5)	0.2 (0.1 , 0.4)	0.2 (0.1 , 0.3)	-1.55 (-1.81 , -1.29)
Angola	21.5 (12.4 , 33.6)	0.5 (0.3 , 0.8)	42.2 (24.9 , 69.6)	0.3 (0.2 , 0.6)	-1.5 (-1.77 , -1.23)
Antigua and Barbuda	0.1 (0.1 , 0.1)	0.2 (0.1 , 0.3)	0.2 (0.1 , 0.2)	0.1 (0.1 , 0.2)	-0.55 (-0.8 , -0.29)
Argentina	198.5 (127.3 , 283.1)	0.6 (0.4 , 0.9)	170.3 (108.2 , 245)	0.3 (0.2 , 0.4)	-2.14 (-2.42 , -1.86)
Armenia	9.4 (6.2 , 13)	0.3 (0.2 , 0.5)	5.9 (4 , 8.1)	0.1 (0.1 , 0.2)	-2.97 (-3.31 , -2.64)
Australia	90.5 (58 , 128.3)	0.5 (0.3 , 0.6)	117.9 (73.8 , 175.1)	0.3 (0.2 , 0.4)	-1.74 (-1.86 , -1.62)
Austria	36.2 (23.6 , 50.1)	0.3 (0.2 , 0.5)	57.8 (37.5 , 82)	0.3 (0.2 , 0.5)	0.38 (0.18 , 0.59)
Azerbaijan	36.9 (22.9 , 55.7)	0.7 (0.4 , 1.1)	55.9 (32.3 , 87.2)	0.5 (0.3 , 0.8)	-0.6 (-0.83 , -0.36)
Bahamas	0.6 (0.3 , 1)	0.4 (0.2 , 0.6)	1.2 (0.7 , 1.8)	0.3 (0.2 , 0.4)	-0.3 (-0.58 , -0.03)
Bahrain	0.6 (0.4 , 1)	0.4 (0.2 , 0.6)	1.7 (0.9 , 2.8)	0.2 (0.1 , 0.4)	-2.63 (-2.95 , -2.31)
Bangladesh	337.9 (196.4 , 507.3)	0.7 (0.4 , 1.1)	601.3 (363.7 , 950.6)	0.4 (0.3 , 0.7)	-1.56 (-1.69 , -1.44)
Barbados	0.9 (0.5 , 1.3)	0.3 (0.2 , 0.5)	1 (0.6 , 1.7)	0.2 (0.1 , 0.3)	-1.57 (-1.77 , -1.36)
Belarus	50.5 (34.3 , 73.2)	0.4 (0.3 , 0.5)	65.6 (40.3 , 99.5)	0.4 (0.3 , 0.6)	-0.09 (-0.23 , 0.06)
Belgium	84.5 (58.2 , 121.1)	0.6 (0.4 , 0.8)	122.5 (78.4 , 176.3)	0.6 (0.4 , 0.8)	-0.06 (-0.31 , 0.2)
Belize	0.1 (0.1 , 0.2)	0.1 (0.1 , 0.2)	0.3 (0.2 , 0.5)	0.1 (0.1 , 0.2)	0.33 (-0.12 , 0.77)
Benin	2 (1.3 , 3)	0.1 (0.1 , 0.1)	5.9 (3.6 , 9.5)	0.1 (0.1 , 0.2)	0.7 (0.55 , 0.85)
Bermuda	0.2 (0.2 , 0.4)	0.4 (0.2 , 0.6)	0.3 (0.2 , 0.5)	0.3 (0.2 , 0.4)	-0.93 (-1.22 , -0.64)
Bhutan	1.2 (0.7 , 1.9)	0.5 (0.3 , 0.8)	2.1 (1.2 , 3.4)	0.4 (0.2 , 0.6)	-1.13 (-1.32 , -0.93)
Bolivia (Plurinational State of)	3.4 (2.1 , 5.3)	0.1 (0.1 , 0.2)	6.9 (4 , 11.4)	0.1 (0 , 0.1)	-0.81 (-1.04 , -0.58)
Bosnia and Herzegovina	12.5 (8 , 17.9)	0.3 (0.2 , 0.4)	18.4 (11.2 , 28.4)	0.3 (0.2 , 0.5)	0.25 (0.07 , 0.43)
Botswana	5 (2.9 , 8.1)	0.9 (0.5 , 1.4)	8.6 (5.3 , 13.3)	0.6 (0.4 , 0.9)	-1.6 (-1.85 , -1.34)
Brazil	719.2 (502.2 , 985)	0.8 (0.6 , 1.1)	878.3 (598.4 , 1243)	0.3 (0.2 , 0.5)	-2.88 (-2.99 , -2.77)

Brunei Darussalam	0.4 (0.2 , 0.6)	0.4 (0.2 , 0.7)	0.8 (0.5 , 1.3)	0.2 (0.2 , 0.4)	-1.57 (-1.73 , -1.4)
Bulgaria	42.4 (28.8 , 58.1)	0.3 (0.2 , 0.5)	37.8 (25.3 , 53.5)	0.3 (0.2 , 0.4)	-0.61 (-0.83 , -0.38)
Burkina Faso	4.4 (2.8 , 6.6)	0.1 (0.1 , 0.1)	15.4 (8.8 , 23.6)	0.2 (0.1 , 0.2)	2.09 (1.87 , 2.31)
Burundi	18.9 (11.6 , 28.7)	0.8 (0.5 , 1.2)	16.2 (9.7 , 25.6)	0.3 (0.2 , 0.5)	-3.49 (-3.73 , -3.25)
Cabo Verde	0.7 (0.5 , 1)	0.3 (0.2 , 0.5)	2.3 (1.4 , 3.4)	0.5 (0.3 , 0.7)	0.69 (0.25 , 1.14)
Cambodia	25.2 (15.6 , 37.1)	0.6 (0.3 , 0.8)	46.3 (28.6 , 68.6)	0.4 (0.2 , 0.6)	-1.56 (-1.66 , -1.46)
Cameroon	5.6 (3.7 , 8.8)	0.1 (0.1 , 0.2)	25.6 (14.8 , 41.7)	0.2 (0.1 , 0.3)	1.9 (1.67 , 2.13)
Canada	202.4 (135.4 , 283.1)	0.6 (0.4 , 0.9)	362 (244.4 , 534.3)	0.5 (0.3 , 0.7)	-0.61 (-0.79 , -0.43)
Central African Republic	5.4 (3.2 , 8.3)	0.4 (0.3 , 0.6)	6.7 (3.7 , 10.7)	0.3 (0.2 , 0.4)	-1.72 (-1.88 , -1.57)
Chad	2.6 (1.6 , 3.9)	0.1 (0.1 , 0.1)	10.2 (5.6 , 16.1)	0.2 (0.1 , 0.3)	2.43 (2.23 , 2.64)
Chile	41.3 (27.1 , 61)	0.4 (0.3 , 0.6)	32.4 (20.4 , 47.5)	0.1 (0.1 , 0.2)	-4.08 (-4.32 , -3.84)
China	21383.4 (13980.9 , 30220.9)	2.5 (1.6 , 3.5)	38990.7 (24784.9 , 55753)	1.8 (1.1 , 2.5)	-1.19 (-1.31 , -1.06)
Colombia	36.9 (24.1 , 52.1)	0.2 (0.1 , 0.3)	29.1 (18.2 , 43.5)	0.1 (0 , 0.1)	-5.08 (-5.31 , -4.85)
Comoros	2.1 (1.2 , 3.3)	1 (0.6 , 1.6)	3.3 (1.9 , 5.4)	0.7 (0.4 , 1.1)	-1.78 (-1.93 , -1.62)
Congo	5.2 (2.9 , 8.3)	0.5 (0.3 , 0.7)	10 (5.7 , 15.7)	0.4 (0.2 , 0.6)	-0.99 (-1.22 , -0.76)
Cook Islands	0 (0 , 0)	0.2 (0.1 , 0.3)	0 (0 , 0.1)	0.2 (0.1 , 0.3)	-0.79 (-0.9 , -0.68)
Costa Rica	3.5 (2.3 , 5.2)	0.2 (0.1 , 0.3)	5 (3.1 , 7.4)	0.1 (0.1 , 0.1)	-2.89 (-3.07 , -2.72)
Coted'Ivoire	1.9 (1.2 , 2.9)	0 (0 , 0.1)	5.2 (3.1 , 8.3)	0 (0 , 0.1)	-0.4 (-0.73 , -0.06)
Croatia	35.4 (23.2 , 50.7)	0.6 (0.4 , 0.8)	30.4 (19.7 , 44.3)	0.4 (0.2 , 0.5)	-1.22 (-1.38 , -1.06)
Cuba	41.6 (27.2 , 59.6)	0.4 (0.3 , 0.6)	95.6 (62 , 145.4)	0.5 (0.3 , 0.7)	0.88 (0.77 , 0.99)
Cyprus	1.3 (0.8 , 1.9)	0.2 (0.1 , 0.3)	4.2 (2.5 , 6.3)	0.2 (0.1 , 0.3)	1.35 (1.07 , 1.63)
Czechia	49.5 (33.3 , 70.4)	0.4 (0.2 , 0.5)	74.9 (47.1 , 113.1)	0.4 (0.2 , 0.6)	-0.05 (-0.18 , 0.08)
Democratic People's Republic of Korea	165.7 (91 , 255)	1 (0.5 , 1.5)	297.7 (170.1 , 458.3)	0.9 (0.5 , 1.3)	-0.3 (-0.4 , -0.2)
Democratic Republic of the Congo	42.4 (25.4 , 64.1)	0.3 (0.1 , 0.4)	70.3 (39.2 , 109.7)	0.2 (0.1 , 0.3)	-1.27 (-1.45 , -1.09)
Denmark	58.1 (38.8 , 81.2)	0.7 (0.5 , 1)	75.6 (50.6 , 109.2)	0.6 (0.4 , 0.9)	-0.57 (-0.71 , -0.42)
Djibouti	1.1 (0.6 , 1.8)	0.8 (0.4 , 1.3)	4.1 (2.2 , 6.7)	0.6 (0.3 , 1)	-0.82 (-0.9 , -0.74)
Dominica	0.1 (0.1 , 0.2)	0.2 (0.1 , 0.3)	0.2 (0.1 , 0.2)	0.2 (0.1 , 0.3)	-0.14 (-0.39 , 0.1)
Dominican Republic	6.4 (3.9 , 9.9)	0.2 (0.1 , 0.3)	17.6 (10.2 , 28.5)	0.2 (0.1 , 0.3)	0.31 (0.12 , 0.5)
Ecuador	5.8 (3.8 , 8.3)	0.1 (0.1 , 0.2)	6.8 (4.1 , 10.6)	0 (0 , 0.1)	-2.71 (-3.01 , -2.42)
Egypt	26.9 (17.3 , 39.1)	0.1 (0.1 , 0.1)	63.4 (40 , 95.1)	0.1 (0.1 , 0.2)	0.09 (-0.08 , 0.26)
El Salvador	1.7 (1.1 , 2.7)	0.1 (0 , 0.1)	3.7 (2.2 , 5.9)	0.1 (0 , 0.1)	0.02 (-0.19 , 0.24)
Equatorial Guinea	0.8 (0.5 , 1.3)	0.4 (0.2 , 0.6)	1.5 (0.8 , 2.4)	0.3 (0.2 , 0.4)	-1.14 (-1.69 , -0.58)
Eritrea	7.3 (3.8 , 11.6)	0.5 (0.3 , 0.8)	8.7 (4.4 , 14.9)	0.3 (0.1 , 0.4)	-2.55 (-2.75 , -2.34)
Estonia	7.5 (4.8 , 10.9)	0.4 (0.2 , 0.5)	7.5 (4.9 , 11.1)	0.3 (0.2 , 0.5)	-0.66 (-0.84 , -0.48)
Eswatini	1.6 (1 , 2.4)	0.5 (0.3 , 0.8)	2.4 (1.4 , 3.8)	0.4 (0.2 , 0.6)	-0.82 (-1.39 , -0.25)
Ethiopia	30.9 (18.7 , 48.5)	0.1 (0.1 , 0.2)	28.2 (16.4 , 44.7)	0.1 (0 , 0.1)	-2.67 (-2.97 , -2.37)
Fiji	0.6 (0.4 , 0.9)	0.2 (0.1 , 0.2)	1.1 (0.6 , 1.7)	0.1 (0.1 , 0.2)	-0.56 (-0.71 , -0.42)
Finland	23.6 (15.5 , 34.4)	0.3 (0.2 , 0.5)	34.5 (22.3 , 51.1)	0.3 (0.2 , 0.4)	-0.15 (-0.3 , -0.01)
France	783.6 (522.3 , 1107.9)	1 (0.7 , 1.4)	663.9 (433.8 , 953.5)	0.5 (0.3 , 0.7)	-1.87 (-1.99 , -1.75)
Gabon	1.8 (1 , 2.8)	0.3 (0.2 , 0.5)	3.4 (1.9 , 5.4)	0.3 (0.2 , 0.5)	-0.14 (-0.24 , -0.04)
Gambia	0.3 (0.2 , 0.4)	0.1 (0 , 0.1)	0.7 (0.4 , 1)	0.1 (0 , 0.1)	-0.28 (-0.42 , -0.14)
Georgia	14.9 (9.9 , 21.2)	0.2 (0.2 , 0.3)	7.5 (4.9 , 10.7)	0.1 (0.1 , 0.2)	-0.9 (-1.43 , -0.35)

Germany	608.4 (405.6 , 863.5)	0.5 (0.3 , 0.7)	1026.6 (693.5 , 1469.4)	0.6 (0.4 , 0.8)	0.34 (0.09 , 0.6)
Ghana	3.3 (2 , 4.9)	0.1 (0 , 0.1)	11.7 (7.1 , 18.5)	0.1 (0 , 0.1)	1.54 (1.33 , 1.75)
Greece	51.5 (35.4 , 70.8)	0.3 (0.2 , 0.5)	61.9 (41.4 , 84.9)	0.3 (0.2 , 0.4)	-0.64 (-0.77 , -0.51)
Greenland	0.8 (0.5 , 1.2)	2.2 (1.4 , 3.5)	0.9 (0.6 , 1.4)	1.2 (0.7 , 1.8)	-1.86 (-1.94 , -1.78)
Grenada	0.2 (0.1 , 0.3)	0.3 (0.2 , 0.5)	0.2 (0.1 , 0.3)	0.2 (0.1 , 0.3)	-1.25 (-1.76 , -0.74)
Guam	0.1 (0.1 , 0.2)	0.1 (0.1 , 0.2)	0.3 (0.2 , 0.4)	0.1 (0.1 , 0.2)	0.57 (0.26 , 0.89)
Guatemala	2.6 (1.7 , 3.8)	0.1 (0.1 , 0.1)	5.2 (3.3 , 7.9)	0 (0 , 0.1)	-1.88 (-2.23 , -1.53)
Guinea	1.3 (0.8 , 1.9)	0 (0 , 0.1)	2.5 (1.5 , 4.1)	0 (0 , 0.1)	0.71 (0.52 , 0.91)
Guinea-Bissau	0.4 (0.2 , 0.6)	0.1 (0 , 0.1)	1.4 (0.8 , 2.2)	0.2 (0.1 , 0.3)	3.17 (2.77 , 3.56)
Guyana	0.4 (0.2 , 0.5)	0.1 (0.1 , 0.1)	0.6 (0.3 , 0.9)	0.1 (0 , 0.1)	0.08 (-0.11 , 0.26)
Haiti	5.2 (3.1 , 8.3)	0.1 (0.1 , 0.2)	6.3 (3.6 , 10.3)	0.1 (0 , 0.1)	-1.88 (-2.07 , -1.7)
Honduras	1.3 (0.8 , 1.9)	0.1 (0 , 0.1)	5.3 (3.2 , 8.4)	0.1 (0.1 , 0.1)	1.39 (1.19 , 1.6)
Hungary	81.2 (51.9 , 119.5)	0.6 (0.4 , 0.8)	65.6 (41.8 , 97.6)	0.4 (0.2 , 0.5)	-1.76 (-2.13 , -1.38)
Iceland	2.1 (1.4 , 2.9)	0.7 (0.5 , 1)	3.3 (2.1 , 4.7)	0.6 (0.4 , 0.8)	-0.89 (-1.05 , -0.74)
India	1850.9 (1288.5 , 2559.4)	0.4 (0.3 , 0.5)	3515.8 (2336.1 , 4921.5)	0.3 (0.2 , 0.4)	-1.15 (-1.26 , -1.05)
Indonesia	109.8 (71 , 154.1)	0.1 (0.1 , 0.2)	283.7 (182.8 , 414.5)	0.1 (0.1 , 0.2)	0.17 (0.06 , 0.28)
Iran (Islamic Republic of)	83.7 (52.5 , 119.7)	0.3 (0.2 , 0.5)	206.5 (135.1 , 288.6)	0.3 (0.2 , 0.4)	-0.41 (-0.55 , -0.28)
Iraq	10.9 (6.6 , 16.4)	0.1 (0.1 , 0.2)	30.7 (18.3 , 48.3)	0.1 (0.1 , 0.2)	-0.57 (-0.78 , -0.36)
Ireland	43.7 (29.1 , 61.4)	1.1 (0.7 , 1.5)	48.2 (30.4 , 68.9)	0.6 (0.4 , 0.9)	-1.61 (-1.76 , -1.47)
Israel	11 (7.1 , 15.4)	0.2 (0.1 , 0.3)	17.9 (12 , 25)	0.1 (0.1 , 0.2)	-1.51 (-1.69 , -1.33)
Italy	363.4 (250.9 , 515.5)	0.4 (0.3 , 0.6)	250.7 (171.4 , 360)	0.2 (0.1 , 0.3)	-2.57 (-2.63 , -2.51)
Jamaica	3.6 (2.3 , 5.2)	0.2 (0.1 , 0.3)	5.5 (3.3 , 9)	0.2 (0.1 , 0.3)	-0.7 (-1.19 , -0.21)
Japan	1943.6 (1381.5 , 2625.2)	1.1 (0.8 , 1.5)	3040.9 (2125.6 , 4205.6)	0.9 (0.6 , 1.2)	-0.87 (-1.21 , -0.52)
Jordan	1.8 (1.2 , 2.7)	0.1 (0.1 , 0.2)	8.4 (5.4 , 13)	0.1 (0.1 , 0.2)	-0.63 (-0.8 , -0.46)
Kazakhstan	163 (107.8 , 238.7)	1.2 (0.8 , 1.8)	54 (31.9 , 81.9)	0.3 (0.2 , 0.4)	-4.66 (-4.94 , -4.38)
Kenya	25.2 (15.6 , 38.8)	0.3 (0.2 , 0.5)	75.2 (45.8 , 112.8)	0.3 (0.2 , 0.5)	0.12 (-0.08 , 0.32)
Kiribati	0.2 (0.1 , 0.3)	0.6 (0.4 , 0.9)	0.4 (0.2 , 0.7)	0.5 (0.3 , 0.8)	-0.43 (-0.61 , -0.25)
Kuwait	1 (0.7 , 1.5)	0.2 (0.1 , 0.3)	2.9 (1.8 , 4.6)	0.1 (0.1 , 0.2)	-1.71 (-2.5 , -0.92)
Kyrgyzstan	26.4 (17.3 , 39.3)	0.9 (0.6 , 1.3)	15.1 (9.9 , 22.3)	0.3 (0.2 , 0.5)	-3.05 (-3.19 , -2.9)
Lao People's Democratic Republic	9.2 (5.6 , 14.1)	0.4 (0.3 , 0.7)	11.4 (7.2 , 17.4)	0.3 (0.2 , 0.4)	-1.86 (-1.94 , -1.78)
Latvia	12.3 (8.2 , 17.8)	0.3 (0.2 , 0.5)	10.6 (6.8 , 15.9)	0.3 (0.2 , 0.5)	-0.3 (-0.51 , -0.1)
Lebanon	3.6 (2.2 , 5.5)	0.2 (0.1 , 0.3)	8.9 (5.9 , 13.1)	0.1 (0.1 , 0.2)	0.13 (-0.08 , 0.35)
Lesotho	6.3 (3.5 , 10.2)	0.8 (0.4 , 1.2)	12.9 (7.7 , 19.4)	1.2 (0.7 , 1.7)	2.04 (1.71 , 2.36)
Liberia	0.9 (0.6 , 1.4)	0.1 (0.1 , 0.1)	3 (1.8 , 5.1)	0.1 (0.1 , 0.2)	2.16 (1.86 , 2.47)
Libya	3.3 (1.9 , 5.2)	0.2 (0.1 , 0.3)	11.7 (7.2 , 17.8)	0.2 (0.1 , 0.3)	1.12 (0.96 , 1.29)
Lithuania	15 (9.7 , 21.5)	0.3 (0.2 , 0.5)	18.8 (11.9 , 27.2)	0.4 (0.2 , 0.5)	0.38 (0.19 , 0.57)
Luxembourg	3.2 (1.9 , 4.7)	0.6 (0.4 , 0.9)	4.3 (2.6 , 6.7)	0.4 (0.2 , 0.6)	-1.16 (-1.39 , -0.93)
Madagascar	43.9 (26.6 , 66.3)	0.8 (0.5 , 1.2)	62.5 (34.2 , 101.2)	0.5 (0.3 , 0.7)	-1.87 (-1.95 , -1.79)
Malawi	29.9 (19.4 , 43.3)	0.8 (0.5 , 1.1)	78.8 (49.2 , 118.3)	1 (0.6 , 1.6)	0.92 (0.46 , 1.38)
Malaysia	21 (13.3 , 30)	0.2 (0.1 , 0.3)	60 (38.6 , 87.3)	0.2 (0.1 , 0.3)	-0.51 (-0.66 , -0.36)
Maldives	0.3 (0.2 , 0.5)	0.3 (0.2 , 0.5)	0.5 (0.3 , 0.7)	0.1 (0.1 , 0.2)	-3.18 (-3.41 , -2.94)
Mali	2.8 (1.7 , 4.1)	0.1 (0 , 0.1)	7.9 (4.6 , 12.4)	0.1 (0.1 , 0.1)	1.35 (1.16 , 1.53)

Malta	1.6 (1.1 , 2.3)	0.4 (0.2 , 0.5)	2.5 (1.6 , 3.8)	0.3 (0.2 , 0.4)	-0.76 (-0.88 , -0.64)
Marshall Islands	0 (0 , 0)	0.2 (0.1 , 0.3)	0.1 (0 , 0.1)	0.2 (0.1 , 0.3)	-0.03 (-0.12 , 0.07)
Mauritania	1.2 (0.7 , 2)	0.1 (0.1 , 0.2)	3.7 (2 , 6.1)	0.2 (0.1 , 0.3)	1.3 (0.95 , 1.64)
Mauritius	2 (1.4 , 2.8)	0.3 (0.2 , 0.4)	5.3 (3.5 , 7.5)	0.3 (0.2 , 0.4)	-0.65 (-1.08 , -0.21)
Mexico	56.4 (38.6 , 78.7)	0.1 (0.1 , 0.2)	68.6 (44.8 , 101.5)	0.1 (0 , 0.1)	-3.19 (-3.28 , -3.1)
Micronesia (Federated States of)	0.1 (0.1 , 0.2)	0.2 (0.1 , 0.4)	0.2 (0.1 , 0.3)	0.2 (0.1 , 0.3)	-0.5 (-0.55 , -0.46)
Monaco	0.4 (0.2 , 0.6)	0.6 (0.3 , 1)	0.6 (0.3 , 0.9)	0.6 (0.4 , 1)	0.34 (0.1 , 0.59)
Mongolia	11 (7 , 16.4)	1.1 (0.7 , 1.6)	20.3 (12.4 , 32.1)	0.9 (0.6 , 1.4)	-0.66 (-0.79 , -0.53)
Montenegro	2.1 (1.3 , 3.1)	0.3 (0.2 , 0.5)	3.3 (2 , 5)	0.3 (0.2 , 0.5)	0.33 (0.2 , 0.47)
Morocco	6.4 (4 , 9.8)	0 (0 , 0.1)	14.2 (8.7 , 22.1)	0 (0 , 0.1)	-0.34 (-0.6 , -0.09)
Mozambique	17.5 (10.8 , 27.2)	0.3 (0.2 , 0.5)	36.2 (22.8 , 56.2)	0.3 (0.2 , 0.5)	0.81 (0.59 , 1.02)
Myanmar	113.7 (68.4 , 169.7)	0.5 (0.3 , 0.7)	105.2 (65.1 , 159.3)	0.2 (0.1 , 0.3)	-3.04 (-3.19 , -2.89)
Namibia	0.8 (0.5 , 1.2)	0.1 (0.1 , 0.2)	1.4 (0.9 , 2.1)	0.1 (0.1 , 0.2)	-0.92 (-1.28 , -0.56)
Nauru	0 (0 , 0)	0.3 (0.2 , 0.5)	0 (0 , 0)	0.2 (0.1 , 0.3)	-1.3 (-1.46 , -1.13)
Nepal	59 (37.9 , 91)	0.6 (0.4 , 0.9)	101.9 (62.4 , 159)	0.4 (0.3 , 0.7)	-1.13 (-1.43 , -0.83)
Netherlands	140.8 (95.5 , 196.4)	0.7 (0.5 , 1)	260.3 (168.8 , 381.3)	0.7 (0.5 , 1.1)	0.33 (-0.01 , 0.68)
New Zealand	20 (13.3 , 28.8)	0.5 (0.3 , 0.7)	25.4 (16.2 , 37)	0.3 (0.2 , 0.4)	-1.82 (-1.99 , -1.65)
Nicaragua	0.8 (0.5 , 1.2)	0.1 (0 , 0.1)	2 (1.2 , 3.2)	0 (0 , 0.1)	-0.68 (-0.9 , -0.47)
Niger	2 (1.2 , 3.1)	0.1 (0 , 0.1)	8.3 (5 , 13.3)	0.1 (0.1 , 0.2)	1.62 (1.4 , 1.85)
Nigeria	20.9 (13.2 , 33.7)	0 (0 , 0.1)	53.8 (32.8 , 82.1)	0.1 (0 , 0.1)	0.96 (0.76 , 1.16)
Niue	0 (0 , 0)	0.2 (0.1 , 0.3)	0 (0 , 0)	0.2 (0.1 , 0.3)	-0.33 (-0.45 , -0.21)
North Macedonia	3.8 (2.4 , 5.6)	0.2 (0.1 , 0.3)	6.4 (3.9 , 9.6)	0.2 (0.1 , 0.3)	-0.21 (-0.49 , 0.08)
Northern Mariana Islands	0 (0 , 0)	0.1 (0.1 , 0.2)	0.1 (0.1 , 0.1)	0.2 (0.1 , 0.3)	2.63 (2.06 , 3.2)
Norway	20.4 (14.1 , 28.2)	0.3 (0.2 , 0.4)	25.8 (17.5 , 36.6)	0.3 (0.2 , 0.4)	-0.82 (-1.02 , -0.61)
Oman	0.8 (0.4 , 1.2)	0.1 (0.1 , 0.2)	1.7 (1 , 2.7)	0.1 (0.1 , 0.1)	-0.27 (-0.48 , -0.05)
Pakistan	320.3 (213 , 453.3)	0.6 (0.4 , 0.8)	591.2 (383 , 860.8)	0.5 (0.3 , 0.7)	-0.97 (-1.23 , -0.71)
Palau	0 (0 , 0)	0.2 (0.1 , 0.3)	0 (0 , 0.1)	0.2 (0.1 , 0.3)	-0.52 (-0.59 , -0.44)
Palestine	1.2 (0.7 , 1.8)	0.1 (0.1 , 0.2)	2.3 (1.5 , 3.6)	0.1 (0.1 , 0.2)	-1.63 (-1.83 , -1.42)
Panama	1.7 (1.1 , 2.5)	0.1 (0.1 , 0.2)	2.4 (1.5 , 3.8)	0.1 (0 , 0.1)	-2.57 (-2.68 , -2.46)
Papua New Guinea	2.1 (1.2 , 3.5)	0.1 (0.1 , 0.2)	4.5 (2.5 , 7.2)	0.1 (0 , 0.1)	-1.27 (-1.39 , -1.15)
Paraguay	8 (5.1 , 11.9)	0.4 (0.2 , 0.5)	19.8 (11.7 , 30.9)	0.3 (0.2 , 0.5)	-0.47 (-0.65 , -0.29)
Peru	5.5 (3.4 , 8.1)	0 (0 , 0.1)	11.9 (6.9 , 19.7)	0 (0 , 0.1)	-1.43 (-1.75 , -1.1)
Philippines	46 (30.4 , 65.5)	0.2 (0.1 , 0.2)	104.4 (69.2 , 148.1)	0.1 (0.1 , 0.2)	-0.79 (-0.97 , -0.61)
Poland	196.9 (135.3 , 265.7)	0.4 (0.3 , 0.6)	210.4 (140.9 , 294.5)	0.3 (0.2 , 0.4)	-1.37 (-1.53 , -1.21)
Portugal	59.7 (38.8 , 87.6)	0.4 (0.3 , 0.6)	58.9 (37.9 , 88.1)	0.3 (0.2 , 0.4)	-1.11 (-1.36 , -0.86)
Puerto Rico	14.5 (8.4 , 23.4)	0.4 (0.2 , 0.6)	10 (5.7 , 16.7)	0.1 (0.1 , 0.2)	-3.16 (-3.35 , -2.96)
Qatar	0.4 (0.2 , 0.6)	0.4 (0.2 , 0.6)	1.7 (0.9 , 2.7)	0.2 (0.1 , 0.3)	-2.45 (-3.06 , -1.84)
Republic of Korea	296.2 (189.1 , 429.1)	1 (0.6 , 1.4)	587 (384 , 840.8)	0.6 (0.4 , 0.9)	-1.72 (-1.83 , -1.62)
Republic of Moldova	15.2 (9.8 , 21.5)	0.3 (0.2 , 0.5)	17.6 (11.7 , 24.7)	0.3 (0.2 , 0.4)	-0.21 (-0.67 , 0.25)
Romania	45.9 (31.5 , 66.6)	0.2 (0.1 , 0.2)	90.4 (59 , 130)	0.3 (0.2 , 0.4)	1.29 (0.96 , 1.63)
Russian Federation	928.9 (640.3 , 1237.8)	0.5 (0.3 , 0.7)	914.8 (636.1 , 1280.7)	0.4 (0.3 , 0.5)	-0.98 (-1.15 , -0.82)
Rwanda	31.6 (19.8 , 48)	1.1 (0.7 , 1.7)	40.6 (23.7 , 64)	0.7 (0.4 , 1)	-2.52 (-2.88 , -2.17)
Saint Kitts and Nevis	0.1 (0 , 0.1)	0.2 (0.1 , 0.3)	0.1 (0.1 , 0.2)	0.1 (0.1 , 0.2)	-0.69 (-0.81 , -0.57)

Saint Lucia	0.3 (0.2 , 0.4)	0.3 (0.2 , 0.5)	0.5 (0.3 , 0.8)	0.2 (0.1 , 0.3)	-1.26 (-1.43 , -1.09)
Saint Vincent and the Grenadines	0.1 (0 , 0.1)	0.1 (0.1 , 0.1)	0.2 (0.1 , 0.2)	0.1 (0.1 , 0.2)	0.21 (-0.07 , 0.49)
Samoa	0.1 (0.1 , 0.1)	0.1 (0.1 , 0.2)	0.1 (0.1 , 0.2)	0.1 (0.1 , 0.1)	-0.91 (-1.02 , -0.8)
San Marino	0.1 (0 , 0.1)	0.2 (0.1 , 0.4)	0.1 (0 , 0.1)	0.1 (0.1 , 0.2)	-1.27 (-1.59 , -0.95)
Sao Tome and Principe	0 (0 , 0)	0 (0 , 0.1)	0.1 (0.1 , 0.2)	0.1 (0.1 , 0.2)	3.31 (3.11 , 3.51)
Saudi Arabia	7.6 (4.3 , 12.6)	0.1 (0.1 , 0.2)	24.9 (14.7 , 39.4)	0.1 (0.1 , 0.2)	-0.39 (-0.54 , -0.24)
Senegal	3.8 (2.4 , 5.6)	0.1 (0.1 , 0.2)	11.7 (6.8 , 18.8)	0.1 (0.1 , 0.2)	1.12 (0.83 , 1.41)
Serbia	31 (18.2 , 46.5)	0.3 (0.2 , 0.4)	40.6 (24.4 , 63.9)	0.3 (0.2 , 0.4)	-0.02 (-0.24 , 0.2)
Seychelles	0.3 (0.2 , 0.5)	0.5 (0.3 , 0.8)	0.6 (0.4 , 0.8)	0.5 (0.3 , 0.7)	-0.56 (-0.74 , -0.38)
Sierra Leone	2.4 (1.5 , 3.7)	0.1 (0.1 , 0.2)	6.4 (3.9 , 10)	0.2 (0.1 , 0.3)	1.74 (1.46 , 2.03)
Singapore	11 (6.8 , 16.1)	0.5 (0.3 , 0.7)	20.5 (12.6 , 30.4)	0.2 (0.1 , 0.3)	-2.35 (-2.63 , -2.06)
Slovakia	27.1 (15.9 , 41.4)	0.5 (0.3 , 0.7)	28.8 (17.3 , 46.3)	0.3 (0.2 , 0.5)	-1.23 (-1.33 , -1.13)
Slovenia	11.2 (7.4 , 16.5)	0.4 (0.3 , 0.7)	13.7 (8.7 , 19.9)	0.3 (0.2 , 0.5)	-1.04 (-1.19 , -0.89)
Solomon Islands	0.3 (0.2 , 0.5)	0.2 (0.1 , 0.4)	0.7 (0.4 , 1.1)	0.2 (0.1 , 0.3)	-0.43 (-0.55 , -0.31)
Somalia	16.4 (9.1 , 27.4)	0.6 (0.4 , 1)	24 (13.4 , 37.5)	0.4 (0.2 , 0.6)	-1.94 (-2.05 , -1.83)
South Africa	189.7 (128.2 , 263.7)	0.9 (0.6 , 1.2)	241.3 (163.6 , 338.5)	0.5 (0.3 , 0.7)	-2.52 (-2.96 , -2.08)
South Sudan	16.2 (9.5 , 26.5)	0.6 (0.4 , 1)	17.3 (9.3 , 26.2)	0.4 (0.2 , 0.6)	-1.57 (-1.79 , -1.35)
Spain	346.2 (227.8 , 510.9)	0.7 (0.4 , 1)	310.9 (198.6 , 449.2)	0.4 (0.2 , 0.5)	-1.96 (-2.19 , -1.74)
Sri Lanka	51.2 (32.2 , 76.2)	0.5 (0.3 , 0.7)	91.3 (51.1 , 139.8)	0.3 (0.2 , 0.5)	-0.77 (-1.03 , -0.52)
Sudan	30.6 (17.6 , 47.8)	0.3 (0.2 , 0.5)	64.4 (35.2 , 108.9)	0.3 (0.2 , 0.6)	0.05 (-0.01 , 0.11)
Suriname	0.4 (0.2 , 0.6)	0.1 (0.1 , 0.2)	0.8 (0.4 , 1.2)	0.1 (0.1 , 0.2)	-0.51 (-0.73 , -0.28)
Sweden	50.8 (34.1 , 72)	0.3 (0.2 , 0.5)	70.6 (45.4 , 105.6)	0.3 (0.2 , 0.5)	0.07 (-0.1 , 0.25)
Switzerland	65.6 (44.1 , 95)	0.7 (0.4 , 1)	70.3 (45.8 , 103)	0.4 (0.3 , 0.6)	-1.22 (-1.49 , -0.95)
Syrian Arab Republic	4.3 (2.8 , 6.4)	0.1 (0.1 , 0.1)	11 (6.4 , 17.4)	0.1 (0.1 , 0.1)	-0.18 (-0.35 , -0.01)
Taiwan (Province of China)	220.8 (148.2 , 310.2)	1.3 (0.9 , 1.9)	493.1 (332.7 , 708.9)	1.2 (0.8 , 1.7)	-0.34 (-0.77 , 0.09)
Tajikistan	31.7 (19.2 , 47)	1.1 (0.7 , 1.7)	26.2 (14.3 , 41.8)	0.5 (0.3 , 0.8)	-2.66 (-2.94 , -2.38)
Thailand	195.5 (118.1 , 315)	0.5 (0.3 , 0.9)	450.5 (280.8 , 692.7)	0.4 (0.3 , 0.6)	-1.27 (-1.41 , -1.14)
Timor-Leste	0.5 (0.3 , 0.8)	0.2 (0.1 , 0.3)	1.3 (0.8 , 2.1)	0.2 (0.1 , 0.2)	-0.73 (-0.95 , -0.51)
Togo	2.3 (1.3 , 3.6)	0.2 (0.1 , 0.3)	11.5 (6.8 , 18.2)	0.3 (0.2 , 0.4)	1.74 (1.55 , 1.92)
Tokelau	0 (0 , 0)	0.2 (0.1 , 0.3)	0 (0 , 0)	0.1 (0.1 , 0.2)	-0.98 (-1.07 , -0.89)
Tonga	0.1 (0.1 , 0.2)	0.2 (0.1 , 0.3)	0.2 (0.1 , 0.2)	0.2 (0.1 , 0.3)	-0.54 (-0.74 , -0.35)
Trinidad and Tobago	1.4 (0.9 , 1.9)	0.2 (0.1 , 0.2)	2.2 (1.3 , 3.4)	0.1 (0.1 , 0.2)	-1.08 (-1.29 , -0.87)
Tunisia	3.9 (2.4 , 5.6)	0.1 (0 , 0.1)	11.1 (6.5 , 18.3)	0.1 (0 , 0.1)	0 (-0.06 , 0.07)
Turkey	93.1 (58 , 135.8)	0.3 (0.2 , 0.4)	168 (102.7 , 250.6)	0.2 (0.1 , 0.3)	-1.49 (-1.72 , -1.27)
Turkmenistan	48.3 (32.4 , 68.2)	2.5 (1.6 , 3.4)	22.9 (13.4 , 35.4)	0.6 (0.3 , 0.9)	-4.82 (-5.27 , -4.36)
Tuvalu	0 (0 , 0)	0.2 (0.1 , 0.3)	0 (0 , 0)	0.2 (0.1 , 0.2)	-0.7 (-0.77 , -0.64)
Uganda	24.1 (14.7 , 35.9)	0.4 (0.2 , 0.5)	51.7 (31.1 , 80.4)	0.3 (0.2 , 0.5)	-0.92 (-1.3 , -0.54)
Ukraine	312.6 (212.5 , 445)	0.4 (0.3 , 0.6)	210.2 (114.1 , 349.9)	0.3 (0.2 , 0.5)	-1.62 (-1.74 , -1.5)
United Arab Emirates	0.7 (0.4 , 1.2)	0.2 (0.1 , 0.3)	3.7 (2.2 , 5.9)	0.1 (0.1 , 0.2)	0.05 (-0.46 , 0.58)
United Kingdom	885.4 (623.6 , 1180.7)	1 (0.7 , 1.3)	999.9 (678.1 , 1387.2)	0.8 (0.5 , 1.1)	-0.9 (-1.15 , -0.64)
United Republic of Tanzania	74.5 (44.4 , 111.2)	0.7 (0.4 , 1)	94.5 (57.9 , 151.2)	0.4 (0.2 , 0.6)	-2.51 (-2.69 , -2.32)

United States of America	2108.9 (1480.3 , 2864.2)	0.7 (0.5 , 0.9)	3271.5 (2241.4 , 4488.5)	0.6 (0.4 , 0.8)	-0.79 (-1.02 , -0.56)
United States Virgin Islands	0.2 (0.1 , 0.3)	0.2 (0.1 , 0.4)	0.2 (0.1 , 0.4)	0.1 (0.1 , 0.2)	-1.67 (-1.84 , -1.49)
Uruguay	28.9 (18.6 , 40)	0.7 (0.5 , 1)	23.9 (15.2 , 34.4)	0.5 (0.3 , 0.6)	-1.91 (-2.18 , -1.64)
Uzbekistan	64.2 (41.5 , 95.8)	0.5 (0.4 , 0.8)	46.6 (30.2 , 67.3)	0.2 (0.1 , 0.3)	-4.09 (-4.67 , -3.51)
Vanuatu	0.1 (0.1 , 0.2)	0.2 (0.1 , 0.2)	0.2 (0.1 , 0.3)	0.1 (0.1 , 0.2)	-1.31 (-1.42 , -1.21)
Venezuela (Bolivarian Republic of)	15.7 (10.4 , 21.9)	0.2 (0.1 , 0.2)	23.8 (13.8 , 37.2)	0.1 (0 , 0.1)	-2.6 (-2.74 , -2.45)
Viet Nam	94 (58.7 , 139.7)	0.2 (0.1 , 0.3)	281.9 (173.8 , 421.3)	0.3 (0.2 , 0.4)	0.57 (0.5 , 0.64)
Yemen	28.4 (16.2 , 45)	0.6 (0.3 , 0.9)	59 (31.4 , 96.2)	0.4 (0.2 , 0.7)	-1.21 (-1.37 , -1.05)
Zambia	17.8 (11 , 26.7)	0.6 (0.4 , 0.9)	31.3 (17.4 , 48.9)	0.5 (0.3 , 0.7)	-1.57 (-1.86 , -1.29)
Zimbabwe	37.2 (22.5 , 55)	0.9 (0.6 , 1.4)	64.3 (38.3 , 99.6)	0.9 (0.6 , 1.4)	-0.1 (-0.42 , 0.23)

sTable 4 Tobacco-Related Esophageal Cancer Years of Life Lost (YLLs) Between 1990 and 2021 for Both Sexes, Each SDI Regions and All countries, With EAPC Between 1990 and 2021.

	1990 YLLs		2021 YLLs		
	Case number (95% UI)	ASR/100,000 persons (95% UI)	Case number (95% UI)	ASR/100,000 persons (95% UI)	EAPC% (95% CI) 1990–2021
Global	3806182.4 (3105052.1 , 4543522.9)	92.4 (75.3 , 110.3)	5073074.8 (3987558.6 , 6269501.3)	57.8 (45.4 , 71.4)	-1.7 (-1.81 , -1.59)
**Sex**					
Male	3466555.6 (2801253.1 , 4165039.6)	177.9 (144.1 , 213.1)	4697091.8 (3660823.5 , 5826298)	113.4 (88.4 , 140.7)	-1.62 (-1.73 , -1.52)
Female	339626.8 (256675.6 , 421167.8)	16 (12.1 , 19.8)	375983 (288756.4 , 481016.4)	8.1 (6.3 , 10.4)	-2.47 (-2.62 , -2.32)
**SDI**					
High SDI	667648.6 (551973.7 , 774890)	62 (51.3 , 71.9)	750693.5 (597699.4 , 905917.1)	37.9 (30.4 , 45.6)	-1.72 (-1.86 , -1.57)
High-middle SDI	1251007.6 (991527.6 , 1527908.9)	121.4 (96.5 , 148.3)	1742109.1 (1309166.9 , 2276815.9)	86.5 (65 , 113)	-1.25 (-1.39 , -1.12)
Middle SDI	1542231.6 (1228770.3 , 1916601)	139.4 (111.4 , 172.9)	2013758.8 (1521211.6 , 2603570.6)	72.6 (54.7 , 93.9)	-2.32 (-2.43 , -2.21)
Low-middle SDI	265402.8 (221971.9 , 316446)	40.5 (33.7 , 48.2)	439344 (359081.8 , 525897.2)	29.1 (23.8 , 34.7)	-1.2 (-1.28 , -1.13)
Low SDI	78293.7 (63489.4 , 93683.2)	32.1 (26 , 38.3)	125265 (99500.5 , 154293.8)	23 (18.3 , 28.3)	-1.32 (-1.41 , -1.22)
**Country**					
Afghanistan	2011.8 (1151.3 , 3159.9)	27.6 (16.1 , 43.4)	2876.7 (1756.9 , 4478)	25.3 (15.5 , 38.2)	-0.43 (-0.78 , -0.08)
Albania	478.3 (353.5 , 622.4)	22.5 (16.7 , 29.1)	768.2 (516.6 , 1061.3)	17.7 (11.9 , 24.4)	-0.42 (-0.66 , -0.17)
Algeria	784.7 (586.7 , 1018)	6.6 (5 , 8.5)	2011.2 (1364.6 , 2780.2)	5.7 (3.9 , 7.9)	-0.5 (-0.56 , -0.44)
American Samoa	2.3 (1.6 , 3.1)	9.1 (6.4 , 12.5)	5.7 (3.9 , 7.7)	10.9 (7.7 , 14.7)	1.04 (0.71 , 1.37)
Andorra	13.8 (8.3 , 21.1)	23.4 (14.2 , 36)	16.9 (9 , 26.5)	11 (5.8 , 17.2)	-2.2 (-2.45 , -1.94)
Angola	2491 (1590.3 , 3636.7)	56.2 (36.2 , 81.4)	4721.1 (3234.8 , 6691.3)	35 (24.3 , 49.2)	-1.66 (-1.94 , -1.38)
Antigua and Barbuda	8.2 (5.8 , 10.9)	16.2 (11.6 , 21.3)	13.8 (9.8 , 18)	12.4 (8.8 , 16.3)	-0.73 (-1.01 , -0.45)
Argentina	20132.7 (15540.6 , 24442.8)	61.3 (47.4 , 74.6)	15363.2 (11506.2 , 19321.6)	28.1 (21.1 , 35.1)	-2.46 (-2.74 , -2.18)
Armenia	946.4 (798.9 , 1084.6)	32.6 (27.5 , 37.3)	519 (422.5 , 623.8)	11.5 (9.3 , 13.7)	-3.26 (-3.64 , -2.89)
Australia	7256.8 (5641.5 , 8973.9)	37 (28.8 , 45.7)	8089.3 (5842.1 , 10526.2)	18.9 (13.9 , 24.5)	-2.21 (-2.3 , -2.12)
Austria	3039.4 (2387.4 , 3711.9)	28.2 (22.2 , 34.4)	3827.3 (3036 , 4715.3)	23.2 (18.6 , 28.6)	-0.41 (-0.57 , -0.25)
Azerbaijan	3969.1 (2891.3 , 5073)	74.6 (54.2 , 95.3)	5658.8 (3928.1 , 7609.7)	52.4 (36.1 , 70.7)	-0.9 (-1.12 , -0.69)
Bahamas	59.5 (39 , 85.5)	37.4 (24.3 , 54.1)	120.3 (80 , 165.2)	27.5 (18.2 , 38.1)	-0.41 (-0.7 , -0.12)
Bahrain	58.5 (40 , 79.9)	36 (24.9 , 48.5)	138.2 (90 , 205.6)	16.9 (11.5 , 24.1)	-3.33 (-3.67 , -2.99)
Bangladesh	36231.8 (24846.4 , 51350)	73.4 (49.7 , 103.2)	57516.8 (38114.6 , 82621.6)	40.5 (27 , 58)	-1.81 (-1.94 , -1.68)
Barbados	79.9 (58.4 , 105.8)	28.3 (20.7 , 37.3)	91.9 (59.7 , 128.6)	17.8 (11.7 , 25)	-1.68 (-1.87 , -1.49)
Belarus	5163.8 (4162.7 , 6250.8)	38.9 (31.5 , 47.1)	6193.7 (4474.9 , 7984.7)	38.8 (28.1 , 49.7)	-0.45 (-0.62 , -0.27)
Belgium	7218.2 (5804.9 , 8554.1)	49.6 (40.2 , 58.8)	8511.6 (6536.8 , 10559.8)	39.8 (30.7 , 49.1)	-0.76 (-1.04 , -0.49)
Belize	9.9 (7.6 , 12.7)	10.8 (8.3 , 13.8)	33.6 (24.8 , 43.9)	10.5 (7.7 , 13.7)	0.32 (-0.16 , 0.79)
Benin	197.4 (143.9 , 270.8)	9.8 (7.2 , 13.4)	618.4 (423.4 , 853.7)	11.5 (7.9 , 15.7)	0.87 (0.7 , 1.04)
Bermuda	24 (16.7 , 33.3)	37.9 (26.2 , 52.5)	26.6 (18.5 , 35.9)	20.7 (14.3 , 27.9)	-1.53 (-1.83 , -1.23)
Bhutan	129.5 (84.9 , 183.8)	49.8 (32.7 , 70.4)	196.3 (132.7 , 281.8)	32.1 (21.8 , 46)	-1.48 (-1.69 , -1.27)
Bolivia (Plurinational State of)	343.6 (237.3 , 498.8)	10.6 (7.3 , 15.4)	648.7 (412.9 , 978.7)	7 (4.4 , 10.6)	-0.99 (-1.23 , -0.75)
Bosnia and Herzegovina	1244.8 (946.1 , 1599.9)	27.6 (20.9 , 35.3)	1651.3 (1129.4 , 2259)	27.2 (18.6 , 37.2)	0.11 (-0.08 , 0.31)
Botswana	551.1 (370.8 , 790.1)	91.5 (61.4 , 129.9)	926.2 (642.1 , 1295.2)	58.7 (40.9 , 82.2)	-1.8 (-2.12 , -1.48)
Brazil	76313.3 (61647.7 , 90827.9)	81.7 (65.7 , 97.7)	85755.3 (65322 , 107928.1)	33.3 (25.4 , 42)	-3.09 (-3.22 , -2.96)

Brunei Darussalam	37.1 (26.6 , 49.9)	37.6 (26.9 , 50.1)	66.6 (46.2 , 92.3)	18.1 (12.6 , 24.7)	-2.03 (-2.25 , -1.81)
Bulgaria	4282.8 (3431.9 , 5155.5)	34 (27.4 , 40.8)	3563.5 (2717.7 , 4485.6)	29 (22.3 , 36.2)	-0.74 (-0.96 , -0.51)
Burkina Faso	461.2 (330 , 626)	9.8 (7 , 13.4)	1669.7 (1097.1 , 2371.6)	16.4 (10.7 , 23.3)	2.27 (2.04 , 2.5)
Burundi	2190.5 (1519.9 , 2945.6)	88.8 (61.6 , 119.4)	1830.1 (1265.6 , 2527.1)	32.6 (22.7 , 44.5)	-3.69 (-3.94 , -3.44)
Cabo Verde	70.4 (52.3 , 92)	32.7 (24.5 , 43)	243.9 (168.4 , 331.5)	51.8 (36.3 , 71.1)	0.74 (0.31 , 1.17)
Cambodia	2615.8 (1941.6 , 3335.8)	55.4 (41.5 , 71)	4456.3 (3137 , 5948.3)	34.5 (24.6 , 45.6)	-1.83 (-1.94 , -1.71)
Cameroon	601.2 (397.8 , 842.5)	12.2 (8.2 , 16.9)	2795 (1724.9 , 4242.6)	19.6 (12.2 , 29.4)	1.99 (1.74 , 2.24)
Canada	15094.5 (12174.1 , 18025.5)	46.8 (37.9 , 55.8)	21950.1 (16708.2 , 27658.4)	31.2 (24 , 39.4)	-1.32 (-1.46 , -1.18)
Central African Republic	638.5 (422.1 , 902.7)	48 (32.5 , 67.6)	793.9 (518.1 , 1172.4)	29.4 (19.7 , 42.4)	-1.82 (-1.98 , -1.66)
Chad	250.8 (175.3 , 336.5)	8.7 (6.1 , 11.6)	1102 (710.5 , 1670.3)	17.9 (11.8 , 26.6)	2.74 (2.5 , 2.98)
Chile	4134.9 (3158.9 , 5180.5)	40.2 (30.5 , 50.3)	2686.9 (2051.4 , 3405)	10.6 (8.1 , 13.4)	-4.65 (-4.88 , -4.42)
China	2303268.8 (1786991.9 , 2891575.8)	258.4 (200.2 , 323.7)	3199109.5 (2325116.3 , 4210059.4)	145.3 (106.1 , 190.4)	-2.05 (-2.2 , -1.9)
Colombia	3658.3 (2854.8 , 4514.4)	20.3 (15.6 , 25.3)	2482.4 (1774.8 , 3338.5)	4.5 (3.2 , 6)	-5.46 (-5.7 , -5.22)
Comoros	233.2 (159.6 , 336.2)	108.7 (74.8 , 155.3)	358.3 (241.4 , 528.3)	68.5 (46.6 , 100.9)	-1.91 (-2.09 , -1.74)
Congo	598.4 (391.6 , 894.9)	51.4 (34 , 76.4)	1106 (721.4 , 1659.5)	36.8 (24.1 , 52.8)	-1.25 (-1.49 , -1)
Cook Islands	2.7 (1.8 , 3.8)	20.6 (13.7 , 28.9)	3.8 (2.7 , 5.4)	14.9 (10.4 , 21.1)	-1.13 (-1.23 , -1.03)
Costa Rica	310.6 (234.5 , 389.1)	18 (13.6 , 22.6)	404.7 (291.5 , 529.7)	7.3 (5.3 , 9.6)	-3.2 (-3.38 , -3.01)
Coted'Ivoire	200.8 (141.1 , 273.5)	4.4 (3.1 , 6.1)	541 (359.2 , 770.3)	4.3 (2.9 , 6.1)	-0.46 (-0.82 , -0.11)
Croatia	3514.7 (2833.6 , 4167.1)	54.3 (43.8 , 64.3)	2517.9 (1923.3 , 3125.2)	31.2 (23.9 , 38.8)	-1.69 (-1.88 , -1.49)
Cuba	3824.6 (3056 , 4610)	37.7 (30.1 , 45.4)	8937.5 (6693.5 , 11281.7)	45.9 (34.4 , 57.8)	0.99 (0.87 , 1.11)
Cyprus	111.2 (78.1 , 148.8)	14.2 (10 , 18.8)	275.8 (190.9 , 375.8)	13.5 (9.3 , 18.3)	0.55 (0.3 , 0.79)
Czechia	4721.4 (3743.8 , 5793.4)	35.3 (28 , 43.4)	6133.7 (4538.8 , 7809.8)	31.3 (23.2 , 40.1)	-0.54 (-0.68 , -0.4)
Democratic People's Republic of Korea	18052.7 (11814.1 , 25535.5)	99.6 (65.5 , 140.8)	30063.3 (20291.7 , 44031.8)	85.9 (58.3 , 125.1)	-0.51 (-0.63 , -0.39)
Democratic Republic of the Congo	4821.6 (3132.7 , 6710)	27.3 (17.9 , 37.9)	7886.6 (4874.9 , 11462.8)	18.6 (11.5 , 26.9)	-1.36 (-1.53 , -1.19)
Denmark	5023.7 (4138.8 , 5890.3)	66.5 (54.6 , 77.8)	5196.5 (4046.5 , 6354.5)	45.5 (35.4 , 55.4)	-1.47 (-1.63 , -1.31)
Djibouti	127 (80.1 , 197.2)	81.9 (51.2 , 123.5)	446.9 (266.2 , 694.3)	62.5 (38.6 , 95.2)	-0.94 (-1.05 , -0.83)
Dominica	11.1 (7.9 , 15)	19.2 (13.9 , 25.8)	15.5 (10.3 , 22.6)	17.6 (11.8 , 25.8)	-0.05 (-0.31 , 0.21)
Dominican Republic	593.4 (407 , 809.6)	16.2 (11.1 , 22.2)	1583.5 (1066.2 , 2274.1)	15.8 (10.5 , 22.6)	0.34 (0.16 , 0.51)
Ecuador	533.7 (413.9 , 666.7)	10.3 (8 , 12.9)	576.3 (401.6 , 787)	3.5 (2.5 , 4.8)	-2.93 (-3.25 , -2.62)
Egypt	2726.8 (2012.4 , 3558.6)	9.5 (7 , 12.3)	5820.6 (4225.4 , 7842.3)	9.1 (6.6 , 12.2)	-0.27 (-0.45 , -0.08)
El Salvador	162.6 (116.8 , 225.1)	5.4 (3.8 , 7.5)	339.2 (227.4 , 485)	5.6 (3.8 , 8)	-0.05 (-0.28 , 0.17)
Equatorial Guinea	93.8 (61.2 , 140.1)	42.4 (28.1 , 63)	163.6 (97.5 , 241.3)	28.6 (17.5 , 41.7)	-1.45 (-2.03 , -0.86)
Eritrea	901.7 (530.1 , 1319.4)	58.5 (35 , 85.2)	1054.5 (603.7 , 1642.4)	29.9 (17.8 , 46)	-2.69 (-2.91 , -2.48)
Estonia	779.7 (612.4 , 956.4)	37.9 (29.8 , 46.3)	628.3 (467.6 , 809.5)	26.8 (20.1 , 34.3)	-1.36 (-1.55 , -1.16)
Eswatini	179.9 (122 , 252.2)	58.8 (40.5 , 82.2)	282.4 (178 , 409.7)	45.7 (29.6 , 65.2)	-0.7 (-1.34 , -0.06)
Ethiopia	3462 (2351 , 5167.8)	15.7 (10.6 , 23.2)	2904.1 (2036.1 , 4045.7)	6.4 (4.6 , 9.1)	-3.01 (-3.32 , -2.69)
Fiji	62.4 (43.1 , 85.4)	15.6 (10.9 , 21.3)	110.2 (75.6 , 156.5)	12.9 (8.9 , 18.2)	-0.56 (-0.71 , -0.41)
Finland	1742.9 (1326.7 , 2218.4)	25.7 (19.7 , 32.5)	1910.4 (1411.7 , 2464.5)	17.4 (13.1 , 22.1)	-1.13 (-1.22 , -1.04)
France	69407.8 (54190.7 , 82746.9)	91.9 (72.4 , 109.5)	33961.9 (26504.4 , 42211.2)	27.7 (21.7 , 34.2)	-3.83 (-3.95 , -3.7)
Gabon	196.8 (137 , 277.8)	32.9 (22.8 , 46.4)	372.5 (249.1 , 529.1)	31.5 (21.4 , 43.8)	-0.25 (-0.36 , -0.14)
Gambia	26.1 (17.8 , 35.5)	6.9 (4.7 , 9.5)	68.4 (45.5 , 94.7)	6.5 (4.4 , 9)	-0.34 (-0.49 , -0.19)

Georgia	1475.6 (1142.2 , 1817.1)	22.5 (17.5 , 27.6)	675.8 (519.3 , 838.7)	11.7 (9 , 14.5)	-1.04 (-1.59 , -0.5)
Germany	50640.6 (39944.1 , 61148.4)	43.1 (34 , 52)	60179.9 (46528.6 , 73694.6)	35.2 (27.5 , 43)	-0.94 (-1.13 , -0.74)
Ghana	327.9 (225.3 , 439.7)	5 (3.5 , 6.7)	1175.3 (813.2 , 1591)	6.7 (4.6 , 9)	1.61 (1.38 , 1.84)
Greece	4066.5 (3369.9 , 4780.1)	26.6 (22.1 , 31.3)	4221.5 (3442.7 , 4992.4)	20.7 (16.9 , 24.4)	-0.95 (-1.11 , -0.8)
Greenland	86.9 (64.1 , 115.7)	231.3 (168.3 , 306.4)	88.2 (62.1 , 120.7)	113.4 (79.7 , 158.6)	-2.18 (-2.28 , -2.08)
Grenada	20.5 (14.7 , 27.7)	32.2 (23.2 , 43.4)	23.6 (17.3 , 30.6)	18.8 (13.9 , 24.3)	-1.28 (-1.79 , -0.76)
Guam	12.8 (9.6 , 16.6)	14.4 (10.7 , 19.1)	27.9 (20.4 , 36.3)	13.5 (9.9 , 17.6)	0.62 (0.26 , 0.97)
Guatemala	252.7 (191 , 326.9)	7.3 (5.4 , 9.7)	474.4 (335 , 637.8)	4.3 (3 , 5.8)	-2.02 (-2.39 , -1.66)
Guinea	126.6 (90.4 , 176.5)	3.7 (2.6 , 5.1)	251.2 (159.9 , 374.5)	4.2 (2.7 , 6.3)	0.7 (0.49 , 0.91)
Guinea-Bissau	40.7 (27 , 58.2)	9.4 (6.2 , 13.3)	157.8 (105 , 224)	18.6 (12.6 , 26.1)	3.24 (2.84 , 3.65)
Guyana	38.3 (28.1 , 49.6)	9.5 (7 , 12.4)	61.9 (40.4 , 90.6)	8.6 (5.6 , 12.6)	0.17 (-0.02 , 0.36)
Haiti	586.4 (393.4 , 870.3)	16.1 (10.8 , 23.8)	682 (429.4 , 1034)	8.3 (5.3 , 12.6)	-2.05 (-2.24 , -1.85)
Honduras	121.7 (85.5 , 167.9)	5.9 (4.1 , 8.1)	480.8 (328.1 , 675.3)	7.6 (5.2 , 10.6)	1.2 (1.01 , 1.4)
Hungary	8888.4 (7118.5 , 10591.7)	63.3 (50.9 , 75.7)	6342.4 (4897.1 , 8028.8)	36.9 (28.7 , 46.6)	-2.23 (-2.64 , -1.82)
Iceland	163.8 (130.3 , 196.1)	59.5 (47.3 , 71)	204.2 (151.2 , 260)	36.8 (27.3 , 46.5)	-1.72 (-1.88 , -1.56)
India	199651.5 (162301.4 , 243193.8)	38.7 (31.5 , 47.3)	352096.9 (273522.8 , 439092.8)	28.1 (21.9 , 34.9)	-1.34 (-1.46 , -1.22)
Indonesia	10945.8 (8348 , 13732.3)	10.5 (8 , 13.2)	26875.6 (19794.2 , 34873.9)	10.4 (7.8 , 13.5)	0.01 (-0.1 , 0.12)
Iran (Islamic Republic of)	8429.4 (6120.4 , 11046.5)	30.6 (22.1 , 40.1)	17448.8 (13204.2 , 21905.9)	22.3 (16.9 , 28.1)	-0.9 (-1.07 , -0.74)
Iraq	1030.8 (734.2 , 1404)	12.9 (9.2 , 17.7)	2691.5 (1700.6 , 3877.9)	11.1 (7.1 , 15.9)	-0.96 (-1.13 , -0.79)
Ireland	3646.2 (2921.1 , 4390.3)	89.6 (71.9 , 107.9)	3028.1 (2269.1 , 3925.9)	38.5 (28.9 , 49.8)	-2.63 (-2.77 , -2.49)
Israel	898.6 (712.5 , 1115.1)	18.9 (15 , 23.5)	1220.3 (932.5 , 1550.1)	10.3 (7.9 , 13.1)	-2.11 (-2.31 , -1.92)
Italy	31882.3 (25453.4 , 38513.5)	37.2 (29.8 , 44.7)	16709.6 (12933.8 , 20945)	12.8 (10.1 , 15.9)	-3.41 (-3.47 , -3.34)
Jamaica	332.9 (250.7 , 420.4)	19.3 (14.6 , 24.2)	511.2 (342.3 , 753.5)	16.6 (11.1 , 24.5)	-0.75 (-1.29 , -0.21)
Japan	109126.3 (91213.9 , 125776.5)	62.6 (52.3 , 72.2)	105730.1 (83342.1 , 129091.7)	32.6 (26.1 , 39.3)	-2.36 (-2.59 , -2.13)
Jordan	171.4 (128.6 , 225.1)	12.3 (9.2 , 16)	707.4 (506.3 , 942.3)	9.1 (6.5 , 11.9)	-1.16 (-1.34 , -0.97)
Kazakhstan	18308.7 (13753 , 23212.3)	136.8 (102.2 , 173.8)	5511.4 (4250.7 , 7006.8)	28.5 (21.8 , 36.4)	-5 (-5.28 , -4.73)
Kenya	2703.7 (1927.3 , 3917)	31.5 (22.4 , 45.7)	8163.4 (5790.3 , 11871.6)	32.8 (23.2 , 47.5)	0.11 (-0.13 , 0.35)
Kiribati	24.9 (18.1 , 32.6)	62.3 (44.7 , 82.4)	46.5 (31.5 , 65.1)	57.1 (39.3 , 79.6)	-0.46 (-0.64 , -0.29)
Kuwait	95.9 (69.5 , 126.1)	14.7 (10.6 , 19.2)	209.6 (149.2 , 275.5)	7.4 (5.2 , 9.7)	-2.63 (-3.45 , -1.8)
Kyrgyzstan	2861.6 (2264.3 , 3521.4)	91.6 (72.8 , 112.6)	1510.6 (1149.7 , 1949.5)	29.8 (22.9 , 38.3)	-3.43 (-3.57 , -3.29)
Lao People's Democratic Republic	970.9 (649.6 , 1376.8)	44.1 (29.7 , 62.1)	1102.5 (796 , 1553.6)	22.9 (16.5 , 32)	-2.2 (-2.29 , -2.11)
Latvia	1309.6 (1082.7 , 1588.2)	36.5 (30.2 , 44.2)	1045.5 (786.4 , 1288.1)	30.9 (23.2 , 37.9)	-0.52 (-0.74 , -0.3)
Lebanon	332 (236.8 , 458.7)	14.6 (10.5 , 20.2)	628.6 (461.9 , 842.1)	10.7 (7.8 , 14.4)	-0.58 (-0.78 , -0.37)
Lesotho	657.5 (432.2 , 961.1)	76.6 (50 , 112.1)	1500.2 (1037 , 2085.8)	130.5 (90.8 , 180.3)	2.36 (1.98 , 2.75)
Liberia	94.2 (68 , 126.6)	7.9 (5.7 , 10.7)	340.4 (220.9 , 504.4)	13.9 (9 , 20.5)	2.45 (2.12 , 2.78)
Libya	315.2 (204 , 474.8)	16.2 (10.5 , 24.3)	1084.6 (707.1 , 1558.2)	19.3 (12.6 , 27.2)	0.92 (0.76 , 1.08)
Lithuania	1526.5 (1188.2 , 1870.9)	33.6 (26.2 , 41.1)	1760.8 (1360.7 , 2180.7)	35 (27.2 , 43.2)	0.19 (-0.04 , 0.43)
Luxembourg	287.4 (206.7 , 382.2)	54.4 (39.2 , 72.3)	298.1 (208.2 , 407)	29 (20.3 , 39.5)	-2.16 (-2.36 , -1.96)
Madagascar	5065.3 (3518.4 , 7032.5)	89.9 (62.7 , 124)	7332.2 (4608.2 , 10926.7)	50.7 (32.2 , 75.1)	-1.9 (-1.97 , -1.82)
Malawi	3368.4 (2497.6 , 4363.1)	83.9 (61.7 , 108.2)	8922 (6219.1 , 12208.2)	112.1 (79.1 , 152.3)	0.88 (0.38 , 1.37)
Malaysia	1984.9 (1503.8 , 2531.5)	21.2 (16.2 , 27.1)	5338.3 (4027.7 , 6875.8)	18.2 (13.8 , 23.3)	-0.73 (-0.9 , -0.56)
Maldives	30.8 (21.3 , 42.3)	31.6 (21.9 , 43)	39.5 (28.9 , 51.8)	11.3 (8.3 , 14.6)	-3.84 (-4.12 , -3.57)

Mali	281.7 (204.7 , 368.3)	6.6 (4.8 , 8.6)	791 (533.5 , 1142.2)	8.4 (5.6 , 12)	1.27 (1.08 , 1.46)
Malta	142.6 (110.1 , 176.2)	33.1 (25.6 , 40.9)	173.6 (129.7 , 222.6)	20.6 (15.6 , 26.4)	-1.4 (-1.51 , -1.29)
Marshall Islands	3 (1.8 , 4.7)	16.8 (9.9 , 27)	6.2 (3.8 , 9.6)	15.2 (9.3 , 23.1)	-0.13 (-0.24 , -0.02)
Mauritania	130.6 (86.5 , 186.1)	12.4 (8.2 , 17.6)	385.5 (229.8 , 592.2)	16.6 (10 , 25.5)	1.35 (0.97 , 1.72)
Mauritius	196 (159.2 , 233.9)	25.8 (20.9 , 30.7)	489 (396.4 , 581.5)	25.5 (20.7 , 30.3)	-0.79 (-1.3 , -0.27)
Mexico	5279.6 (4156.3 , 6389.9)	12.7 (9.9 , 15.5)	6107.9 (4554.5 , 7703.9)	4.7 (3.5 , 6)	-3.27 (-3.35 , -3.18)
Micronesia (Federated States of)	13.2 (9.1 , 18.4)	25.4 (17.6 , 35)	18.6 (12 , 26.4)	21.3 (14.1 , 30.2)	-0.65 (-0.68 , -0.61)
Monaco	29.8 (19.1 , 42.2)	48.8 (31.5 , 68.5)	37.6 (24 , 56.8)	43.8 (28.4 , 66.7)	-0.26 (-0.49 , -0.03)
Mongolia	1175.4 (855.3 , 1598.7)	111.7 (81.4 , 151.1)	2206.4 (1537.2 , 3021.2)	92 (64.3 , 122.8)	-0.73 (-0.88 , -0.58)
Montenegro	201.2 (144.8 , 267.6)	30.4 (21.9 , 40.4)	298.7 (207.7 , 406.5)	30.1 (20.9 , 40.9)	0.14 (-0.02 , 0.31)
Morocco	612.9 (428.3 , 825.6)	4.2 (2.9 , 5.7)	1294.2 (842.5 , 1817.8)	3.6 (2.3 , 5)	-0.51 (-0.75 , -0.26)
Mozambique	1864.8 (1350.5 , 2488.4)	30.7 (22.2 , 40.7)	3970.2 (2768.7 , 5362.6)	34 (23.8 , 45.5)	0.95 (0.7 , 1.19)
Myanmar	11861.8 (8438.4 , 16135.7)	47.7 (34.1 , 64.6)	10120.2 (7223.9 , 14177.7)	19.4 (13.8 , 27.2)	-3.29 (-3.43 , -3.14)
Namibia	79.9 (58.5 , 108.3)	12 (8.8 , 16)	140.3 (97.9 , 191.6)	9.7 (7 , 13)	-0.97 (-1.36 , -0.57)
Nauru	1.5 (1 , 2.2)	29.4 (18.4 , 42)	1.5 (1 , 2.1)	21.9 (14.3 , 30.8)	-1.3 (-1.48 , -1.11)
Nepal	6292.6 (4520.7 , 8547.2)	62.5 (45.1 , 84.2)	9900.3 (6726 , 14275.9)	41.3 (28.3 , 58.8)	-1.33 (-1.68 , -0.98)
Netherlands	11899.7 (9483.7 , 14080.7)	61.7 (49.3 , 72.9)	17151.5 (13010.8 , 21303.9)	49.1 (37.6 , 60.7)	-0.69 (-1 , -0.39)
New Zealand	1509.3 (1155.4 , 1843.2)	38.4 (29.4 , 46.8)	1433.8 (1089.9 , 1839.2)	17.1 (13.1 , 21.8)	-2.81 (-2.99 , -2.64)
Nicaragua	77 (56.1 , 100.4)	5 (3.7 , 6.5)	178.8 (121.9 , 252.1)	3.6 (2.5 , 5.1)	-0.87 (-1.06 , -0.67)
Niger	210.5 (140.1 , 298.5)	6.9 (4.6 , 9.7)	876.6 (567.7 , 1315.5)	9.8 (6.6 , 14.5)	1.83 (1.58 , 2.08)
Nigeria	2117.4 (1437.6 , 3126)	4.5 (3.1 , 6.7)	5757.7 (3796.5 , 8247.6)	5.6 (3.8 , 8)	1.14 (0.92 , 1.37)
Niue	0.3 (0.2 , 0.5)	15.8 (10.4 , 23.5)	0.3 (0.2 , 0.5)	15.2 (10.1 , 22.8)	-0.45 (-0.58 , -0.32)
North Macedonia	378.5 (274.7 , 484.2)	19 (13.8 , 24.4)	589.4 (404.1 , 810.4)	17.2 (11.8 , 23.6)	-0.38 (-0.67 , -0.08)
Northern Mariana Islands	2 (1.4 , 2.8)	9.6 (6.8 , 13.7)	9.2 (6.8 , 12.2)	15.7 (11.6 , 21)	2.71 (2.12 , 3.3)
Norway	1573.2 (1273.3 , 1888.6)	25.2 (20.4 , 30)	1638.7 (1237.4 , 2070.4)	17.3 (13.2 , 21.6)	-1.57 (-1.79 , -1.35)
Oman	73 (45.9 , 108.1)	10.4 (6.5 , 15.3)	149.3 (97.3 , 216.1)	7 (4.7 , 10.3)	-0.78 (-0.96 , -0.6)
Pakistan	33379.9 (26301.5 , 40816.4)	58 (45.6 , 70.7)	62451.2 (46500.8 , 83115.8)	48.2 (36.2 , 63.9)	-1.02 (-1.31 , -0.74)
Palau	2.4 (1.7 , 3.4)	22.8 (15.9 , 31.5)	5 (3.6 , 6.9)	19.6 (14.3 , 27.2)	-0.57 (-0.64 , -0.49)
Palestine	105.3 (69.4 , 153)	12.5 (8.3 , 18)	197.2 (135.4 , 275.4)	7.9 (5.4 , 11)	-1.86 (-2.08 , -1.63)
Panama	147.3 (112.1 , 189.2)	10.1 (7.7 , 13)	196.1 (130.5 , 275.4)	4.4 (3 , 6.2)	-2.74 (-2.87 , -2.62)
Papua New Guinea	229 (137.8 , 362.1)	10.7 (6.5 , 16.7)	480.1 (318.1 , 725.1)	7.7 (5.1 , 11.8)	-1.32 (-1.44 , -1.2)
Paraguay	776.9 (573.8 , 1009.6)	34.9 (25.7 , 45.1)	1912.4 (1277.1 , 2800)	32.3 (21.5 , 46.9)	-0.53 (-0.73 , -0.34)
Peru	501.5 (351.6 , 688.9)	4.2 (3 , 5.8)	991.2 (658.4 , 1498.3)	3 (2 , 4.5)	-1.7 (-2.04 , -1.36)
Philippines	4697.8 (3682.1 , 5950.4)	14.6 (11.5 , 18.5)	10179.8 (7653.9 , 13038.8)	11.5 (8.6 , 14.6)	-0.89 (-1.08 , -0.7)
Poland	20546.6 (17063.6 , 24081.5)	47 (39.1 , 55.1)	20385.1 (16078.5 , 25029)	30.6 (24.2 , 37.4)	-1.59 (-1.78 , -1.4)
Portugal	5851.8 (4451.9 , 7371.9)	42.7 (32.6 , 53.5)	5026.9 (3840.4 , 6282.8)	25.6 (19.7 , 31.8)	-1.48 (-1.73 , -1.23)
Puerto Rico	1361.5 (912.8 , 1980.5)	37.7 (25.3 , 54.8)	794.7 (514.6 , 1225.8)	12.5 (8.2 , 18.8)	-3.49 (-3.69 , -3.28)
Qatar	37.6 (23.1 , 56.2)	36.7 (22.7 , 52.5)	139.7 (82.3 , 208.2)	14.5 (8.3 , 22.4)	-3.25 (-3.92 , -2.57)
Republic of Korea	27759.5 (20728.3 , 35202.4)	86.2 (64.6 , 109.6)	22761.1 (16078.2 , 30630.9)	23.6 (16.7 , 31.9)	-4.71 (-4.91 , -4.52)
Republic of Moldova	1555.4 (1150.7 , 1976.9)	33.1 (24.6 , 41.8)	1633.9 (1271.3 , 2023.7)	27.2 (21.1 , 33.6)	-0.46 (-0.89 , -0.02)
Romania	4728.2 (3720.1 , 5813.7)	16.4 (13 , 20.2)	8645.8 (6682.6 , 10824.2)	26.9 (20.9 , 33.4)	1.13 (0.77 , 1.49)
Russian Federation	98341.3 (81389.6 , 114287.4)	52.2 (43.2 , 60.6)	84241 (67560.2 , 101523.6)	35.6 (28.6 , 42.9)	-1.46 (-1.61 , -1.3)

Rwanda	3558.4 (2275.5 , 4875.6)	118.5 (77.3 , 161.9)	4253.5 (2866.9 , 6001.1)	65.6 (44.8 , 92.5)	-2.96 (-3.36 , -2.55)
Saint Kitts and Nevis	6.5 (4.4 , 9.2)	18.5 (12.8 , 25.7)	10 (6.9 , 13.6)	13.3 (9.1 , 18.2)	-0.81 (-0.95 , -0.67)
Saint Lucia	26.6 (19.7 , 33.9)	30.8 (22.9 , 39.3)	51.4 (35.6 , 70)	20.7 (14.4 , 28.2)	-1.33 (-1.52 , -1.14)
Saint Vincent and the Grenadines	7.1 (5.2 , 8.9)	10.2 (7.6 , 12.8)	15.6 (11.4 , 20.7)	10.6 (7.8 , 14.1)	0.27 (-0.02 , 0.55)
Samoa	9.1 (6.4 , 11.9)	10.1 (7.2 , 13.1)	12.2 (8.8 , 16.4)	8 (5.8 , 10.8)	-1 (-1.12 , -0.89)
San Marino	6.2 (4.2 , 8.7)	18.3 (12.4 , 25.7)	5.3 (3 , 8.5)	7.9 (4.3 , 13)	-1.87 (-2.17 , -1.57)
Sao Tome and Principe	2.9 (2.1 , 4.1)	4.4 (3.1 , 6)	13.5 (9 , 19.3)	10.8 (7.2 , 15.2)	3.44 (3.22 , 3.65)
Saudi Arabia	735.6 (444.2 , 1168.8)	12.3 (7.3 , 19.3)	2289.8 (1443.2 , 3328.2)	10.7 (6.7 , 15.6)	-0.77 (-0.92 , -0.63)
Senegal	399.8 (283.1 , 554)	11.4 (8.1 , 15.8)	1258.6 (852 , 1852.8)	14.8 (10 , 21.5)	1.25 (0.94 , 1.55)
Serbia	3131.7 (2076.9 , 4483.7)	25.6 (17.2 , 36.6)	3596.4 (2334.6 , 5302.6)	23.6 (15.3 , 34.9)	-0.31 (-0.55 , -0.07)
Seychelles	31.3 (22.7 , 41.4)	56.4 (41 , 74.3)	54 (40.1 , 71.1)	43.4 (32.7 , 56.8)	-0.8 (-0.97 , -0.62)
Sierra Leone	246.5 (173.7 , 339)	11.8 (8.3 , 16.1)	688.9 (481.4 , 982.7)	16.7 (11.7 , 23.7)	1.97 (1.65 , 2.29)
Singapore	864.5 (634.8 , 1122)	38.5 (27.9 , 50.1)	810.5 (597.2 , 1040.5)	9.1 (6.7 , 11.7)	-4.57 (-4.89 , -4.25)
Slovakia	2855.7 (1951.8 , 3976.8)	48.9 (33.7 , 67.7)	2750.7 (1849.4 , 3947.4)	30.2 (20.2 , 43.7)	-1.55 (-1.67 , -1.43)
Slovenia	1128.2 (870.5 , 1390.9)	45.2 (34.9 , 55.6)	971.6 (705.2 , 1233.9)	24.6 (17.8 , 31.4)	-2.11 (-2.29 , -1.94)
Solomon Islands	34.6 (21.2 , 50.7)	22.6 (14.3 , 32.9)	77.6 (48.8 , 116.6)	19.6 (12.6 , 29.2)	-0.4 (-0.52 , -0.29)
Somalia	1955 (1162.5 , 2986.5)	69.5 (42 , 104.9)	2816.6 (1787.1 , 4340.9)	40.1 (26.4 , 60.3)	-2.05 (-2.18 , -1.93)
South Africa	21115.7 (16672.7 , 26352.2)	95.6 (75.2 , 119.5)	26517.9 (20623.6 , 33206.4)	53.3 (41.6 , 66.3)	-2.58 (-3.05 , -2.11)
South Sudan	1801.1 (1179.3 , 2653)	67.6 (44.2 , 99.7)	1982.7 (1224.8 , 2912.1)	45.3 (28.9 , 66.9)	-1.65 (-1.89 , -1.41)
Spain	30396.4 (24909.7 , 35920.6)	59 (48.5 , 69.5)	20836.9 (16016 , 25177.2)	24.2 (18.7 , 29.2)	-2.91 (-3.07 , -2.74)
Sri Lanka	5180.6 (3948.3 , 6725.6)	46.2 (35.6 , 59.7)	7925.1 (4724.2 , 11522.1)	28.3 (17 , 41)	-1.17 (-1.46 , -0.89)
Sudan	3120.4 (2019.6 , 4518.4)	32.8 (21.3 , 47.5)	6292.1 (3790.2 , 9591.2)	32 (19.6 , 48.9)	-0.18 (-0.24 , -0.13)
Suriname	36.9 (27.2 , 49.7)	13.6 (10.1 , 18.4)	76.5 (51.1 , 109.6)	11.3 (7.6 , 16.2)	-0.48 (-0.7 , -0.25)
Sweden	3700 (2947.5 , 4480.9)	25.7 (20.5 , 30.9)	4508.2 (3315.8 , 5798)	21.9 (16.2 , 28.1)	-0.38 (-0.55 , -0.22)
Switzerland	5308.4 (4251.8 , 6400.7)	54.7 (44.2 , 66.2)	4299 (3303.1 , 5323.1)	24.8 (19.1 , 30.7)	-2.25 (-2.43 , -2.07)
Syrian Arab Republic	409.2 (288.4 , 560.7)	7.7 (5.5 , 10.4)	928.1 (595.9 , 1326.5)	7 (4.5 , 9.8)	-0.61 (-0.78 , -0.44)
Taiwan (Province of China)	21010.7 (16966.4 , 24875.9)	124.1 (101.1 , 146.7)	37307.3 (29574.8 , 45277.6)	91.7 (72.5 , 110.9)	-0.96 (-1.31 , -0.61)
Tajikistan	3323.8 (2338.6 , 4430.6)	117.5 (82.7 , 154.9)	2656.9 (1598.4 , 4164.8)	45.7 (27.5 , 71.5)	-2.96 (-3.22 , -2.69)
Thailand	20143.7 (14307.8 , 29174.7)	53.2 (37.9 , 76.3)	40382.1 (29053.5 , 56263.2)	36.6 (26.4 , 51)	-1.57 (-1.71 , -1.43)
Timor-Leste	55.6 (33.8 , 82.3)	18.1 (11.3 , 26.8)	121.9 (79.4 , 172.5)	14 (9.1 , 19.9)	-0.85 (-1.09 , -0.61)
Togo	229.4 (151 , 334.2)	17.8 (11.8 , 26.2)	1235.6 (801.7 , 1819.5)	29 (18.8 , 42.3)	2.05 (1.84 , 2.25)
Tokelau	0.2 (0.1 , 0.3)	14.9 (8.9 , 22.5)	0.2 (0.1 , 0.2)	11.5 (7.6 , 16.1)	-1.06 (-1.14 , -0.97)
Tonga	11.1 (7.7 , 14.9)	19.4 (13.5 , 26.4)	14.1 (9.8 , 20.2)	17.5 (12.1 , 24.9)	-0.57 (-0.79 , -0.34)
Trinidad and Tobago	131.9 (102.1 , 162.7)	15.6 (12.1 , 19.4)	209.4 (139.2 , 295.2)	10.7 (7.1 , 15)	-1.14 (-1.35 , -0.92)
Tunisia	346.7 (249.1 , 466.4)	6.8 (4.9 , 9)	882.3 (539.2 , 1310.9)	6.5 (4 , 9.5)	-0.33 (-0.4 , -0.26)
Turkey	9209.5 (6668.3 , 12375.5)	25 (18.2 , 33.3)	13641.1 (9255.7 , 18511.3)	14.1 (9.6 , 19.1)	-2.12 (-2.35 , -1.89)
Turkmenistan	5373.2 (4367.2 , 6396.4)	266 (217.6 , 314)	2365.7 (1615 , 3250.9)	55.9 (38.5 , 77)	-5.14 (-5.6 , -4.67)
Tuvalu	1.4 (1 , 2)	19.2 (13.3 , 26.6)	1.7 (1.2 , 2.4)	15.2 (10.6 , 21.4)	-0.81 (-0.87 , -0.74)
Uganda	2674.2 (1894.9 , 3600.3)	39.5 (27.9 , 53)	5739 (4084 , 7909.5)	35.9 (25.6 , 49.4)	-1.04 (-1.45 , -0.63)
Ukraine	30876.3 (24929 , 36869.7)	42.7 (34.6 , 51.1)	19315.6 (12033.4 , 28872.8)	26.5 (16.5 , 39.6)	-1.9 (-2.05 , -1.74)
United Arab Emirates	72.6 (47.4 , 112.8)	15.4 (9.8 , 24.2)	372.3 (248.8 , 531.1)	9.9 (6.5 , 13.9)	-0.43 (-1.02 , 0.17)

United Kingdom	73099 (58407.5 , 85775.4)	83.6 (66.9 , 97.8)	70016.3 (52866.8 , 86928.9)	55.2 (42 , 68.4)	-1.47 (-1.7 , -1.24)
United Republic of Tanzania	8211 (5941.3 , 11069)	71.2 (51.8 , 97)	10218.4 (6977.6 , 14677.4)	37.1 (25.4 , 53.2)	-2.64 (-2.84 , -2.43)
United States of America	166836.9 (136951.6 , 193367.3)	55.8 (45.8 , 64.6)	228599.6 (177970 , 275702.3)	39.9 (31.2 , 48.1)	-1.19 (-1.37 , -1.01)
United States Virgin Islands	17.7 (10.3 , 28.1)	19.8 (11.4 , 31.2)	19.9 (10.9 , 32.9)	11.2 (6.4 , 18.3)	-1.78 (-1.96 , -1.61)
Uruguay	2853.5 (2232.3 , 3511)	74 (58 , 91.1)	2118.3 (1634.3 , 2618.4)	41.5 (32 , 51)	-2.23 (-2.51 , -1.95)
Uzbekistan	7063.2 (5362.1 , 8903.9)	58.8 (44.5 , 74.1)	4787.4 (3586.8 , 6174.7)	17 (12.7 , 22.1)	-4.44 (-5.04 , -3.84)
Vanuatu	10.3 (7 , 15.1)	14.7 (10.2 , 21.7)	20.6 (14.4 , 28.5)	10.5 (7.3 , 14.5)	-1.31 (-1.42 , -1.21)
Venezuela (Bolivarian Republic of)	1507.7 (1182 , 1892.4)	15.6 (12.1 , 19.6)	2141.7 (1301.2 , 3047.1)	6.9 (4.2 , 9.9)	-2.77 (-2.92 , -2.62)
Viet Nam	9054.4 (6251.5 , 12745.2)	21.9 (15.2 , 30.5)	25972.6 (17935.1 , 35216.3)	23.8 (16.7 , 32)	0.42 (0.35 , 0.49)
Yemen	3006.4 (2000.6 , 4301.6)	57 (38.4 , 80.9)	5885.8 (3620.7 , 8525.7)	40.3 (25.4 , 57.6)	-1.49 (-1.65 , -1.33)
Zambia	1975.9 (1403.6 , 2695.9)	66.7 (47.6 , 90.3)	3487.7 (2300.8 , 4927.5)	47.5 (32.1 , 66.9)	-1.7 (-2.02 , -1.38)
Zimbabwe	4000.2 (2754.4 , 5460.6)	95.2 (65.6 , 130.2)	7233 (4901.7 , 9798.5)	97.3 (67.3 , 128.7)	-0.03 (-0.39 , 0.33)

© 2025 Chen J. et al.
